# Genome‐Wide Association Studies Reveal the Genetic Architecture of Ionomic Variation in Grains of Tartary Buckwheat

**DOI:** 10.1002/advs.202412291

**Published:** 2025-03-13

**Authors:** Zhirong Wang, Yuqi He, Mengyu Zhao, Xiang‐Qian Liu, Hao Lin, Yaliang Shi, Kaixuan Zhang, Guijie Lei, Dili Lai, Tong Liu, Xiaoyang Peng, Jiayue He, Wei Li, Xiangru Wang, Sun‐Hee Woo, Muriel Quinet, Alisdair R. Fernie, Xin‐Yuan Huang, Meiliang Zhou

**Affiliations:** ^1^ National Key Facility for Crop Gene Resources and Genetic Improvement/Key laboratory Grain Crop Genetic Resources Evaluation and Utilization Ministry of Agriculture and Rural Affairs Institute of Crop Sciences Chinese Academy of Agricultural Sciences Beijing 100081 P. R. China; ^2^ State Key Laboratory of Crop Genetics & Germplasm Enhancement and Utilization College of Resources and Environmental Sciences Nanjing Agricultural University Nanjing 210095 P. R. China; ^3^ State Key Laboratory for Conservation and Utilization of Bio‐Resources in Yunnan Key Laboratory of Biology and Germplasm Innovation of Perennial Rice School of Agriculture Yunnan University Kunming 650500 P. R. China; ^4^ Department of Crop Science Chungbuk National University Cheong‐ju 28644 Republic of Korea; ^5^ Groupe de Recherche en Physiologie Végétale (GRPV) Earth and Life Institute‐Agronomy (ELI‐A) Université catholique de Louvain Croix du Sud 45, boîte L7.07.13 Louvain‐la‐Neuve B‐1348 Belgium; ^6^ Department of Molecular Physiology Max‐Planck‐Institute of Molecular Plant Physiology 14476 Potsdam Germany; ^7^ Sanya Institute of Nanjing Agricultural University Sanya 572024 P. R. China

**Keywords:** biofortification, Fagopyrum tataricum, Genetic basis, ionomic variation

## Abstract

Tartary buckwheat (*Fagopyrum tataricum*) is esteemed as a medicinal crop due to its high nutritional and health value. However, the genetic basis for the variations in Tartary buckwheat grain ionome remains inadequately understood. Through genome‐wide association studies (GWAS) on grain ionome, 52 genetic loci are identified associated with 10 elements undergoing selection. Molecular experiments have shown that the variation in *FtACA13*’s promoter (an auto‐inhibited Ca^2+^‐ATPase) is accountable for grain sodium concentration and salt tolerance, which underwent selection during domestication. *FtYPQ1* (a vacuolar amino acid transporter) exhibits zinc transport activity, enhancing tolerance to excessive zinc stress and raising zinc accumulation. Additionally, *FtNHX2* (a Na^+^/H^+^ exchanger) positively regulates arsenic content. Further genomic comparative analysis of “20A1” (wild accession) and “Pinku” (cultivated accession) unveiled structural variants in key genes involved in ion uptake and transport that may result in considerable changes in their functions. This research establishes the initial comprehensive grain ionome atlas in Tartary buckwheat, which will significantly aid in genetic improvement for nutrient biofortification.

## Graphical abstract

In this study, variation and genetic basis of ionome among Tartary buckwheat population are investigated, illuminating their significance in the domestication and diversification of Tartary buckwheat. And three genes (*FtACA13* for Na content, *FtYPQ1* for Zn content, *FtNHX2* for As content) are identified with the dominant haplotypes and functional validation in detail.

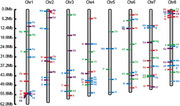

## 1 Introduction

The plant ionome referring to mineral nutrients and trace element composition contributes to both the growth and development and tolerance to abiotic and biotic stresses of plants.^[^ ^1^ ^]^ Essential micronutrient levels and bioavailability in crops not only affect the quality and quantity of crop production, but also via their diet have a profound impact on humans.^[^ ^2^ ^]^ Among nutrient elements, the essential trace elements such as zinc (Zn) and iron (Fe), play crucial roles in human health, especially with regard to their immune system and longevity. Zinc deficiency can, furthermore, lead to a decrease in taste perception, resulting in loss of appetite, unusual food cravings, and even stunted growth.^[^ ^3^ ^]^ Iron deficiency severely affects cognitive development and the immune systems of young children, and results in anemia in pregnant and lactating women.^[^ ^4^, ^5^ ^]^ Biofortification through genetic modification and crop improvement appears to be an important and economically sustainable approach.^[^ ^5^, ^6^, ^7^ ^]^ A better understanding of genes and gene networks controlling the uptake, translocation, and accumulation of mineral nutrients in crops holds promise for future resource‐efficient, sustainable crop production, and grain‐based health benefits. Whilst the genetic architecture analyses of the ionome have been dissected in many plants including *Arabidopsis thaliana*,^[^ ^8^, ^9^ ^]^ rice,^[^ ^10^, ^11^ ^]^ wheat,^[^ ^12^ ^]^ maize,^[^ ^13^ ^]^ bean,^[^ ^14^ ^]^ sorghum,^[^ ^15^ ^]^ rapeseed,^[^ ^16^ ^]^ this is not yet the case in buckwheat.

Buckwheat is an important cereal crop belonging to the *Fagopyrum* genus of the Polygonaceae, being widely cultivated in arid, semi‐arid, and high‐altitude regions of the Northern Hemisphere over more than 4000 years.^[^ ^17^ ^]^ Buckwheat has two highly cultivated species: Tartary buckwheat (*F. tataricum*) and common buckwheat (*F. esculentum*).^[^ ^18^, ^19^ ^]^ Tartary buckwheat is often used to study the genetic architecture and gene function owing to its properties of self‐pollination as opposed to the self‐incompatible common buckwheat.^[^ ^20^ ^]^ Tartary buckwheat originates in the Himalayan region, has strong ecological adaptability and abundant nutrients and rich bioactive flavonoids, and is the main grain crop in the high‐altitude areas of southwest China and an important autumn cash crop in southern China.^[^ ^21^ ^]^ Tartary buckwheat possesses abundant mineral nutrients and trace elements, and exhibits high tolerance to harsh, low‐nutrient, environments.^[^ ^22^, ^23^ ^]^ However, the genetic basis of the ionome in germplasm resources remains unclear to date, which limits the molecular breeding process of Tartary buckwheat.

The ionome of the plant is influenced by a summation of multiple internal and external factors including genomic features and climatic conditions as well as soil properties.^[^ ^1^, ^24^ ^]^ Here, the substantial differences in the elemental compositions of Tartary buckwheat grains harvested from different fields between 2020 to 2022 may be due to soil properties, germplasm characteristics and genetic diversity. By integrating genome‐wide association study and high‐throughput inductively coupled plasma mass spectrometry (ICP‐MS), numerous significant key loci and multiple potentially causal candidate genes associated with 20 ionomic traits were identified with a panel of 199 diverse Tartary buckwheat accessions. Three of these were further validated by biochemistry and molecular biology methods. This study thereby provides crucial insights into the genetic basis of natural ionomic variations in Tartary buckwheat.

## 2 Results

### 2.1 Ionome Variation among Tartary Buckwheat Grains

To investigate the natural variations in the grain ionome in Tartary buckwheat, the population of 199 Tartary buckwheat accessions based on large variations in the geographical origins, agricultural traits and genetic diversity was used in this study (Table S1, Supporting Information). As described in our previous works,^[^ ^20^, ^25^ ^]^ these accessions were determined by population structure analysis to include 13 HW (Himalayan wild accessions), 80 SL (Southwestern landraces), and 106 NL (Northern landraces). Here, we detected the variation of 20 elements in the grains of the accessions grown in two locations, namely Liangshan and Danzhou, China, in three consecutive years (Tables S2–S4, Supporting Information).

**Figure 1:**
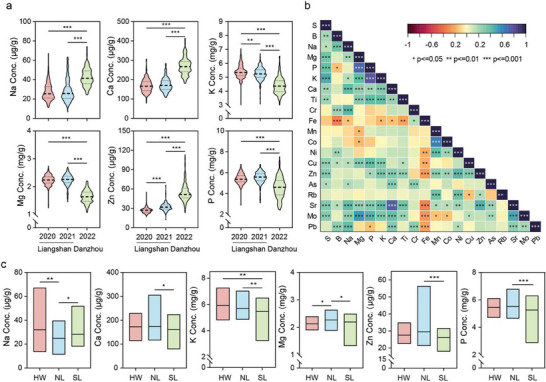
The ionome analysis based a natural population of Tartary buckwheat. a) Violin plots of Na, Ca, K, Mg, Zn, and P elemental concentrations of Tartary buckwheat seeds among different years and locations. Asterisk (**) and (***) indicate significant differences at *P* < 0.01 and *P* < 0.001 using one‐way ANOVA. b) Pearson's correlation coefficient among different elemental concentrations (The average of elemental concentrations in three years). c) Box plots of Na, Ca, K, Mg, Zn, and P elemental concentrations of Tartary buckwheat seeds in three populations. Asterisk (*), (**), and (***) indicate significant difference at *P* < 0.05, *P* < 0.01, and *P* < 0.001 using one‐way ANOVA.

Soil properties play a crucial role in the cultivation and growth of crops, such as pH value, greatly affecting the absorption of nutrient elements by crops.^[^ ^26^ ^]^ The soil is alkaline (pH > 7) in most regions of northern China, while soil in southern China is acidic (pH < 7) (Figure S1a, Supporting Information), which determines the differences in soil structure and properties between northern and southern China.^[^ ^27^ ^]^ The Na content in Tartary buckwheat grains grown in Danzhou with higher exchangeable Na^+^ was significantly larger than those grown in Liangshan with lower exchangeable Na^+^, and the same was true for Mg and P (**Figure** **1**a; Figure S1b,e,f, Supporting Information), indicating that these three elemental concentrations in soil had a great impact on the accumulation of Tartary buckwheat. By contrast, the levels of Ca and K in grains had an adverse trend to those of the soil (Figure 1a; Figure S1c,d, Supporting Information). The grain content of ten elements (K, Zn, As, Mo, Cu, S, Fe, Pb, Sr, Cr) showed significant differences across the three years, while eight ion compositions (Na, Ca, Mg, P, Co, Ni, Mn, Rb) varied in grains grown in Liangshan in comparison to those grown in Danzhou, but were invariant between harvest at the same location (Figure 1a; Figure S1, Supporting Information). The coefficients of variation (CVs) of essential macronutrients (Mg, P, S, K, Ca) ranged from 7.5% to 21.5% being considerable smaller than those of micronutrients (B, Mn, Fe, Cu, Zn, Mo), which ranged from 15.9% to 67.4%, and toxic elements (As and Pb), which ranged from 54.3% to 304.5% (Table S5, Supporting Information). Correlation analysis revealed that the five essential macronutrients (Mg, P, S, K, Ca) exhibited a significant positive correlation with one another, while Fe displayed a negative correlation with the six elements Ni, Cu, Zn, Sr, Mo and Pb (Figure 1b; and Table S6, Supporting Information).

The ionome profiles are detailly investigated based on the three population structures.^[^ ^20^, ^21^ ^]^ There were four differentially abundant ionomic elements, i.e., Na, Mg, B, and Mo in the NL cultivated group and five, i.e., K, Ni, B, Fe, S in the SL cultivated group compared to the HW wild group. Among them, the content of B in HW was higher than in both NL and SL (Figure 1c; Figure S3, Supporting Information). Thirteen elemental levels namely those of Na, Ca, K, Mg, Zn, P, Ni, Mo, Mn, Fe, Ti, Sr, S displayed significant differences between the NL and SL groups (Figure 1c; Figure S3, Supporting Information), suggesting ionome alteration in NL and SL was probably owing to natural or artificial selection during Tartary buckwheat domestication.

### 2.2 Genetic Basis of Ionome in Tartary Buckwheat Grains

**Figure 2:**
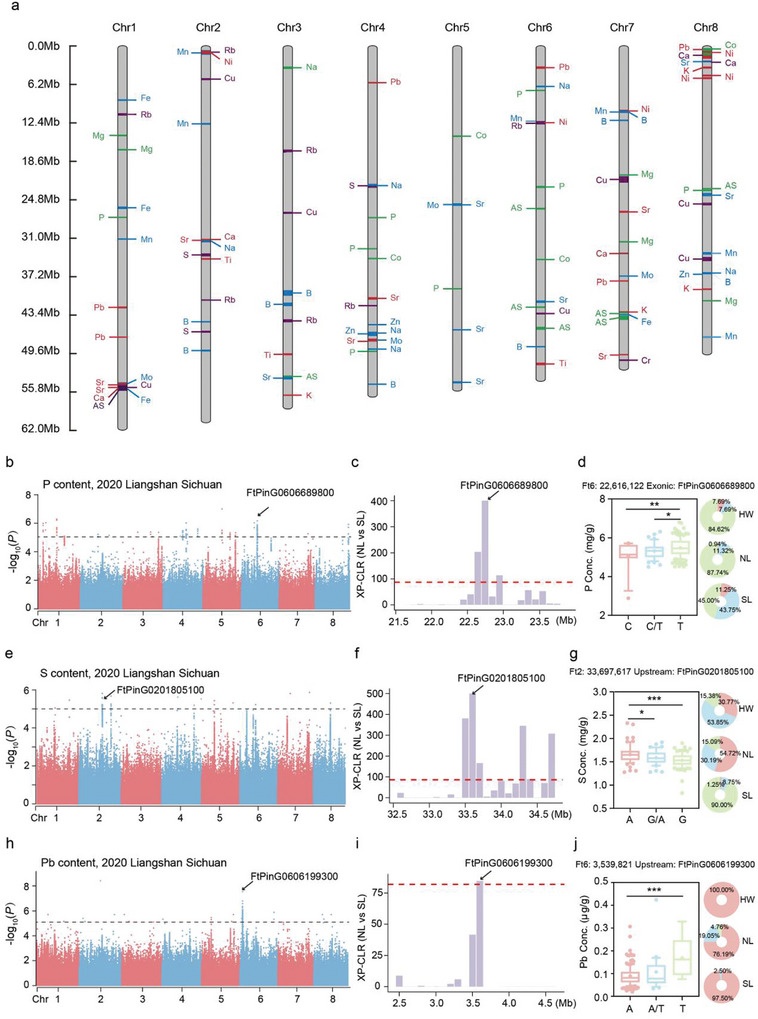
Information on the QTLs identified by GWAS. a) Distribution of 122 QTLs located on 12 chromosomes according to physical distance. The number on left column represents the physical location (Mb) of chromosomes and the letters marked on column represent the corresponding elements. b) Manhattan plot for P element. The black arrow indicates the location of *FtPinG0606689800* on Chr 6. The dashed line indicates the threshold (−log_10_ *P* = 5). c) Selective sweeps between SL and NL on Chr 6 using XP‐CLR. The red dashed horizontal lines indicate top 10% threshold. The black arrow indicates the position of *FtPinG0606689800*. d) Box plot for P concentration at Ft6: 22616122 (SNP) and frequencies of the haplotype in three groups. Data is presented as mean ± SD. Significant differences are indicated by asterisks: * *P* < 0.05, ** *P* < 0.01, One‐way ANOVA. e) Manhattan plot for S element. The black arrow indicates the location of *FtPinG0201805100* on Chr 2.The dashed line indicates the threshold (−log_10_ *P* = 5). f) Selective sweeps between SL and NL on Chr 2. The red dashed horizontal lines indicate top 10% threshold. The black arrow indicates the position of *FtPinG0201805100*. g) Box plot for S concentration at Ft2: 33697617 (SNP) and frequencies of the haplotype in three groups. Data is presented as mean ± SD. Significant differences are indicated by asterisks: * *P* < 0.05, *** *P* < 0.001, One‐way ANOVA. h) Manhattan plot for Pb element. The black arrow indicates the location of *FtPinG0606199300* on Chr 6. The dashed line indicates the threshold (−log_10_ *P* = 5). i) Selective sweeps between SL and NL on Chr 6 using XP‐CLR. The red dashed horizontal lines indicate top 10% threshold. The black arrow indicates the position of *FtPinG0606199300* in the selective sweep. j) Box plot for Pb concentration at Ft6: 3539821 (SNP) and frequencies of the haplotype in three groups. Data is presented as mean ± SD. Significant differences are indicated by asterisks: *** *P* < 0.001, One‐way ANOVA.

In order to assess the loci and candidate genes associated with the content of the 20 ions measured here, we conducted a genome‐wide association study analysis with 30‐fold deep whole‐genome resequencing data combining the 1354548 SNPs and phenotypic profiles derived from the three independent field conditions.^[^ ^28^ ^]^ A total of 122 loci containing 2606 candidate genes were identified in the study, including 46 loci in 2020 in Liangshan, 51 loci in 2021 in Liangshan, 25 loci in 2022 in Danzhou (**Figure** **2**a; Figure S4 and Table S7, Supporting Information). Among them, the ten elements S, Fe, K, Mo, Ti, Pb, Ca, Mg, Co, and Zn displayed from three to five GWAS loci, the seven ions Ni, Cu, Mn, Rb, As, B, and P were controlled by six to nine significant associations and the two elements Na and Sr were each associated with more than ten loci. 16 genomic loci were associated with at least two types of ion, including the 1.36 Mb region (Ft1: 54349299 – 55710904) on chromosome 1 which was associated to variation in the levels of As, Ca, Cu, Fe, Mo, Pb, and Sr elements. Similarly, five loci were repeatedly identified in GWAS for the five elements Ca, P, Sr, Na, Zn measured at least two different times or locations (Figure S4 and Table S7, Supporting Information). The locus 36546167 – 36818505 on chromosome 8 was simultaneously detected for Na and Zn contents, and homologs of the gene *FtPinG0808772500* located in this region, encoding protein phosphatase 2C (*PP2C*), which acts as a negative regulator by interacting with salt overly sensitive (SOS1) and inhibiting its Na^+^/H^+^ antiporter activity, resulting in salt sensitivity of *Arabidopsis thaliana* (Figures S7 and S20 and Table S7, Supporting Information).^[^ ^29^ ^]^ The lead SNPs 25731111 and 25630500 on chromosome 5 were consistently correlated with Mo and Sr contents in Liangshan in 2020, and the lead SNP 22931829 on chromosome 4 was connected to Na and S contents (Figures S4 and S7 and Table S7, Supporting Information), implying multiple ionomic traits might be controlled by the same or linked genetic loci. The GWAS signals for Mn content, a gene *FtPinG0606447600* on Chr 6 belonging to ATP‐binding cassette (*ABC*) transporter B family member as the candidate gene was identified (Table S7, Supporting Information). Several ABC transporters responded to Mn treatment and contributed to cellular homeostasis of manganese and iron ions in plants.^[^ ^30^ ^]^ The genes encoding heavy metal‐associated isoprenylated plant protein (HIPP) were located in the GWAS regions for As, Co, Rb, B, Na contents (Table S7, Supporting Information). Heavy metal‐associated (HMA) gene family in Tartary buckwheat had been reported to related with cadmium stress.^[^ ^31^ ^]^ The identification of these homologous genes suggests that our GWAS is reliable, and provides clues for exploration of genes responsible for ionomic variations in Tartary buckwheat germplasms.

The domestication and differentiation sweeps among three major populations have been clearly characterized in our previous studies.^[^ ^20^, ^21^ ^]^ By comparing GWAS signals with domestication and differentiation sweeps, we identified that 52 GWAS regions for ten elemental contents were located in selective sweeps, with 16 signals for seven elements in the HW versus NL comparison, 25 signals for six elements in the HW versus SL comparison, and 11 signals for six elements in the SL versus NL comparison (Table S8, Supporting Information). Among them, the GWAS signal for P content on Chr 6 simultaneously overlapped with selective sweeps of HW versus SL and SL versus NL comparisons, which coincided with the trait levels across SL and NL populations (Figures 1c and 2b,c; Figure S5 and Table S8, Supporting Information). The haplotypes of the SNP 22616122 on Chr 6, located in the exon of *FtPinG0606689800*, was associated with P content. The allele T was associated with higher P content, compared with the alleles C and C/T (Figures 1c and 2d). The lead SNP 33697617 on Chr 2, located in the upstream of *FtPinG0201805100* encoding nitrate reductase and associated with S concentration, overlapped with selective sweeps identified in SL versus NL (Figure 2e,f; Figure S5 and Table S8, Supporting Information). Tartary buckwheat varieties harboring haplotype A exhibited higher S content than those harboring haplotype G/A and G, with haplotype A accounting for 54,72% of the accessions in NL, 30.77% of the accessions in HW and none of the accessions in SL, which was in agreement with the phenotypic variations among the three groups (Figure 2g; Figure S3, Supporting Information). The GWAS signal on Chr 6 for Pb content simultaneously overlapped with the domestication and differentiation sweeps identified in HW versus NL, SL versus NL comparisons (Figure 2h,i; Figure S5 and Table S8, Supporting Information). The proportion of allele T of the SNP 3539821 located upstream of *FtPinG0606199300* on Chr 6 for higher Pb content was considerably lower in the SL than the NL sub‐population (Figure 2j). We thus speculate that not only the agronomic traits and metabolite contents, but also the levels of some elements were subjected to domestication and differentiation in Tartary buckwheat.^[^ ^20^, ^25^ ^]^

### 2.3 Genomic Divergence between 20A1 and Pinku Tartary Buckwheats

**Figure 3:**
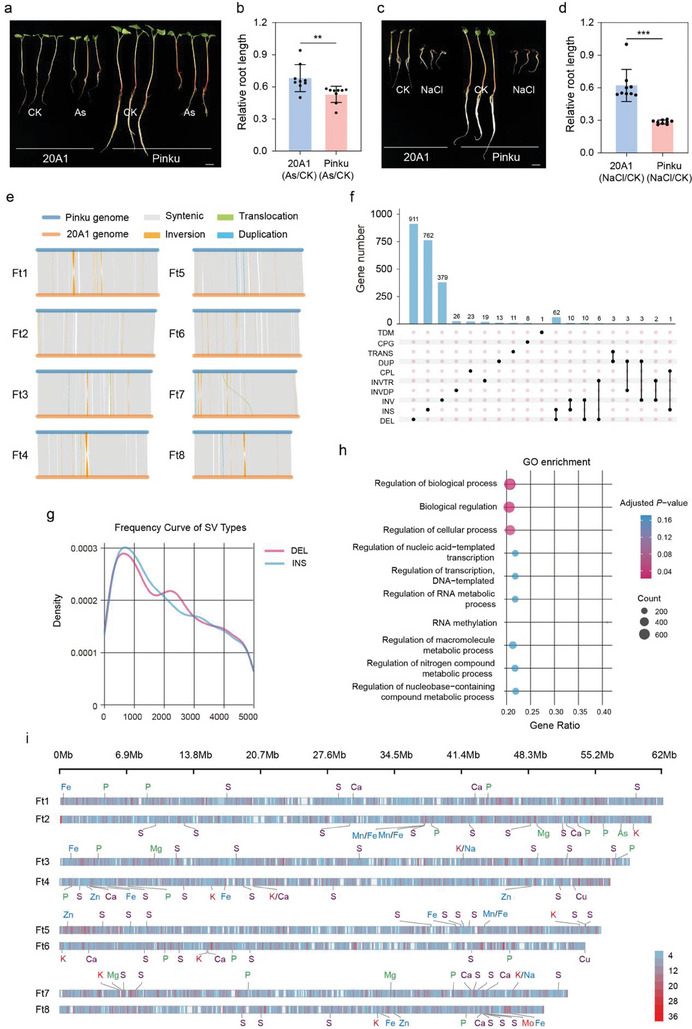
Comparative analyses of Pinku (cultivated) and 20A1 (wild) Tartary buckwheat genomes. a,b) The phenotype of 20A1 and Pinku after 0.2 µg ml^−1^ As treatment (a). Scale bar, 1 cm. Relative root length of 20A1 and Pinku under control and As treatment (b). *n* = 9. Asterisk (**) indicates significant difference at *P* < 0.01 using two‐tailed *t*‐test. c,d) The phenotype of 20A1 and Pinku after 150 mM NaCl treatment (c). Scale bar, 1 cm. Relative root length of 20A1 and Pinku under control and NaCl treatment (d). *n* = 9. Asterisk (***) indicates significant difference at *P* < 0.001 using two‐tailed *t*‐test. e) Syntenic analysis of the Pinku and 20A1 Tartary buckwheat genomes. f) The histogram illustrates the gene numbers of different types in structure variantions. Variation types: TDM, tandem repeat; CPG, copy gain in query; TRANS, translocated region; DUP, duplicated region; CPL, copy loss in query; INVTR, inverted translocated region; INVDP, inverted duplicated region; INV, inverted; INS, insertion in query; DEL, deletion in query. g) The density curve of deletion and insertion presenting in the promoter 5 kb of genes between Pinku and 20A1 genomes. h) GO enrichment of genes related to structural variations between the Pinku and 20A1 genomes. i) Distribution of SVs and the genes from GO enrichment related to the biological processes of 12 ionomic elements on 12 chromosomes according to physical distance. Red indicates high density and blue indicates low abundance SVs.

Given the discovery of ionome variations and domestication sweeps between wild and cultivated groups, we assume that genetic diversity directly influences phenotypic traits, as well as environmental factors. Additionally, the 20A1 representing wild type from Tibet exhibited stronger tolerance than the Pinku belonging to the cultivated population under salt or arsenic (As) stresses, respectively (A genomic and phenotypic database for buckwheat, BuckwheatGPDB) (**Figure** **3**a–d; Figure S6, Supporting Information). Therefore, we conducted a genome‐wide comparison between 20A1 and Pinku to dissect genetic divergence contributing to the phenotypic variations in the ionome. There was an overall collinear block of large segments between chromosomes, but some inversion, translocation and duplication could still be observed within the collinear blocks (Figure 3e; Table S9, Supporting Information). In order to detect whether the existence of structural variants (SVs) contribute to the significant ionomic differences between 20A1 and Pinku accessions, we continued to investigate the effects of SVs on annotated genes. A total of 2256 genes displayed structural variation (size ≥ 50 bp), and were defined as genes affected by SVs (Figure 3f; Table S10, Supporting Information). Among them, 911 genes displaying deletions, 762 genes displaying insertions and 379 genes with inverted regions, accounted for more than 90% of all SVs (Figure 3f). In addition, sixty‐two, ten and ten genes were co‐affected by deletion‐insertion, inverted region‐insertion, and inverted region‐deletion, respectively (Figure 3f), indicating that deletion, insertion and inverted region were the three most important structural variation types between the 20A1 and Pinku germplasms. The structural variations of non‐coding regions in upstream promoters of genes often lead to drastic changes in the transcript level of genes which subsequently results in a significant impact on phenotypic variations.^[^ ^32^ ^]^ We further investigated the SVs present in the upstream 5 kb core transcription initiation region of all genes, and found that the insertion and deletion types also accounted for the vast majority of SVs in these regions (Figure 3g). The distribution of the two types of SVs in the promoters of genes displayed a consistent trend, with the density of SVs reaching a peak at ≈800 bp upstream of the open reading frame where some key *cis*‐acting elements were located (Figure 3g; and Tables S11 and S12, Supporting Information). We thus inferred that the structural variations might determine the transcription of downstream genes by affecting the presence/absence of *cis*‐acting elements in the promoters.

We subsequently performed Gene Ontology (GO) enrichment analysis of these genes affected by upstream SVs with these mainly being enriched in biological regulation, macromolecule metabolic process and nitrogen compound metabolic process (Figure 3h; and Table S13, Supporting Information). Finally, we focused on the relationship between SVs and genes related to ion transport and homeostasis. The density map was used to show the distribution of SVs at the chromosomal level and labelled the sites of genes involved in the transport and homeostasis regulation processes of 14 ions namely P, Mg, Na, S, K, Ca, Mg, Fe, Cu, Zn, As, and Mo, and some of these genes were found to overlap with the high density SVs regions (Figure 3i; and Table S14, Supporting Information). Among them, homologs of a gene encoding a sodium hydrogen exchanger (FtNHX, *FtPinG0303339900*, *FtPinG0707983300*) were previously found to play a critical role in modulating salt homeostasis.^[^ ^33^ ^]^ Similarly, a homolog of a gene encoding an oligopeptide transporter (OPT3, *FtPinG0100013100*) mediating systemic iron and copper signaling in *Arabidopsis*,^[^ ^34^ ^]^ was identified in this analysis. These results illustrate that structural variations between 20A1 and Pinku may influence the absorption and transport processes of ions, or the crosstalk regulation of ions homeostasis in Tartary buckwheat.

### 2.4 GWAS for Na Levels and Identification of *FtACA13*

**Figure 4:**
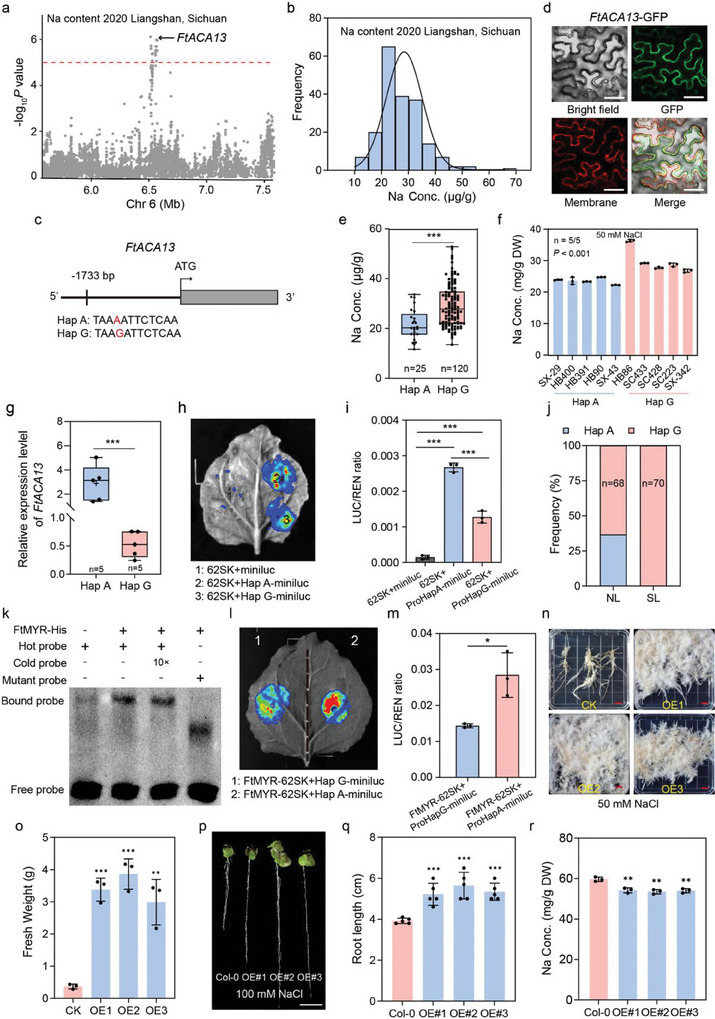
Identification of the *FtACA13* associated with Na content by GWAS. a) Local Manhattan plot of GWAS signals on Chr 6 for Na content. The dashed line represents the threshold (‐log_10_ *P* = 5). The black arrow indicates the position of *FtACA13*. b) Distribution of Na concentrations in 199 Tartary buckwheat accessions. c) Structure of *FtACA13* genomic sequence. A SNP in the promoter of *FtACA13* is marked with red letters and forms two haplotypes (Hap A and Hap G). d) Subcellular localization of FtACA13. Scale bar, 50 µm. e) Box plot showing Na content variation in two haplotypes. Asterisk (***)indicates significant difference at *P* < 0.001 using two‐tailed *t*‐test. f) The Na concentration of ten Tartary buckwheat accessions harboring Hap A and Hap G, respectively. *P* value was calculated using one‐way ANOVA. g) The expression level of *FtACA13* in accessions with the two haplotypes. The error bars indicate the mean ± SD. Asterisk (***) indicates significant difference at *P* < 0.001 using two‐tailed *t*‐test. h,i) Transcription activity of *FtACA13* promoters with two haplotypes. LUC signals of Hap A and Hap G promoters of *FtACA13*. 62SK, empty vector pGreen II 62‐SK; miniluc, empty vector pGreenII‐0800‐miniLUC; Hap A‐miniluc, the *FtACA13* promoter sequence harboring Hap A cloned into pGreenII‐0800‐miniLUC; Hap G‐miniluc, the *FtACA13* promoter sequence harboring Hap G cloned into pGreenII‐0800‐miniLUC (h). The LUC/REN ratio of signal regions in tobacco leaves (i). Data are shown as mean ± SD from three biological replicates. *** *P* < 0.001, as calculated using two‐tailed *t*‐test. j) Frequencies of the two haplotypes in the NL and SL groups. k) EMSA showing that FtMYR‐His could directly bind to the promoter of *FtACA13* comprising Hap G. Hot probe, the *FtACA13* promoter sequence comprising Hap G and labeled with biotin at 3′‐start; cold probe, the unlabelled sequence; mutant probe, the promoter sequence without Hap G. l,m) The LUC signals (l) and LUC/REN ratio (m) of *FtMYR* binding to the promoter of *FtACA13* comprising two haplotypes assayed by dual luciferase system in tobacco leaves. The error bars indicate the mean ± SD. Asterisk (*) indicate significant difference at *P* < 0.05 using two‐tailed *t*‐test. n,o) Phenotypes (n) and fresh weight (o) of overexpressing *FtACA13* hairy roots and wild type subjected to salt stress after three weeks. Scale bar, 1 cm. The error bars indicate the mean ± SD. Asterisk (***) and (**) indicate significant difference at *P* < 0.001 and *P* < 0.01 using two‐tailed *t*‐test. p–r) Phenotypes (p), root length (q) and Na content (r) of *Arabidopsis* lines heterologously expressing *FtACA13* and wild type subjected to salt stress after three weeks. Scale bar, 1 cm. The error bars indicate the mean ± SD. Asterisk (***) and (**) indicate significant difference at *P* < 0.001 and *P* < 0.01 using two‐tailed *t*‐test.

To explore low‐salt and salt‐tolerant genetic resources, we conducted GWAS analysis on the grains of the 199 Tartary buckwheat accessions displaying variation in Na content (**Figure** **4**a,b). The results indicated a significant locus on chromosome 6 was strongly associated with Na content across two harvests (Figure 4a; Figure S7, Supporting Information). A SNP located on the promoter of a gene‐encoding the plant auto‐inhibited Ca^2+^‐ATPase gene (*FtPinG0606295300*, *FtACA13*) was correlated with Na content, and could be divided into haplotypes A (Hap A) and G (Hap G) (Figure 4c; Figure S8, Supporting Information). Phylogenetic analysis revealed that *FtPinG0606295300* was the ortholog to *Arabidopsis thaliana AtACA13* (*AT3G22910*) (Figure S9, Supporting Information). Plasma membrane (PM)‐localized calcium ATPases pump calcium ions out of the cytosol and are involved in calcium ion transmembrane transport process.^[^ ^35^ ^]^ NaCl induces a rapid elevation of cytosolic Ca^2+^ levels in root cells. Furthermore, calcium ions (Ca^2+^) have been reported to promote salt tolerance through modulating the expression of many salt‐responsive genes.^[^ ^36^ ^]^ Subcellular localization revealed that the fluorescence signal of the FtACA13‐GFP fusion protein under the control of the 35S promoter was detected in the plasma membrane of *Nicotiana tabacum* leaves, which was confirmed by colocalization with the plasma membrane marker (Figure 4d). In addition, a Ca^2+^ flux assay showed that *FtACA13* overexpression hairy roots exhibited stronger Ca^2+^ flux than those of wild type (Figure S10, Supporting Information), further demonstrating that FtACA13 is a plasma membrane‐localized calcium ATPase.

Haplotype analysis revealed that Na accumulation in the grains of accessions harboring Hap A was significantly lower than that in those harboring Hap G in our collection under natural conditions (Figure 4e). Subsequently, the seedlings of five Tartary buckwheat accessions comprising Hap A accumulated low Na levels compared with those comprising Hap G under 50 mm NaCl treatment for three days (Figure 4f), suggesting the genetic variation in the promoter of *FtACA13* could determine Na accumulation in Tartary buckwheat and Hap A may be used as a molecular marker when screening for low‐Na germplasm resources. To investigate whether the variation in the promoter region affected the transcript level of *FtACA13*, we performed qRT‐PCR to measure the expression of *FtACA13* in the seedlings of five accessions of Hap A and Hap G groups under normal conditions. The Hap A group with lower Na concentration had a significant higher expression level of *FtACA13* than the Hap G group, consistent with the LUC expression driven by two haplotypes of *FtACA13* promoter in *Nicotiana tabacum* leaves (Figure 4g–i), further demonstrating that the promoter natural variation had a significant influence on *FtACA13* mRNA abundance. The frequency of Hap A was greater in the NL population than the SL population in our study (Figure 4j), implying that *FtACA13* had undergone selection during differentiation of the NL and SL groups. To detect potential upstream regulatory factors targeting *FtACA13* by binding *cis*‐acting element containing the haplotype sequences, we investigated the *cis* ‐regulatory elements of the promoter of *FtACA13* from the PlantCARE database and conducted a co‐expression clustering analysis of *FtACA13* through the transcriptome file of Tartary buckwheat seeds at 13 to 25 days after pollination (DAP) from previous study (Figure S11, Supporting Information).^[^ ^37^ ^]^ Intriguingly, consistent with the expression patterns of *FtACA13* during seed development, a Golden2‐like transcription factor FtMYR (*FtPinG0808584500*, G2‐like, MYB‐related protein) (Figure S12, Supporting Information), was predicted to bind to the *cis* ‐acting element (AGAAT) containing Hap G of the *FtACA13* promoter. By using the EMSA and dual‐luciferase assays, we then demonstrated that FtMYR could showed a higher binding ability to the promoter of *FtACA13* harboring Hap G than that harboring Hap A (Figure 4k–m). The yeast self‐activation experiment showed that FtACA13 had no transcriptional‐activation activity and might act as a transcriptional suppressor (Figure S11, Supporting Information). These results illustrate that FtACA13 is responsible for Na content and Hap A of *FtACA13* may be an elite haplotype resource for achieving low‐sodium grains of Tartary buckwheat.

Previous studies reported that overexpressed *ACA* family genes in plants could obviously promote salinity tolerance by enhancing the expression of stress‐responsive genes and decreasing membrane permeability.^[^ ^38^, ^39^ ^]^ To better understand the function of FtACA13, we next generated *FtACA13*‐overexpressing transgenic hairy roots in the background of the “Pinku” cultivar and obtained heterologous *FtACA13* overexpressing *Arabidopsis* (Figure S13, Supporting Information). The transgenic hairy roots overexpressing *FtACA13* (OE#1, OE#2, and OE#3) were more tolerant to salt stress and had better growth than the wild type after two weeks of treatment with 50 mm NaCl (Figure 4n,o). Simultaneously, the *FtACA13* overexpressing hairy roots exhibited no significant growth difference under control conditions, suggesting that overexpression of *FtACA13* in Tartary buckwheat can significantly enhance salt tolerance at least under the tested NaCl concentration (Figure S14, Supporting Information). *FtACA13* overexpression hairy roots showed a stronger Ca^2+^ flux with NaCl treatment, compared with the wild type, further demonstrating the function of FtACA13 in salt tolerance (Figure S10, Supporting Information). Previous study indicated that the abundant antioxidant metabolite procyanidin could improve salt tolerance in apple and tomato.^[^ ^40^ ^]^ The metabolites L‐phenylalanine, procyanidin, and isovitexin were significantly higher in *FtACA13* overexpressing hairy roots than in the wild type (Figure S15, Supporting Information), however, further research is needed to determine whether these changes are related to the salt tolerance of these lines. Compared to wild‐type seedlings, the seedlings of the three overexpressing *FtACA13 Arabidopsis* lines (OE#1, OE#2, and OE#3) accumulated less Na and had longer roots, when grown on medium containing 100 mM NaCl for three weeks, indicating that *FtACA13* overexpressing lines exhibited salt exclusion and tolerance in high‐salt environments (Figure 4p–r; Figures S16 and S17, Supporting Information). The protein structure of FtACA13 and molecular docking of a sodium ion to it were also predicted with high accuracy by AlphaFold 3 (Figures S18 and S19, Supporting Information).^[^ ^41^ ^]^ Taken together, our results indicate that *FtACA13* is responsible for the low‐salt grain and involved in salt tolerance of Tartary buckwheat. Additionally, a 144 bp insertion was located in the upstream 2756 bp of *FtACA13* in 20A1, and the expression level of *FtACA13* in 20A1 was higher than those in Pinku, which might lead to the significant phenotypic difference in salt tolerance between 20A1 and Pinku (Figure 3c; Figures S20 and S21, Supporting Information). *FtACA13* was also identified within the domestication sweeps in HW versus SL by XP‐CLR identified in previous studies (Figure S22, Supporting Information),^[^ ^20^, ^21^ ^]^ implying that *FtACA13* which is responsible for Na content might have undergone domestication on the transition from wild to cultivated Tartary buckwheat.

### 2.5 The Potential Contribution of *FtYPQ1* in Ensuring High Zn Levels in Tartary Buckwheat Grains

**Figure 5:**
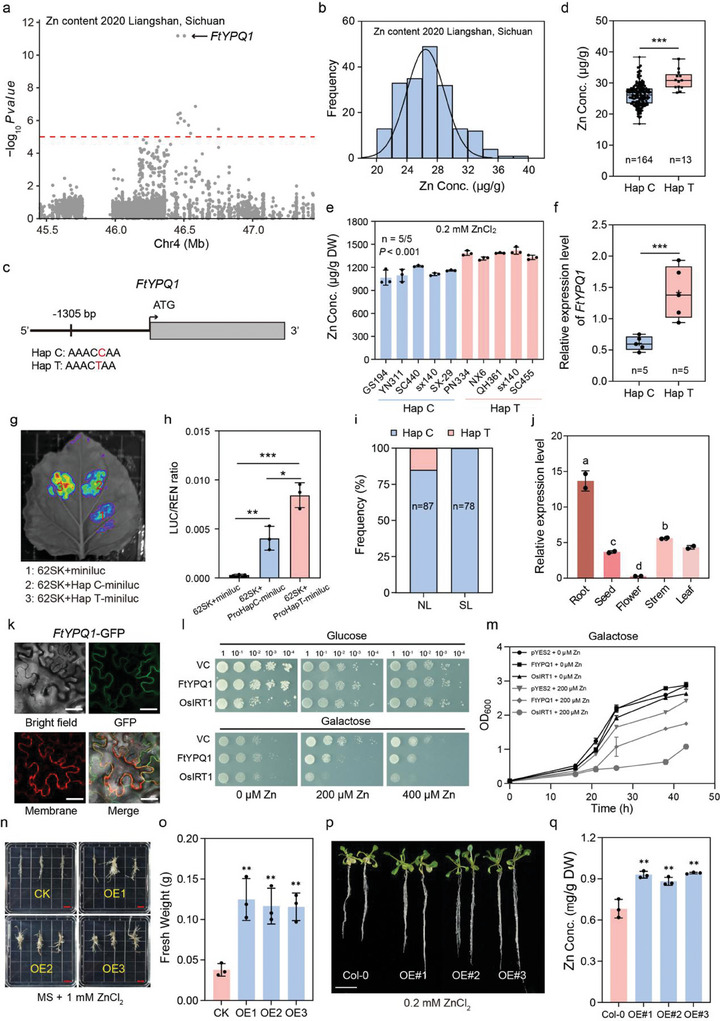
Characterization of the role of *FtYPQ1* in Zn accumulation by GWAS. a) Local Manhattan plot of GWAS signals on Chr 4 for Zn content. The dashed line represents the threshold (‐log_10_ *P* = 5). The black arrow indicates the position of *FtYPQ1*. b) Distribution of Zn concentration in 199 Tartary buckwheat accessions. c) Structure of *FtYPQ1* genomic sequence. A SNP in the promoter of *FtYPQ1* is marked with red letters and forms two haplotypes (Hap C and Hap T). d) Box plot showing Zn content variation in two haplotypes. The error bars indicate the mean ± SD. Asterisk (***) indicates significant difference at *P* < 0.001 using two‐tailed *t*‐test. e) The Zn concentration of ten Tartary buckwheat accessions harboring Hap C and Hap T with 0.2 mm ZnCl_2_ treatment, respectively. *P* value was calculated using one‐way ANOVA. f) The expression level of *FtYPQ1* in accessions with the two haplotypes. The error bars indicate the mean ± SD. Asterisk (***)indicates significant difference at *P* < 0.001 using two‐tailed *t*‐test. g,h) Transcription activity of *FtYPQ1* promoters with two haplotypes. LUC signals of Hap C and Hap T promoters of *FtYPQ1*. 62SK, empty vector pGreen II 62‐SK; miniluc, empty vector pGreenII‐0800‐miniLUC; Hap C‐miniluc, the *FtYPQ1* promoter sequence harboring Hap C cloned into pGreenII‐0800‐miniLUC; Hap T‐miniluc, the *FtYPQ1* promoter sequence harboring Hap T cloned into pGreenII‐0800‐miniLUC (g). The LUC/REN ratio of signal regions in tobacco leaves (h). Data are shown as mean ± SD from three biological replicates. * *P* < 0.05, ** *P* < 0.01, *** *P* < 0.001 as calculated using two‐tailed *t*‐test. i) Frequencies of the two haplotypes in the NL and SL groups. j) The tissue expression pattern of *FtYPQ1* in Tartary buckwheat. The error bars indicate the mean ± SD. The significant analysis by two‐tailed *t*‐test. k) Subcellular localization of *FtYPQ1*. Scale bar, 50 µm. l,m) Zinc transport activity of *FtYPQ1* in yeast. The Zn‐sensitive yeast mutant (*Δzrc1*) transformed with empty vector (EV), *FtYPQ1*, and positive control *OsIRT1* were serially diluted and grown on the media containing with or without ZnCl_2_ for 3 days (l). Yeast growth curve experiment. Data are presented as means ± SD with three biological replicates (m). n,o) Phenotypes (n) and fresh weight (o) of overexpressing *FtYPQ1* hairy roots and wild type subjected to zinc stress after three weeks. Scale bar, 1 cm. The error bars indicate the mean ± SD. Asterisk (**) indicates significant difference at *P* < 0.01 using two‐tailed *t*‐test. p,q) Phenotype (p) and Zn content (q) of *Arabidopsis* lines heterologously expressing *FtYPQ1* and wild type subjected to 0.2 mm ZnCl_2_ stress after three weeks. Scale bar, 1 cm. The error bars indicate the mean ± SD. Asterisk (**) indicates significant difference at *P* < 0.01 using two‐tailed *t*‐test.

Genetic biofortification approaches and identification of the genetic determinants of variation would be efficient modern breeding practices for Tartary buckwheat with high Zn content.^[^ ^42^ ^]^ Through GWAS on two independent harvests, a significant locus on chromosome 4 was consistently identified related to Zn levels in grain (**Figure** **5**a,b; Figure S23, Supporting Information). Gene annotation revealed that the most significant SNP at F4: 46447322 in this region was located in the promoter of a gene encoding a vacuolar amino acid transporter (*FtYPQ1*, *FtPinG0404619100*) (Figure 5c; Figure S24, Supporting Information). Moreover, the SNP yielded two haplotypes, Hap T correlating with higher Zn content and Hap C correlating with lower Zn content in the natural‐variation population (Figure 5d; Figure S25, Supporting Information). We randomly selected five accessions harboring both haplotypes in order to investigate the Zn level phenotypes following 0.2 mm ZnCl_2_ treatment for three days. The seedlings of the five accessions containing Hap T accumulated higher levels of Zn compared with those containing Hap C, demonstrating the haplotype was closely associated with Zn content in Tartary buckwheat seeds (Figure 5e). The expression analysis indicated that the transcript level of *FtYPQ1* in accessions harboring Hap T was higher than those harboring Hap C (Figure 5f). In addition, transient activation assays demonstrated that the promoter of Hap T possessed higher LUC activity compared to that of Hap C, further supporting the natural variation in the promoter of *FtYPQ1* had a significant impact on its expression and function (Figure 5g,h). We further analyzed the frequency of the haplotypes in Tartary buckwheat accessions and found that the Hap T allele was present in the NL group, but not in the SL group (Figure 5i), which is in agreement with the Zn phenotypes exhibited by the NL and SL groups (Figure 1c), implying that *FtYPQ1* might have undergone selection during Tartary buckwheat breeding. We therefore infer that *FtYPQ1* may regulate Zn content in Tartary buckwheat.

*FtYPQ1* exhibited a high expression level in roots of Tartary buckwheat from the transcriptome file of BuckwheatGPDB (Figure 5j), thus we speculated that *FtYPQ1* might play a role in the uptake of zinc ions from soil and translocation from roots to shoots in Tartary buckwheat seedlings. The subcellular localization of the gene product revealed that FtYPQ1 located to the plasma membrane, suggesting that it might function as a transport protein (Figure 5k). To evaluate whether FtYPQ1 possessed transport activity for zinc, we conducted a test by heterologously expressing *FtYPQ1* in the Zn‐sensitive yeast mutant (*Δzrc1*). *OsIRT1* was used as a positive control.^[^ ^43^ ^]^ Under 200–400 µm ZnCl_2_ addition, the growth of yeast cells expressing *FtYPQ1* exhibited more severe inhibition than those carrying empty vector pYES2 even though it was better than those expressing positive control *OsIRT1*, consistent with the results of the growth curve (Figure 5l,m), indicating that FtYPQ1 exhibited Zn transport activity. We thus propose that the FtYPQ1 protein might interact with zinc ions via TRP‐56 and CYS‐367 (Figures S26 and S27, Supporting Information).^[^ ^41^ ^]^ To investigate the function of *FtYPQ1* in vivo, we overexpressed *FtYPQ1* in Tartary buckwheat hairy roots and treated hairy roots of different genotypes with 0.2 mm ZnCl_2_ (Figure S28, Supporting Information). Compared with the wild‐type, *FtYPQ1* overexpressing lines displayed a better growth under excess zinc stress, but no significant phenotypic difference among various hairy roots grown on MS medium, indicating that *FtYPQ*1 did indeed confer zinc tolerance to hairy roots (Figure 5n,o; Figure S29, Supporting Information).The NMT assay showed that *FtYPQ1* overexpression hairy roots exhibited higher Zn^2+^ flux than those of wild type under zinc stress, further demonstrating the function of FtYPQ1 for transport zinc (Figure S30, Supporting Information). Moreover, we heterogeneously overexpressed *FtYPQ1* in *Arabidopsis* (Figure S31, Supporting Information). The three *FtYPQ1* overexpressing *Arabidopsis* lines displayed higher Zn content and better growth than the wild‐type seedlings under 0.2 mm ZnCl_2_ treatment for three weeks, and these different genotypic materials grown in MS without any treatment exhibited no phenotypic difference (Figure 5p,q; Figure S32, Supporting Information), suggesting *FtYPQ1* overexpression lines could accumulate more Zn and were hence more tolerant to zinc stress. As is well known, the content of flavonoids in Tartary buckwheat is much high than in common buckwheat.^[^ ^44^ ^]^ In addition, flavonoids might affect zinc homeostasis, uptake, and transport, and the interaction of flavonoids with zinc might change the antioxidant properties and some biological effects of the flavonoids.^[^ ^45^ ^]^ We thus further investigated whether overexpressing *FtYPQ1* could change the content of flavonoids in hairy roots. The results indicated that the contents of the flavonoids apigenin‐8‐C‐glucoside and quercitrin in the *FtYPQ1* overexpressing hairy roots were higher than those in the wild type, implying that *FtYPQ1* might have an indirect impact on flavonoids content via the promotion of the accumulation of zinc (Figure S33, Supporting Information). By comparing the genomes of Tartary buckwheat (Pinku) and golden buckwheat (*Fagopyrum dibotrys*, Luoji Mountain), being a wild relative of Pinku,^[^ ^46^, ^47^ ^]^ we identified a long terminal repeat retrotransposons (LTR‐RTs) existing in the promoter of *FtYPQ1* in Pinku, which was not present in *FdYPQ1* in *F. dibotrys* (Figure S34, Supporting Information). Subsequently, expression analysis revealed that *FdYPQ1* displayed a higher transcript level in *F. dibotrys* than in Pinku (Figure S35, Supporting Information). We speculated that the LTR‐RTs might affect the function of FtYPQ1 between *F. dibotrys* and *F. tataricum*.

### 2.6 *FtNHX2* Positively Regulating as Content Identified by GWAS

**Figure 6:**
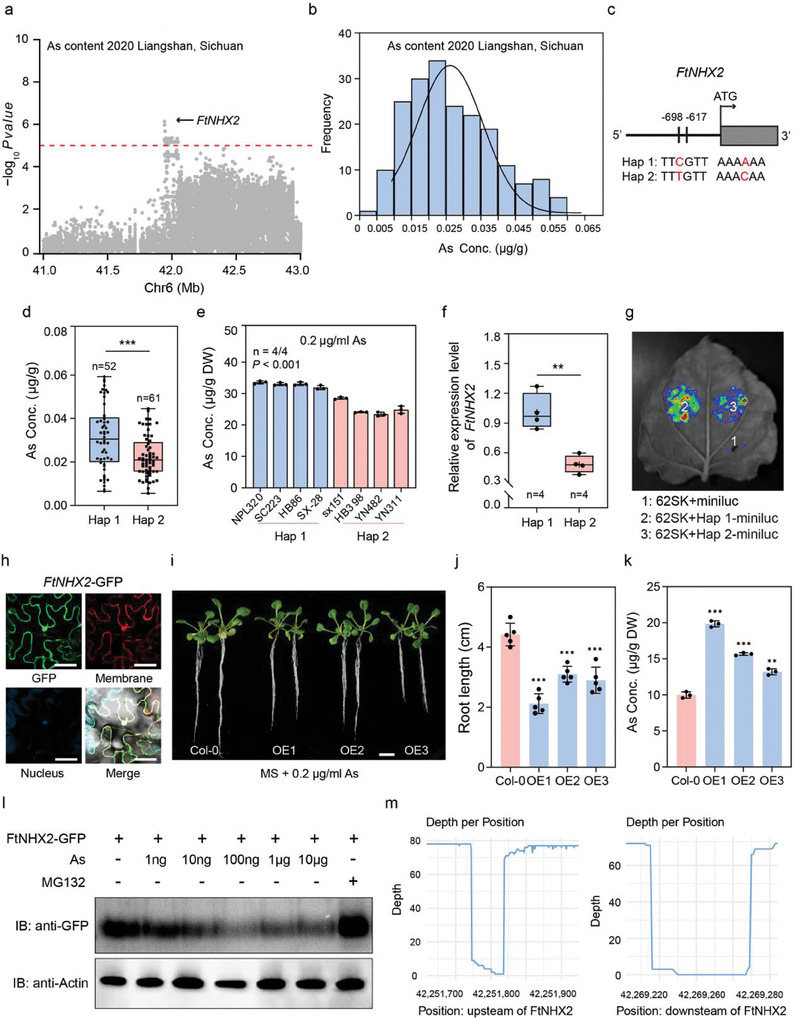
*FtNHX2* by GWAS, positively regulates As content. a) Local Manhattan plot of GWAS signals on Chr 6 for arsenic content. The dashed line represents the threshold (‐log_10_ *P* = 5). The black arrow indicates the position of *FtNHX2*. b) Distribution of As concentration in 199 Tartary buckwheat accessions. c) Structure of *FtNHX2* genomic sequence. The SNPs in the promoter of *FtNHX2* is marked with red letters and forms two haplotypes (Hap 1 and Hap 2). d) Box plots showing As content variation in two haplotypes. The error bars indicate the mean ± SD. Asterisk (***) indicates significant difference at *P* < 0.001 using two‐tailed *t*‐test. e) The As concentrations of eight Tartary buckwheat accessions harboring Hap 1 and Hap 2 with 0.2 µg ml^−1^ As treatment, respectively. *P* value was calculated using one‐way ANOVA. f) The expression level of *FtNHX2* in accessions with the two haplotypes. The error bars indicate the mean ± SD. Asterisk (**) indicates significant difference at *P* < 0.01 using two‐tailed *t*‐test. g) Transcription activity of *FtNHX2* promoters with two haplotypes. 62SK, empty vector pGreen II 62‐SK; miniluc, empty vector pGreenII‐0800‐miniLUC; Hap 1‐miniluc, the *FtNHX2* promoter sequence harboring Hap 1 cloned into pGreenII‐0800‐miniLUC; Hap 2‐miniluc, the *FtNHX2* promoter sequence harboring Hap 2 cloned into pGreenII‐0800‐miniLUC. h) Subcellular localization of *FtNHX2*. Scale bar, 50 µm. i–k) Phenotypes (i), root length (j) and As content (k) of overexpressing *FtNHX2 Arabidopsis* lines and wild type subjected to 0.2 µg ml^−1^ arsenic stress after three weeks. Scale bars, 1 cm. Data are presented as means ± SD. Asterisk (***) and (**) indicates significant difference at *P* < 0.001 and *P* < 0.01 using two‐tailed *t*‐test. l) Protein degradation assay of FtNHX2‐GFP. The same amount of purified FtNHX2‐GFP proteins was incubated with different amounts As. FtNHX2‐GFP proteins were detected by immunoblotting with antibodies against GFP. m) The deep sequencing of SVs located in the upstream and downstream of *FtNHX2*.

Cultivating and planting low‐arsenic Tartary buckwheat is a fundamental method to prevent arsenic from entering the food chain, avoid arsenic toxicity, and ensure food safety. To explore germplasm resources with low‐arsenic content in Tartary buckwheat and key genes regulating arsenic content, we performed GWAS on grain As levels identifying a significant signal locus on chromosome 6 associated with As levels in Tartary buckwheat grains (**Figure** **6**a,b; Figure S36, Supporting Information). Two SNPs in the GWAS region were located in the promoter of a gene encoding Na^+^/H^+^ exchanger (*FtPinG0606926200*, named as *FtNHX2*), and these defined two haplotypes (Hap 1 and Hap 2) associated with the As levels (Figure 6c; Figure S37, Supporting Information). Grain As levels were significantly lower in Hap 2 than in Hap 1 genotypes, consistent with the results of As content in seedlings comprising different haplotypes following three days of 0.2 µg ml^−1^ As treatment (Figure 6d,e). The mRNA abundances of *FtNHX2* in the accessions harboring Hap 1 displayed a stronger LUC signal than those harboring Hap 2, illustrating that natural variations in the promoter region affected the expression level of *FtNHX2* and that *FtNHX2* might play a pivotal role in regulating As content of Tartary buckwheat (Figure 6f,g).

Subcellular localization assay revealed that the FtNHX2 protein was widely distributed between the cell nucleus and membrane of *N. benthamiana* leaves (Figure 6h). To detect the function of FtNHX2, its heterologous expression in *Arabidopsis* was carried out and the resultant transformants were subjected to arsenic tolerance assays (Figure 6i; Figure S38, Supporting Information). Transgenic plants exhibited growth inhibition of shorter roots and higher As accumulation compared to the wild type under As treatment, whereas the different genotypes grown on MS medium did not exhibit obvious phenotype difference (Figure 6i–k; Figure S39, Supporting Information). This suggests that FtNHX2 may act as a regulator to regulate As tolerance. Previous study has unveiled that arsenic trioxide could increase the expression and activity of Na^+^/H^+^ exchanger isoform 1 (*NHE1*) resulting in intracellular alkalization and higher Madin‐Derby canine kidney (MDCK) cell proliferation.^[^ ^48^ ^]^ Subsequently, we performed a protein degradation assay to detect the effect of As on FtNHX2 protein. The FtNHX2 protein fused with a GFP tag exhibited a greater rate degradation with the increase of As concentration, while the addition of MG132, an proteasome inhibitor, could prevent its degradation, indicating that the FtNHX2 protein might be degraded through the 26s proteasome pathway (Figure 6l). Given the structural variants identified between Pinku and 20A1 genomes, we found that 54 and 46 bp deletions existed in the upstream and downstream of *FtNHX2* in 20A1. *FtNHX2* had a lower expression level in 20A1, compared to Pinku, which might be the cause of phenotypic variance between 20A1 and Pinku under arsenic stress (Figure 6m; Figure S40, Supporting Information).

## 3 Discussion

Plants accumulate a wide spectrum of essential mineral nutrients and several beneficial elements to meet general plant growth and environmental adaptation, as well as act as indispensable resources of energy, nutrition to humans.^[^ ^1^, ^2^, ^3^, ^4^, ^5^ ^]^ The large‐scale ionome atlas have been extensively investigated and constructed in crops,^[^ ^8^, ^9^, ^10^, ^11^, ^12^, ^13^, ^14^, ^15^, ^16^ ^]^ implying the importance of theoretical research and practical breeding applications in botany or agricultural production. Tartary buckwheat is known as the “Oriental God grass” because of its rich micronutrients and bioactive ingredients.^[^ ^22^, ^23^ ^]^ The genome and metabolomes profilings of Tartary buckwheat have been clearly characterized and established in our previous studies.^[^ ^20^, ^21^, ^25^ ^]^ However, the ionomic study and genetic determinants of ionomic variation in Tartary buckwheat remain elusive. Our study has investigated 20 ion elements among 199 Tartary buckwheat accession grains, including thirteen elements might undergoing selection during domestication of Tartary buckwheat. The levels of non‐essential toxic elements exhibit larger variations than macronutrients and micronutrients levels in Tartary buckwheat, which coincides with previous reports on rice and *Arabidopsis*.^[^ ^8^, ^9^, ^10^, ^11^ ^]^ The results further demonstrate that the essential nutritional elements in plant are more tightly regulated by precise biological processes to maintain relatively stable level for growth and development. By integrating bioinformatic and genetic tools to identify the genes and gene networks that directly controlling the ionome is an efficient and low‐cost analysis method.^[^ ^1^, ^49^ ^]^ 122 loci containing 2606 candidate genes associated with 20 ion elements are identified in Tartary buckwheat grains by performing GWAS. Unfortunately, we cannot thoroughly verify the corresponding functions of all the potential candidate genes, but only focus on some important traits and genes related to ion transport, and thus, some key signals and genes need further verification to dissect the relationship and molecular mechanism in future study. These results collectively provide not only a large‐scale atlas of the Tartary buckwheat ionome, but also valuable genomic loci and gene resources for precision genetic improvement of Tartary buckwheat.

Previously, salt stress alters Ca^2+^ levels in cellular and Ca^2+^‐ATPase family proteins could enhance salt tolerance in plants due to reduced accumulation of reactive oxygen species, while direct evidence for the role of Ca^2+^‐ATPase pumps in salinity tolerance is lacking.^[^ ^36^, ^38^, ^39^ ^]^ Here, overexpression *FtACA13* hairy roots of Tartary buckwheat show a stronger Ca^2+^ flux than those of wild type with or without NaCl treatment, indicating FtACA13 functions as Ca^2+^‐ATPase pump in response to salt stress, which directly supports FtACA13's involvement in salt tolerance. Different genetic determinants drive the formation of different ecotypes in crop, and higher soil salinity in northern China compared to southern China because of significant differences in the topographic and precipitation factors between northern and southern China.^[^ ^50^ ^]^ We provide multiple evidences that the increased frequency of a haplotype A of *FtACA13* with high expression in NL population is responsible for the greater salt tolerance of Tartary buckwheat, implying that a molecular marker might be designed according to the excellent haplotype to screen salt‐tolerance germplasms. The *FtACA13* also might be applied in low‐salt Tartary buckwheat grains breeding, contributing to the shifts from managing saline‐alkaline land toward breeding salt‐tolerant crops in agriculture, which is in accordance with the modern concept of rapid and low‐cost breeding.^[^ ^51^, ^52^ ^]^ Previous study reports that the ectopic expression of *FtWRKY46* from Tartary buckwheat results in increased salt tolerance in transgenic *Arabidopsis*.^[^ ^53^ ^]^ A WRKY transcription factor (*FtWRKY49*, *FtPinG0201759100*) is also identified for Na content across two years in this study (Table S7, Supporting Information). *FtWRKY49* might regulate the expression of salt stress related genes to participate in salt tolerance, which needs to be proved by further study.

Zinc deficiency is a critical challenge for food crop production, resulting in decreased yields and nutritional quality. Hence, zinc contents are widely focused on and examined in various tissues of crops, especially in seed.^[^ ^8^, ^9^, ^10^, ^11^, ^12^, ^13^, ^14^, ^15^, ^16^ ^]^ Rice and wheat, as main food crops, are known as poor dietary sources of Zn due to the average grain content 20–35 mg kg^−1^ Zn for wheat and 25 mg kg^−1^ Zn for rice, but buckwheat possessing relatively high zinc content.^[^ ^3^, ^54^ ^]^ In support of this conclusion, the average zinc content of Tartary buckwheat gains in Danzhou is ≈54 mg kg^−1^ in our survey (Table S5, Supporting Information). The key dominant *FtYPQ1* gene has been proved to contribute for transport and accumulation of zinc by multiple experimental evidences, which might explain why Tartary buckwheat grains contain relatively high levels of zinc compared to other crops. The results go counter to these observations of Zinc‐Regulated Transporter (ZIP) and Metal Tolerance/Transport Protein (MTP) involvement in Zn uptake, transport, storage and detoxification.^[^ ^3^ ^]^ Moreover, the Hap T type in promoter of *FtYQP1* likewise provides assistance in the development of high‐zinc varieties. Our study can offer theoretical guidance for molecular breeding of high zinc Tartary buckwheat in the future. However, there are some challenges to achieve these goals, such as the current unavailability of genetic transformation systems in Tartary buckwheat. Although successful and stable genetically transformed system in Tartary buckwheat has been reported and utilized.^[^ ^55^ ^]^ It will need more time and patience to overcome this problem.

NHX2 of *Arabidopsis*, the homolog of FtNHX2, interaction or dissociation with calmodulin‐like protein CML18 as a pH‐sensing protein is involved in the cellular K^+^‐ and pH‐stats.^[^ ^56^ ^]^ While this study finds that heterogeneously overexpressed *FtNHX2* in *Arabidopsis* exhibits reduced arsenic tolerance compared to the wild type. We speculate that this might be due to the FtNHX2 protein degradation through the 26s proteasome pathway with excess arsenic. These findings enrich the functional research of *NHX* family genes in plants, as a large number of NHX proteins responsible for potassium and sodium transport.^[^ ^33^ ^]^

In summary, the current study first investigates grain ionome among various Tartary buckwheat germplasms and performs comparative genomic analysis between “20A1” and “Pinku” accessions to explore the genetic difference determining phenotypic variation. Moreover, the identified candidate genes at the GWAS regions would provide a better understanding of the genetic bases underlying the ionomic variation, hold greater promise for biofortification and facilitate the evolution for Tartary buckwheat improvement.

## 4 Experimental Section

### Plant Materials and Growth Conditions

The detailed information ≈199 Tartary buckwheat accessions was listed in Table S1 (Supporting Information), including accession name, position of origin, subpopulation type, 30‐fold depth resequencing data and so on, is as described in previous studies.^[^ ^28^ ^]^ The seeds of 199 Tartary buckwheat varieties harvested in Zhaojue County (27°45′‐28°21′ N, 102°22′‐103°19′ E) of Sichuan province in 2020 and 2021 and Danzhou City (19°11′‐19°52′ N, 108°56′‐109°46′ E) of Hainan province in 2022 were used for ionomic profiling analysis, respectively. All accessions were sown in early April and harvested in July. Each accession was planted in each field in a randomized complete block design. Plants inside each row were used for sampling to avoid the edge effect. Tartary buckwheat lines planted in both places had no fertilization and pesticides throughout the growth period, only irrigation as needed. About 100 mature seeds from three plants per accession were collected and pooled for ionomic measurements.

The *Arabidopsis* Columbia ecotype (Col‐0) for transgenic experiments and *Nicotiana benthamiana* seedlings for molecular biology experiments were used in this study. Plants were cultivated in soil in an artificial climate chamber under a 16 h light/8 h dark photoperiod at a temperature regime of 22 °C for the photophase and 16 °C for the scotophase.

### Ionomic Elements Analysis

The materials were dried at 80 °C for 5 d and smashed and filtered through a 40‐mesh sieve. 0.2000 g (accurate to 0.0001 g) of the sample was dissolved in 1 mL distilled water and 4 mL HNO_3_‐H_2_O_2_ in 15 ml digestion tube according to previous studies.^[^ ^57^ ^]^ After standing for a moment, the digestion tube was put into an inner bucket for digestion in a super microwave digestion system (EXPEC 790S, China). After digestion, the sample was transferred to a 50 mL plastic volumetric flask diluting to the mark, and the ion concentrations were measured by inductively coupled plasma mass spectrometry (SUPEC 7000, Puyu technology, Hangzhou, China) with parameters RF power 1450 W, atomizing gas flow 1.10 L min^−1^, auxiliary gas flow 1.0 L min^−1^, cooling gas flow 14.0 L min^−1^, collision gas flow 1.40 mL min^−1^, flush/analysis pump speed 30 rpm, sampling depth 3.16 mm.

### Sequencing Data and Genome‐Wide Association Study (GWAS)

Based on the resequencing data of 199 Tartary buckwheat accessions in previous study,^[^ ^28^ ^]^ the genome‐wide association analysis was performed on the compositions of 20 ionomic elements in independent environments. A total of 1354548 filtered SNPs were identified for GWAS analysis using factored spectrally transformed linear mixed models (FaST‐LMM).^[^ ^58^ ^]^ The effective SNPs were tested by GEC v0.2 program, and the significance threshold were estimated with *P* = 1×10^−5^. According to the peak SNPs associated with ion contents, the 100 kb flanking regions were scanned to identify candidate genes. Subsequently, the haplotype of target genes was analyzed by Candihap V1.3.0 software with default parameters.

### Phenotype Assays

For arsenite and salt stresses assays, the seeds of Pinku and 20A1 were placed on filter paper within Petri dishes pre‐moistened with distilled water in dark condition. After germination for 5 d, the seeds with consistent growth status were selected for 0.6 µg ml^−1^ As and 150 mm NaCl treatments. The phenotype were daily observed and photographed after one week of As treatment and four days of NaCl treatment. Meanwhile, the root and stem length were measured and counted.

### Identification of Structural Variations (SVs) and Transposable Elements (TEs)

The whole‐genome alignment of Pinku and 20A1 at chromosome‐level was performed by minimap2 (2.26‐r1175) software. The alignment results were analyzed with SyRI (1.6.3) to identify structural variations between the two genomes. Subsequently, BEDTools (v2.27.1) was utilized to determine the genes affected by SVs. The identification of transposable elements (TEs) present in *F. tataricum* (Pinku) and *F. dibotrys* (Luoji Mountain) was conducted using EDTA (v2.1.0).^[^ ^59^ ^]^

### Functional Analysis in Yeast

The yeast strain *Δzrc1* (BY4741; Zn hypersensitive yeast strain; *MATa his3Δ1 leu2Δ0 met15Δ0 ura3Δ0 YMR243c::kanMX4*) was used to investigate the function of *FtYPQ1* in Zn transport. The coding sequence *FtYPQ1* was cloned into pYES2. *OsIRT1* was used as positive control. The recombinant plasmid and empty vector pYES2 were introduced into the yeast strain and grown in SD/‐Ura medium (Coolaber, Beijing, China) at 30 °C for 72 h. Subsequently, they were serially diluted 1 to 10^−4^‐fold onto SD/‐Ura glucose and galactose agar containing ZnCl_2_ (0, 200, and 400 µm), respectively. Petri dishes with spotted yeast cells were placed in an incubator at 30 °C for 2–3 days.

To assess the absorption capacity of Zn, yeast cells in the logarithmic growth period were transferred to 30 ml of SD‐U liquid medium (with galactose as the carbon source) with an initial OD600 value of 0.05 and incubated at 30 °C with 200 rpm. The OD600 values of the liquid culture were measured using a spectrophotometer at different time points (0, 16, 21, 36, 38, and 43 h). Each treatment included three biological replicates. Primer sequences are listed in Table S15 (Supporting Information).

### Overexpressed Plant Construction and Phenotype Assay in Arabidopsis Thaliana

The coding sequence of *FtACA13*, *FtYPQ1*, and *FtNHX2* without termination codon were amplified by using Tartary buckwheat cDNA as the template and inserted into pCAMBIA‐1307 vector. For heterologously expressed *Arabidopsis*, the recombinant plasmids were transformed into *Agrobacterium* strain GV3101. *Arabidopsis* T0 overexpression lines were obtained by *Agrobacterium*‐mediated transformation of *Arabidopsis* flowers (wild type, Columbia ecotype). *Arabidopsis* overexpression materials were screened on MS medium containing 30 µg mL^−1^ hygromycin B and then verified by PCR. T3 lines were used for subsequent experiments.

For phenotypic observation, the wild type, *FtACA13*, *FtYPQ1*, and *FtNHX2* overexpression lines were grown on MS medium supplemented with 100 mm NaCl, 0.2 mm ZnCl_2_, and 0.2 µg ml^−1^ As for three weeks, respectively. The phenotype assays were performed with three replicates. ICP‐MS was used to test the concentrations of Na, Zn, and As. Three biological replicates were conducted. The root length of all lines were measured for three times. All genotypes were grown at 22 °C (day/night) under long‐day conditions (16‐h light/8‐h dark).

### Transgenic Hairy Roots and Phenotype Assay

The recombinant pCAMBIA‐1307 vectors (*FtACA13*‐pCAMBIA‐1307, *FtYPQ1*‐pCAMBIA‐1307) were transformed into *Agrobacterium* A4 to obtain transgenic hairy roots according to methods described previously.^[^ ^60^ ^]^ Subsequently the hairy roots were screened on MS medium containing 30 µg mL^−1^ hygromycin B and identified by PCR and RT‐qPCR.

Phenotype investigation was conducted on the overexpressing hairy roots and wild type after different treatments (50 mm NaCl, 1 mm ZnCl_2_) for three weeks. The fresh weight of all genotypes were measured with three biological replicates. The high performance liquid chromatograph (HPLC, Agilent G6500 Series HPLC‐QTOF) was used for the metabolite content detection of the dried hairy roots following methods described previously.^[^ ^25^, ^28^ ^]^ The experiments were carried out three times. Primer sequences are listed in Table S15 (Supporting Information).

### Total RNA Isolation and Quantitative RT‐PCR

Total RNA from Tartary buckwheat seedlings and hairy roots were extracted by the RNAprep Pure Plant Plus kit (DP441; Tiangen, Beijing, China) according to the manufacturer's protocol. The first‐strand cDNA was synthesized using HiScript III RT SuperMix for qPCR (R323‐01; Vazyme, Nanjing, China) according to the manufacturer's protocol. Quantitative RT‐PCR was conducted using the Light Cycler 480 Real‐Time PCR System (Roche, South San Francisco, CA, USA). The 2^−ΔΔCt^ method and the housekeeping gene *FtACTIN* (FtPinG0302664700) was used to normalize transcript levels and calculate the relative expression of target genes.^[^ ^61^ ^]^ Primer sequences are listed in Table S15 (Supporting Information).

### Phylogenetic Tree Construction

The sequences were obtained from NCBI (https://www.ncbi.nlm.nih.gov/) and *EnsemblPlants* (https://plants.ensembl.org/index.html) using amino acid sequence as the query for the phylogenetic analysis. The phylogenetic tree was conducted using MEGA version 7 based on the neighbor‐joining method.

### Subcellular Localization

The full‐length CDS of *FtACA13*, *FtYPQ1*, and *FtNHX2* were constructed into pCAMBIA‐1300 vector containing a CaMV 35S promoter that drives GFP expression. Recombinant vectors and pJIT‐mCherry‐Mem (cell membrane marker) were transformed into *Agrobacterium tumefaciens* GV3101. DAPI was used for nuclear staining. A laser confocal microscope (LSM900, Zeiss) was used to observe the subcellular localization after transient expression in *N. benthamiana* leaves. Primer sequences are listed in Table S15 (Supporting Information).

### Yeast Two‐Hybrid Assay

The CDS fragments of *FtMYR* was ligated to the pGBKT7 vector (Clontech, Mountain View, CA, USA). The recombinant FtMYR‐pGBKT7 and pGADT7 vectors were then co‐transformed into the yeast strain Y2HGold. The transformed yeast cells were plated on control medium (SD/‐T/‐L) and selective medium (SD/‐T/‐L/‐H/‐A) and incubated for 4 d. Primer sequences are listed in Table S15 (Supporting Information).

### Dual‐Luciferase Assay

The promoter sequences harboring different haplotypes of *FtACA13*, *FtYPQ1*, and *FtNHX2* were cloned into pGreenII‐0800‐miniLUC to detect LUC activity. The CDS fragments of *FtMYR* was cloned into the effector vector pGreen II 62‐SK. Individual combinations of reporter vectors and the effector vector pGreen II 62‐SK under the control of the CaMV 35S promoter were co‐transformed into *A. tumefaciens* strain GV3101 (pSoup‐p19), and the transformed *A. tumefaciens* were used for the infiltration of *N.benthamiana* leaves. After 48–72 h of incubation, the luminescence of the luciferase activity was captured using LB983 Nightowl II, firefly and Renilla luciferase signals were assayed using a Dual Luciferase Reporter Assay Kit (Vazyme, China).^[^ ^62^ ^]^ The experiment was carried out three times. Primer sequences are listed in Table S15 (Supporting Information).

### Electrophoretic Mobility Shift Assay

The full‐length ORFs of *FtMYR* was inserted into pET28a vector and the *FtMYR* protein fused to His tag was expressed in *E. coli* (BL21) and purified with NTA‐Ni Magnetic Agarose Beads according to manufacturer's protocol (P2241, Beyotime, Shanghai, China). The *FtACA13* promoter sequence comprising Hap G was labeled with biotin at 3′‐start to be used as hot probes and the unlabelled sequence was used as cold probes. The promoter sequence without Hap G acted as mutant probes. EMSA reactions were performed by using the Light Shift Chemiluminescent EMSA Kit (Thermo Scientific, Waltham, MA, USA) as described in the manual. DNA probes and primer sequences are listed in Table S15 (Supporting Information).

### Ca^2+^ Flux Measurement with Non‐Invasive Micro‐Test Technology (NMT)

The Ca^2+^ and Zn^2+^ fluxes at the root surface were measured using NMT (NMT‐YG‐100; Younger USA, Amherst, MA, USA; Xuyue (Beijing) Sci. & Tech. Co. Ltd, Beijing, China).^[^ ^63^ ^]^ The Ca^2+^ and Zn^2+^ fluxes were examined in a zone ≈400 µm from the root tip of each genotypic materials with six biological replicates.

### Statistical Analysis

The maps of geographical distribution of elements and haplotypes were made with ArcGIS software (version 10.2). The QTLs distribution on chromosomes were obtained through TBtools (II),^[^ ^64^ ^]^ and the phenotypic correlations between ionomic traits and histogram were achieved using Origin software (version 2022) on the basis of Spearman's correlation coefficients. All the violin, box, and column diagram were obtained by GraphPad Prism 10 software. Significant differences between two groups were assessed using two‐tailed Student's *t*‐test. For comparisons of multiple groups, one‐way analysis of variance (ANOVA) was performed. Significance levels were defined as * *P* < 0.05; ** *P* < 0.01; and *** *P* < 0.001.

## Conflict of Interest

The authors declare no conflict of interest.

## Author Contributions

Z.W., Y.H., M.Z., X.L., and H.L. contributed equally to this work. M.Z. and X.H. designed and managed the project. M.Z., Z.W., and Y.H. organized the funding for this research. S.H.W., M.Q., and M.Z. provided the genetic materials. Z.W., Y.H., M.Z., X.L., and H.L. performed data analysis and figure design. Z.W., Y.S., T.L., J.H., D.L., X.P., X.W., and W.L. performed most of the experiments. Z.W., K.Z., G.L., X.H., A.R.F., and M.Z wrote the manuscript. All authors read and approved the manuscript.

## Supporting information



Supporting Information

Supplemental Tables

## Data Availability

The data that support the findings of this study are available in the supplementary material of this article.

## References

[1] D. E. Salt , I. Baxter , B. Lahner , Annu. Rev. Plant Biol. 2008, 59, 709. 18251712 10.1146/annurev.arplant.59.032607.092942

[2] S. B. Singh , K. Singh , S. S. Butola , S. Rawat , K. Arunachalam , Nat. Prod. Sci. 2020, 26, 50.

[3] C. Stanton , D. Sanders , U. Kramer , D. Podar , Mol. Plant 2022, 15, 65. 34952215 10.1016/j.molp.2021.12.008

[4] P. J. Aggett , B. P. Marriott , D. F. Birt , V. A. Stallings , A. A. Yates , Present Knowledge in Nutrition, Academic Press, London 2020, p. 375.

[5] P. Yan , Q. Du , H. Chen , Z. Guo , Z. Wang , J. Tang , W. X. Li , Science 2023, 382, 1159. 38060668 10.1126/science.adf3256

[6] Y. Liu , B. Jiao , W. Qian , Mod. Agric. 2024, 2, 30.

[7] J. Li , C. Martin , A. Fernie , Nat. Food 2024, 5, 19. 38168782 10.1038/s43016-023-00905-8

[8] S. Atwell , Y. S. Huang , B. J. Vilhjalmsson , G. Willems , M. Horton , Y. Li , D. Meng , A. Platt , A. M. Tarone , T. T. Hu , R. Jiang , N. W. Muliyati , X. Zhang , M. A. Amer , I. Baxter , B. Brachi , J. Chory , C. Dean , M. Debieu , J. de Meaux , J. R. Ecker , N. Faure , J. M. Kniskern , J. D. Jones , T. Michael , A. Nemri , F. Roux , D. E. Salt , C. Tang , M. Todesco , et al., Nature 2010, 465, 627. 20336072 10.1038/nature08800 PMC3023908

[9] A. Campos , W. F. A. van Dijk , P. Ramakrishna , T. Giles , P. Korte , A. Douglas , P. Smith , D. E. Salt , Plant J. 2021, 106, 536. 33506585 10.1111/tpj.15177

[10] M. Yang , K. Lu , F. J. Zhao , W. Xie , P. Ramakrishna , G. Wang , Q. Du , L. Liang , C. Sun , H. Zhao , Z. Zhang , Z. Liu , J. Tian , X. Y. Huang , W. Wang , H. Dong , J. Hu , L. Ming , Y. Xing , G. Wang , J. Xiao , D. E. Salt , X. Lian , Plant Cell 2018, 30, 2720. 30373760 10.1105/tpc.18.00375 PMC6305983

[11] E. A. Pasion , G. Misra , A. Kohli , N. Sreenivasulu , Plant J. 2023, 113, 749. 36573652 10.1111/tpj.16080 PMC10952705

[12] N. D. Rathan , H. Krishna , R. K. Ellur , D. Sehgal , V. Govindan , A. K. Ahlawat , G. Krishnappa , J. P. Jaiswal , J. B. Singh , S. Sv , D. Ambati , S. K. Singh , K. Bajpai , A. Mahendru‐Singh , Sci. Rep. 2022, 12, 7037. 35487909 10.1038/s41598-022-10618-w PMC9054743

[13] B. Stich , A. Benke , M. Schmidt , C. Urbany , R. Shi , N. von Wiren , Plant Cell Environ. 2020, 43, 2095. 32529648 10.1111/pce.13823

[14] M. W. Blair , C. Astudillo , M. Grusak , R. Graham , S. E. Beebe , Mol. Breed. 2009, 23, 197.

[15] N. Shakoor , G. Ziegler , B. P. Dilkes , Z. Brenton , R. Boyles , E. L. Connolly , S. Kresovich , I. Baxter , Plant Physiol. 2016, 170, 1989. 26896393 10.1104/pp.15.01971 PMC4825124

[16] A. Bus , N. Korber , I. A. Parkin , B. Samans , R. J. Snowdon , J. Li , B. Stich , Front. Plant Sci. 2014, 5, 485. 25324847 10.3389/fpls.2014.00485 PMC4179769

[17] H. V. Hunt , X. Shang , M. K. Jones , Veg. Hist. Archaeobotany 2018, 27, 493. 10.1007/s00334-017-0649-4 PMC6560938 31258253

[18] H. Lin , Y. Yao , P. Sun , L. Feng , S. Wang , Y. Ren , X. Yu , Z. Xi , J. Liu , BMC Biol. 2023, 21, 87. 37069628 10.1186/s12915-023-01587-1 PMC10111841

[19] W. Yang , H. Duan , K. Yu , S. Hou , Y. Kang , X. Wang , J. Hao , L. Liu , Y. Zhang , L. Luo , Y. Zhao , J. Zhang , C. Lan , N. Wang , X. Zhang , J. Tang , Q. Zhao , Z. Sun , X. Zhang , Adv. Sci. 2024, 11, 20. 10.1002/advs.202400916 PMC11132045 38520733

[20] K. Zhang , M. He , Y. Fan , H. Zhao , B. Gao , K. Yang , F. Li , Y. Tang , Q. Gao , T. Lin , M. Quinet , D. Janovska , V. Meglic , J. Kwiatkowski , O. Romanova , N. Chrungoo , T. Suzuki , Z. Luthar , M. Germ , S. H. Woo , M. I. Georgiev , M. Zhou , Genome Biol. 2021, 22, 23. 33430931 10.1186/s13059-020-02217-7 PMC7802136

[21] Y. He , K. Zhang , Y. Shi , H. Lin , X. Huang , X. Lu , Z. Wang , W. Li , X. Feng , T. Shi , Q. Chen , J. Wang , Y. Tang , M. A. Chapman , M. Germ , Z. Luthar , I. Kreft , D. Janovska , V. Meglic , S. H. Woo , M. Quinet , A. R. Fernie , X. Liu , M. Zhou , Genome Biol. 2024, 25, 61. 38414075 10.1186/s13059-024-03203-z PMC10898187

[22] L. Zhang , X. Li , B. Ma , Q. Gao , H. Du , Y. Han , Y. Li , Y. Cao , M. Qi , Y. Zhu , H. Lu , M. Ma , L. Liu , J. Zhou , C. Nan , Y. Qin , J. Wang , L. Cui , H. Liu , C. Liang , Z. Qiao , Mol. Plant 2017, 10, 1224. 28866080 10.1016/j.molp.2017.08.013

[23] A. Kumari , H. K. Chaudhary , Crit. Rev. Biotechnol. 2020, 40, 539. 32290728 10.1080/07388551.2020.1747387

[24] X. Y. Huang , D. E. Salt , Mol. Plant 2016, 9, 787. 27212388 10.1016/j.molp.2016.05.003

[25] H. Zhao , Y. He , K. Zhang , S. Li , Y. Chen , M. He , F. He , B. Gao , D. Yang , Y. Fan , X. Zhu , M. Yan , N. Giglioli‐Guivarc'h , C. Hano , A. R. Fernie , M. I. Georgiev , D. Janovska , V. Meglic , M. Zhou , Plant Biotechnol. J. 2023, 21, 150. 36148785 10.1111/pbi.13932 PMC9829391

[26] U. M. Sainju , D. Liptzin , J. D. Jabro , Sci. Rep. 2022, 12, 22025. 36539542 10.1038/s41598-022-26619-8 PMC9768131

[27] W. Shangguan , Y. Dai , B. Liu , A. Zhu , Q. Duan , L. Wu , D. Ji , A. Ye , H. Yuan , Q. Zhang , J. Adv. Model Earth Syst. 2013, 5, 212.

[28] D. Lai , K. Zhang , Y. He , Y. Fan , W. Li , Y. Shi , Y. Gao , X. Huang , J. He , H. Zhao , X. Lu , Y. Xiao , J. Cheng , J. Ruan , M. I. Georgiev , A. R. Fernie , M. Zhou , Plant Biotechnol. J. 2024, 22, 1206. 38062934 10.1111/pbi.14259 PMC11022807

[29] H. Fu , X. Yu , Y. Jiang , Y. Wang , Y. Yang , S. Chen , Q. Chen , Y. Guo , Plant Cell 2023, 35, 279. 36149299 10.1093/plcell/koac283 PMC9806586

[30] S. Ray , R. Gaudet , Biochem. Soc. Trans. 2023, 51, 897. 37283482 10.1042/BST20210699 PMC10330786

[31] X. Ye , C. Liu , H. Yan , Y. Wan , Q. Wu , X. Wu , G. Zhao , L. Zou , D. Xiang , Gene 2022, 847, 146884. 36103913 10.1016/j.gene.2022.146884

[32] L. Shi , J. Su , M. J. Cho , H. Song , X. Dong , Y. Liang , Z. Zhang , J. Exp. Bot. 2023, 74, 4349. 37204916 10.1093/jxb/erad175

[33] A. Gradogna , J. Scholz‐Starke , J. M. Pardo , A. Carpaneto , New Phytol. 2021, 229, 3026. 33098586 10.1111/nph.17021

[34] S. Hendrix , Plant Cell 2023, 35, 1962. 36857081 10.1093/plcell/koad052 PMC10226585

[35] A. Costa , F. Resentini , S. Buratti , M. C. Bonza , Biochim. Biophys. Acta. Mol. Cell Res. 2023, 1870, 119508. 37290725 10.1016/j.bbamcr.2023.119508

[36] F. E. Tracy , M. Gilliham , A. N. Dodd , A. A. Webb , M. Tester , Plant Cell Environ. 2008, 31, 1063. 18419736 10.1111/j.1365-3040.2008.01817.x

[37] M. Liu , Z. Ma , T. Zheng , W. Sun , Y. Zhang , W. Jin , J. Zhan , Y. Cai , Y. Tang , Q. Wu , Z. Tang , T. Bu , C. Li , H. Chen , BMC Genomics 2018, 19, 648. 30170551 10.1186/s12864-018-5036-8 PMC6119279

[38] K. M. Huda , M. S. Banu , B. Garg , S. Tula , R. Tuteja , N. Tuteja , Plant J. 2013, 76, 997. 24128296 10.1111/tpj.12352

[39] M. Sun , B. Jia , N. Cui , Y. Wen , H. Duanmu , Q. Yu , J. Xiao , X. Sun , Y. Zhu , Plant Mol. Biol. 2016, 90, 419. 26801329 10.1007/s11103-015-0426-7

[40] D. Hu , Q. Ma , C. Sun , M. Sun , C. You , Y. Hao , Physiol. Plant 2016, 156, 201. 26096498 10.1111/ppl.12354

[41] J. Abramson , J. Adler , J. Dunger , R. Evans , T. Green , A. Pritzel , O. Ronneberger , L. Willmore , A. J. Ballard , J. Bambrick , S. W. Bodenstein , D. A. Evans , C. C. Hung , M. O'Neill , D. Reiman , K. Tunyasuvunakool , Z. Wu , A. Zemgulyte , E. Arvaniti , C. Beattie , O. Bertolli , A. Bridgland , A. Cherepanov , M. Congreve , A. I. Cowen‐Rivers , A. Cowie , M. Figurnov , F. B. Fuchs , H. Gladman , R. Jain , et al., Nature 2024, 630, 493. 38718835 10.1038/s41586-024-07487-w PMC11168924

[42] J. S. Khokhar , J. King , I. P. King , S. D. Young , M. J. Foulkes , J. De Silva , M. Weerasinghe , A. Mossa , S. Griffiths , A. B. Riche , M. Hawkesford , P. Shewry , M. R. Broadley , PLoS One 2020, 15, 0229107. 10.1371/journal.pone.0229107 PMC7048275 32109944

[43] S. Lee , G. An , Plant Cell Environ. 2009, 32, 408. 19183299 10.1111/j.1365-3040.2009.01935.x

[44] J. Li , M. S. Hossain , H. Ma , Q. Yang , X. Gong , P. Yang , B. Feng , J. Food Compos. Anal. 2020, 85, 103335.

[45] Y. Wei , M. Guo , Biol. Trace Elem. Res. 2014, 161, 223. 25123463 10.1007/s12011-014-0099-0

[46] M. He , Y. He , K. Zhang , X. Lu , X. Zhang , B. Gao , Y. Fan , H. Zhao , R. Jha , M. N. Huda , Y. Tang , J. Wang , W. Yang , M. Yan , J. Cheng , J. Ruan , E. Dulloo , Z. Zhang , M. I. Georgiev , M. A. Chapman , M. Zhou , New Phytol. 2022, 235, 1927. 35701896 10.1111/nph.18306

[47] H. Du , C. Liang , Nat. Commun. 2019, 10, 5360. 31767853 10.1038/s41467-019-13355-3 PMC6877557

[48] M. Cornejo , D. Mieres‐Castro , E. H. Blanco , A. R. Beltran , J. E. Araya , G. Fuentes , M. Figueroa , C. Labarca , F. Toledo , M. A. Ramirez , L. Sobrevia , Biochim. Biophys. Acta. Mol. Basis. Dis. 1867, 2021, 165977. 10.1016/j.bbadis.2020.165977 32980460

[49] J. Li , Y. Guo , Y. Yang , J. Genet. Genomics 2022, 49, 715. 35654346 10.1016/j.jgg.2022.05.007

[50] L. Liu , B. Wang , Biology 2021, 10, 353.33922035

[51] S. Huang , P. Wei , Mod. Agric. 2024, 2, e70002.

[52] Y. Chen , Z. Chao , M. Jin , Y. Wang , Y. Li , J. Wu , Y. Xiao , Y. Peng , Q. Lv , S. Gui , X. Wang , M. Han , A. Fernie , D. Chao , J. Yan , J. Genet. Genomics 2023, 50, 130. 36028132 10.1016/j.jgg.2022.08.003

[53] B. Lv , Q. Wu , A. Wang , Q. Li , Q. Dong , J. Yang , H. Zhao , X. Wang , H. Chen , C. Li , Plant Physiol. Biochem. 2020, 147, 43. 31841961 10.1016/j.plaphy.2019.12.004

[54] M. Manzoor , A. Hami , M. Pakhtoon , A. Batool , A. Zaffar , J. Sudan , G. Ali , M. Khan , P. Sofi , R. Mahajan , B. Bhat , R. Mushtaq , N. Sofi , M. Bhat , S. Zargar , Nucleus 2024, 67, 331.

[55] A. Pinski , A. Betekhtin , Front. Plant Sci. 2023, 14, 1270150. 37746024 10.3389/fpls.2023.1270150 PMC10515086

[56] M. Daniel‐Mozo , B. Rombolá‐Caldentey , I. Mendoza , P. Ragel , A. De Luca , R. Carranco , A. M. Alcaide , A. Ausili , B. Cubero , K. Schumacher , F. J. Quintero , A. Albert , J. M. Pardo , Sci. Adv. 2024, 15, 10. 10.1126/sciadv.adp7658 PMC11559620 39536104

[57] X. Huang , F. Deng , N. Yamaji , S. R. Pinson , M. Fujii‐Kashino , J. Danku , A. Douglas , M. L. Guerinot , D. E. Salt , J. Ma , Nat. Commun. 2016, 7, 12138. 27387148 10.1038/ncomms12138 PMC4941113

[58] C. Lippert , J. Listgarten , Y. Liu , C. M. Kadie , R. I. Davidson , D. Heckerman , Nat. Methods 2011, 8, 833. 21892150 10.1038/nmeth.1681

[59] S. Ou , W. Su , Y. Liao , K. Chougule , J. R. A. Agda , A. J. Hellinga , C. S. B. Lugo , T. A. Elliott , D. Ware , T. Peterson , N. Jiang , C. N. Hirsch , M. B. Hufford , Genome Biol. 2019, 20, 275. 31843001 10.1186/s13059-019-1905-y PMC6913007

[60] X. Huang , Y. He , K. Zhang , Y. Shi , H. Zhao , D. Lai , H. Lin , X. Wang , Z. Yang , Y. Xiao , W. Li , Y. Ouyang , S. H. Woo , M. Quinet , M. I. Georgiev , A. R. Fernie , X. Liu , M. Zhou , Adv. Sci. 2024, 11, 2403603. 10.1002/advs.202403603 PMC11578379 39312476

[61] B. Ma , X. Cao , X. Li , Z. Bian , Q. Zhang , Z. Fang , J. Liu , Q. Li , Q. Liu , L. Zhang , Z. He , J. Genet. Genomics 2024, 51, 492. 37913986 10.1016/j.jgg.2023.10.007

[62] Z. Wang , M. Gao , Y. Li , J. Zhang , H. Su , M. Cao , Z. Liu , X. Zhang , B. Zhao , Y. D. Guo , N. Zhang , J. Exp. Bot. 2022, 73, 6207. 35696674 10.1093/jxb/erac258

[63] J. Wang , Y. Ren , X. Liu , S. Luo , X. Zhang , X. Liu , Q. Lin , S. Zhu , H. Wan , Y. Yang , Y. Zhang , B. Lei , C. Zhou , T. Pan , Y. Wang , M. Wu , R. Jing , Y. Xu , M. Han , F. Wu , C. Lei , X. Guo , Z. Cheng , X. Zheng , Y. Wang , Z. Zhao , L. Jiang , X. Zhang , Y. Wang , H. Wang , et al., Mol. Plant 2021, 14, 315. 33278597 10.1016/j.molp.2020.11.022

[64] C. Chen , Y. Wu , J. Li , X. Wang , Z. Zeng , J. Xu , Y. Liu , J. Feng , H. Chen , Y. He , R. Xia , Mol. Plant 2023, 16, 1733. 37740491 10.1016/j.molp.2023.09.010

