# Supplementary material for: Genome‐Wide Association Studies Reveal the Genetic Architecture of Ionomic Variation in Grains of Tartary Buckwheat
Source: Adv Sci (Weinh). 2025 Mar 13;12(17):2412291. doi: 10.1002/advs.202412291 (PMC12061317; doi:10.1002/advs.202412291)
Supplement: Supplementary file 1 — Supporting Information [file ADVS-12-2412291-s002.pdf]

ADVANCED SCIENCE
Open Access

Supporting Information

for *Adv. Sci.*, DOI 10.1002/advs.202412291

Genome-Wide Association Studies Reveal the Genetic Architecture of Ionomic Variation in Grains of Tartary Buckwheat

Zhirong Wang, Yuqi He, Mengyu Zhao, Xiang-Qian Liu, Hao Lin, Yaliang Shi, Kaixuan Zhang, Guijie Lei, Dili Lai, Tong Liu, Xiaoyang Peng, Jiayue He, Wei Li, Xiangru Wang, Sun-Hee Woo, Muriel Quinet, Alisdair R. Fernie, Xin-Yuan Huang and Meiliang Zhou**

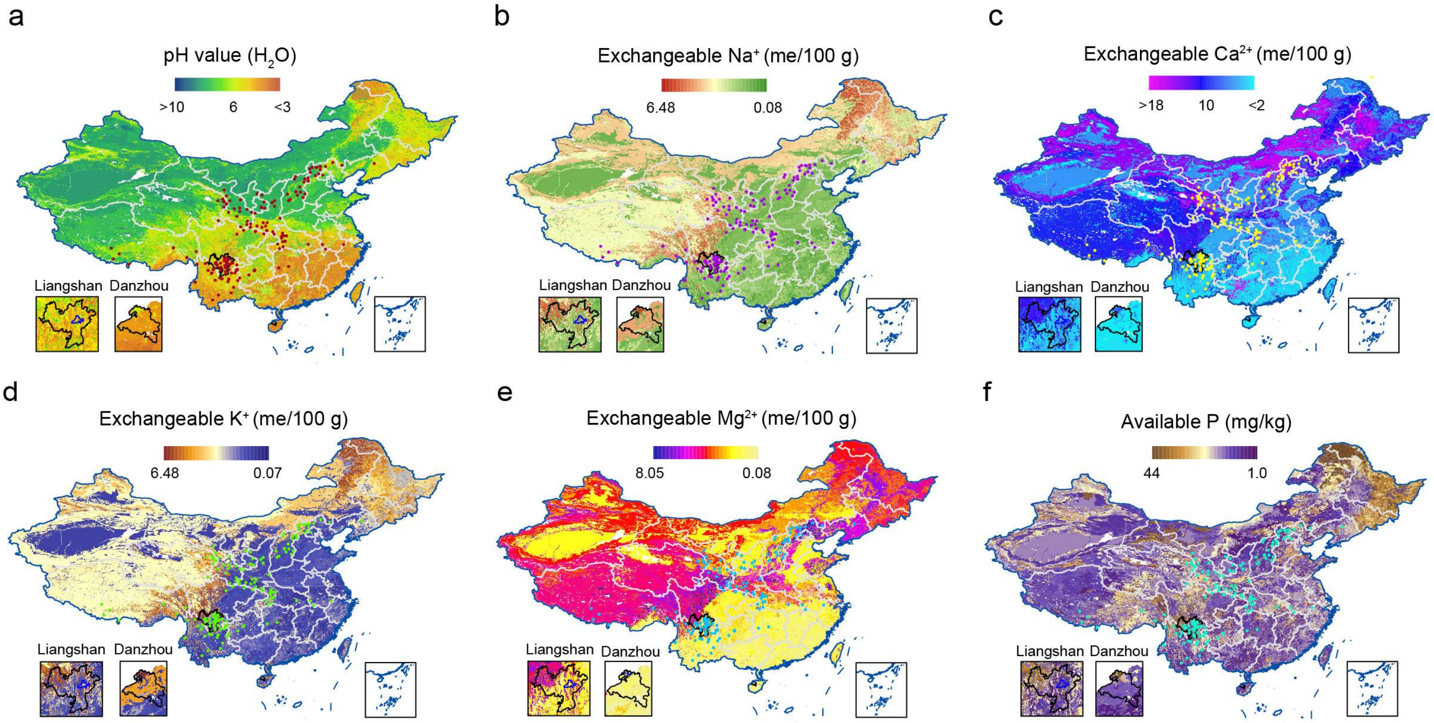

Figure S1. The soil properties of planting field. a-f. Spatial distribution of soil pH value (a), exchangeable Na^+ (me/100 g) (b), exchangeable Ca^{2+} (me/100 g) (c), exchangeable K^+ (me/100 g) (d), exchangeable Mg^{2+} (me/100 g) (e), available phosphorus (P, mg/kg) (f) in China. The circles marked with different colors represent the geographical distribution of 182 Tartary buckwheat accessions. layer (4.5-9.1 cm). The two rectangular boxes in the bottom left corner of the map represent Liangshan Yi Autonomous Prefecture and Danzhou City, respectively, with the blue box in the Liangshan map representing Zhaojue County.

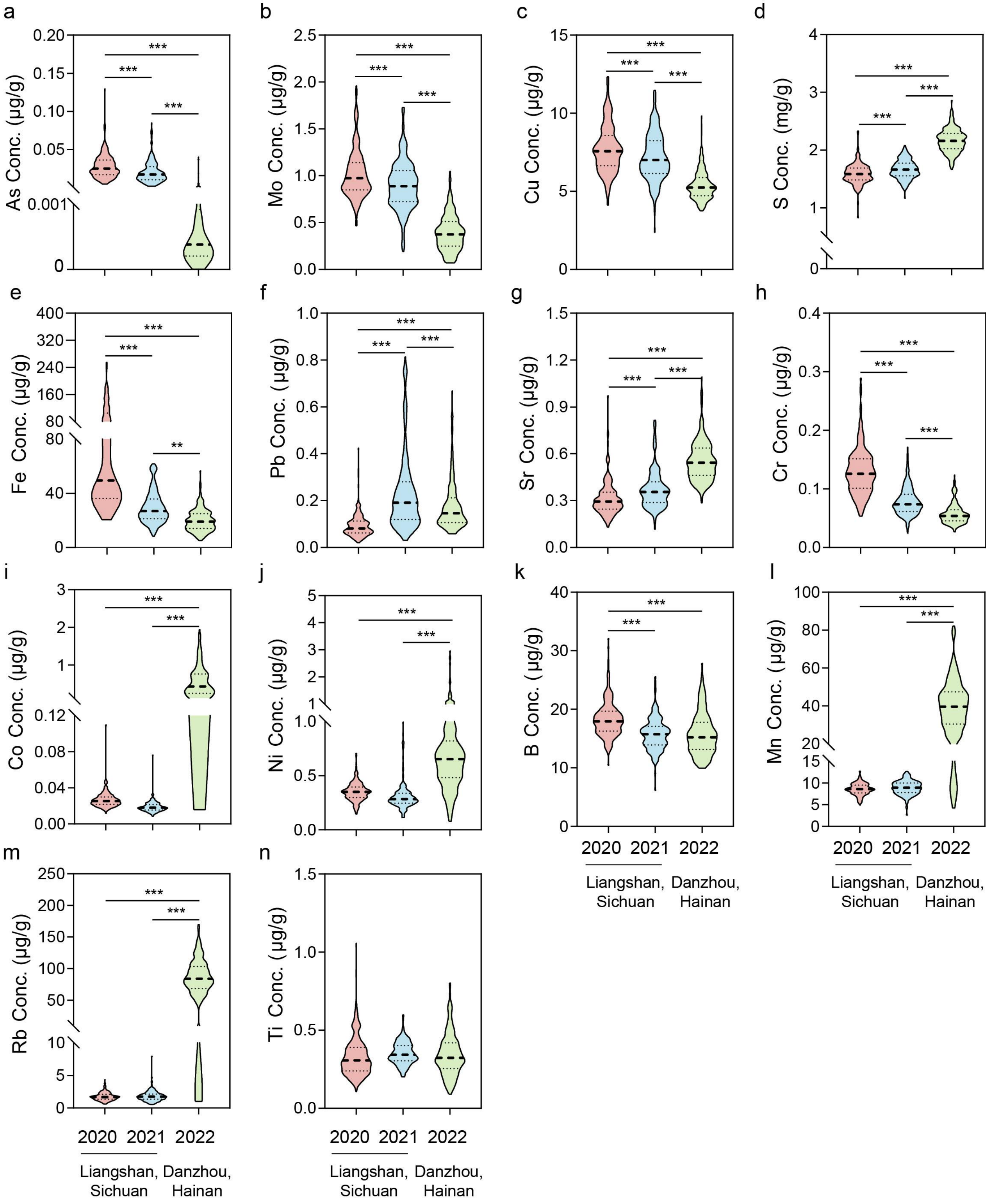

Figure S2. Violin plots of 14 elemental concentrations of Tartary buckwheat seeds among different years and locations. Asterisk (**) and (***) indicate significant difference at $P < 0.01$ and $P < 0.001$ using one-way ANOVA.

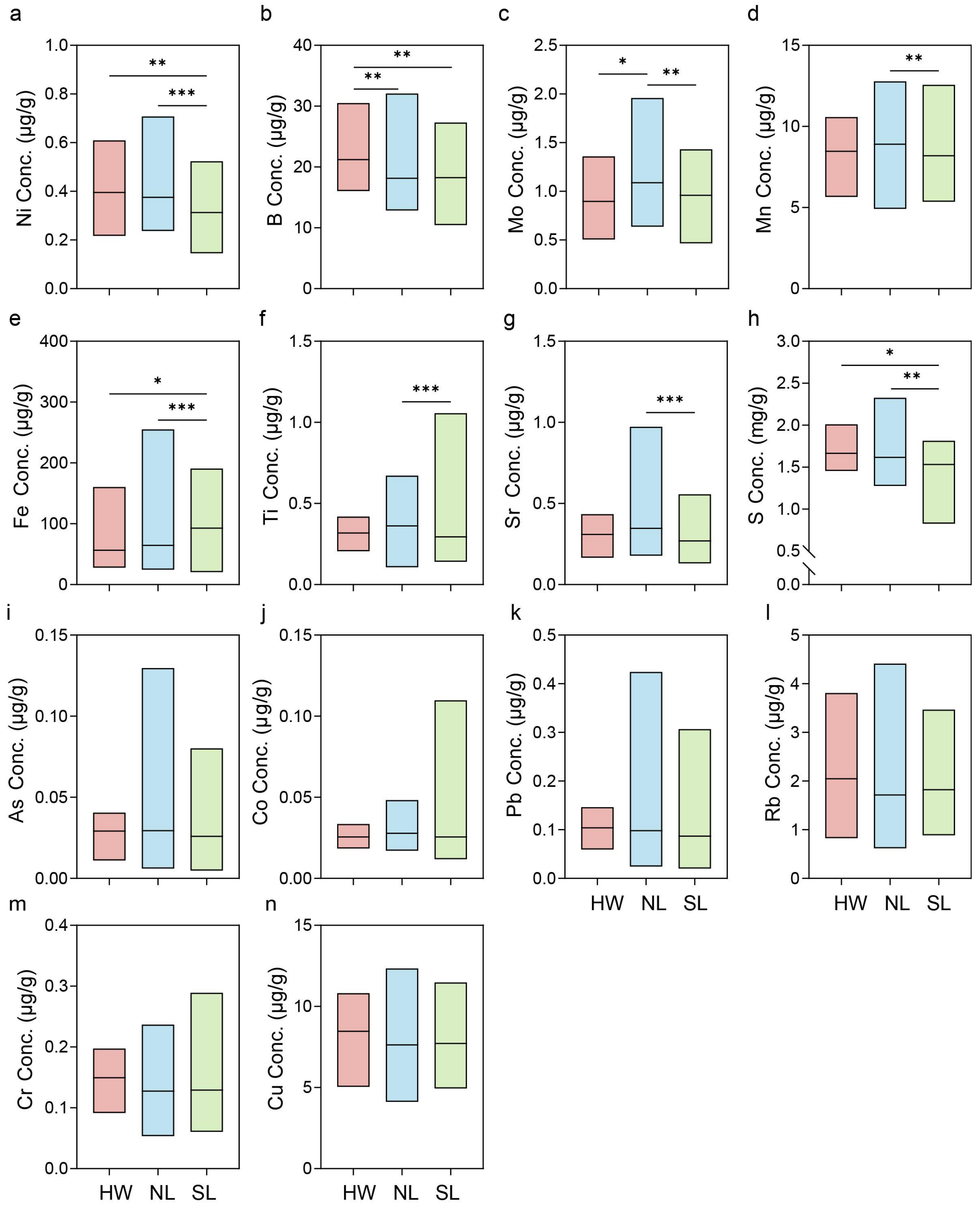

Figure S3. Box plots of 14 elemental concentrations of Tartary buckwheat seeds among three groups. Asterisk (**) and (***) indicate significant difference at $P < 0.01$ and $P < 0.001$ using one-way ANOVA.

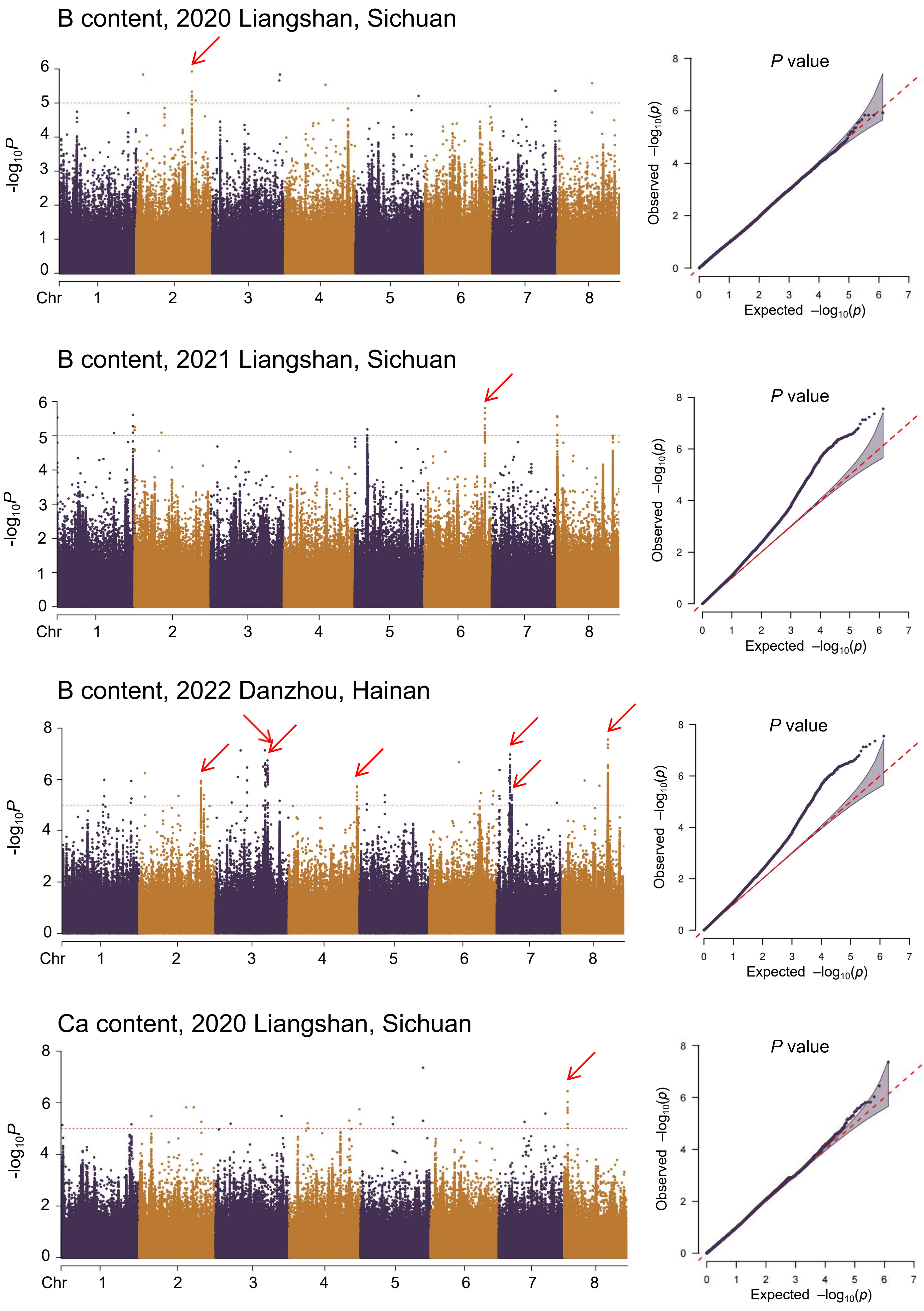

B content, 2020 Liangshan, Sichuan
-log10P
Chr
1
2
3
4
5
6
7
8
P value
Observed −log10(p)
Expected −log10(p)
B content, 2021 Liangshan, Sichuan
-log10P
Chr
P value
Observed −log10(p)
Expected −log10(p)
B content, 2022 Danzhou, Hainan
-log10P
Chr
P value
Observed −log10(p)
Expected −log10(p)
Ca content, 2020 Liangshan, Sichuan
-log10P
Chr
P value
Observed −log10(p)
Expected −log10(p)

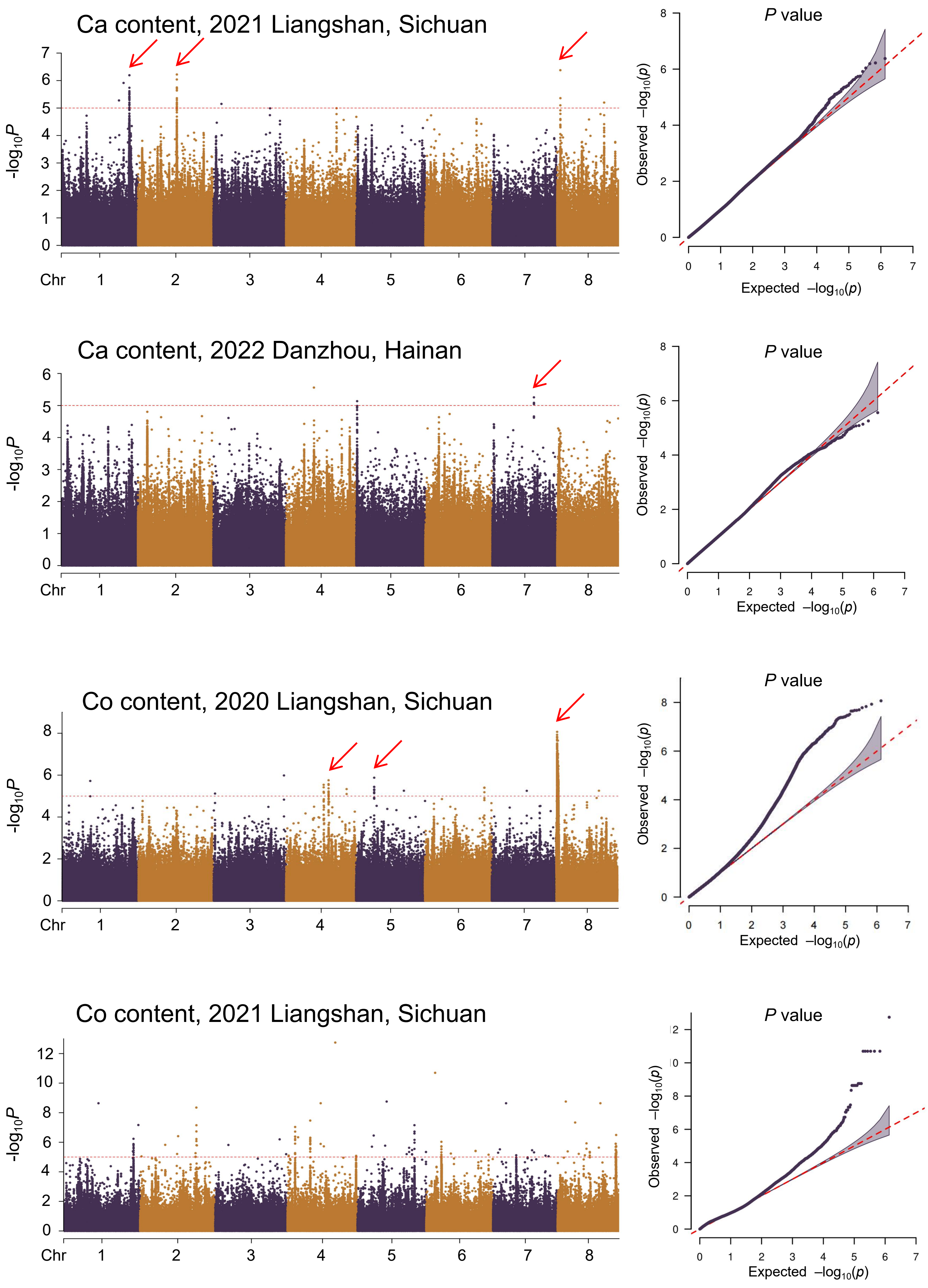

Ca content, 2021 Liangshan, Sichuan
Ca content, 2022 Danzhou, Hainan
Co content, 2020 Liangshan, Sichuan
Co content, 2021 Liangshan, Sichuan
-log10P
Chr
P value
Observed −log10(p)
Expected −log10(p)

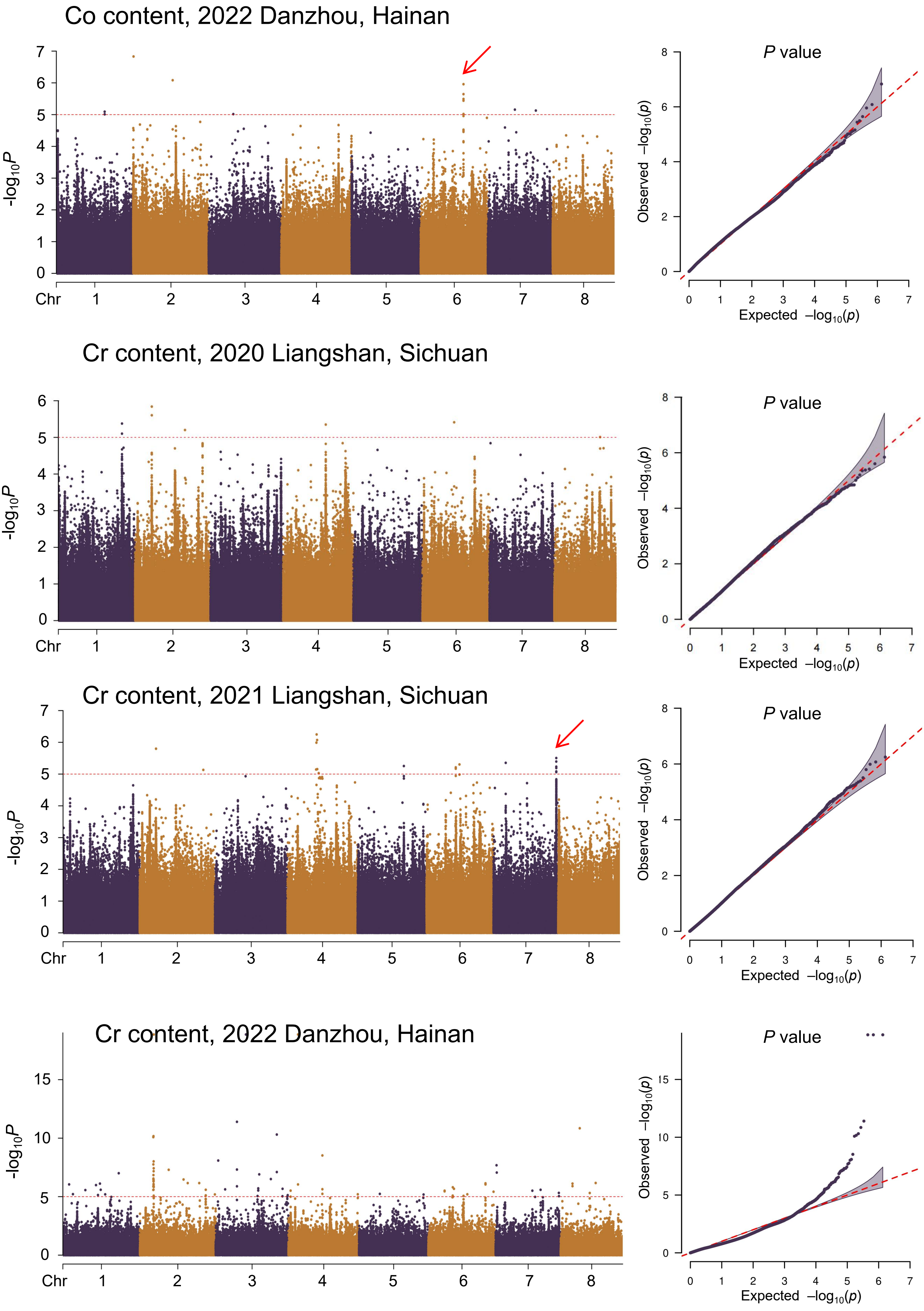
Co content, 2022 Danzhou, Hainan
Cr content, 2020 Liangshan, Sichuan
Cr content, 2021 Liangshan, Sichuan
Cr content, 2022 Danzhou, Hainan
$-\log_{10}P$
Chr
1
2
3
4
5
6
7
8
P value
Observed $-\log_{10}(p)$
Expected $-\log_{10}(p)$

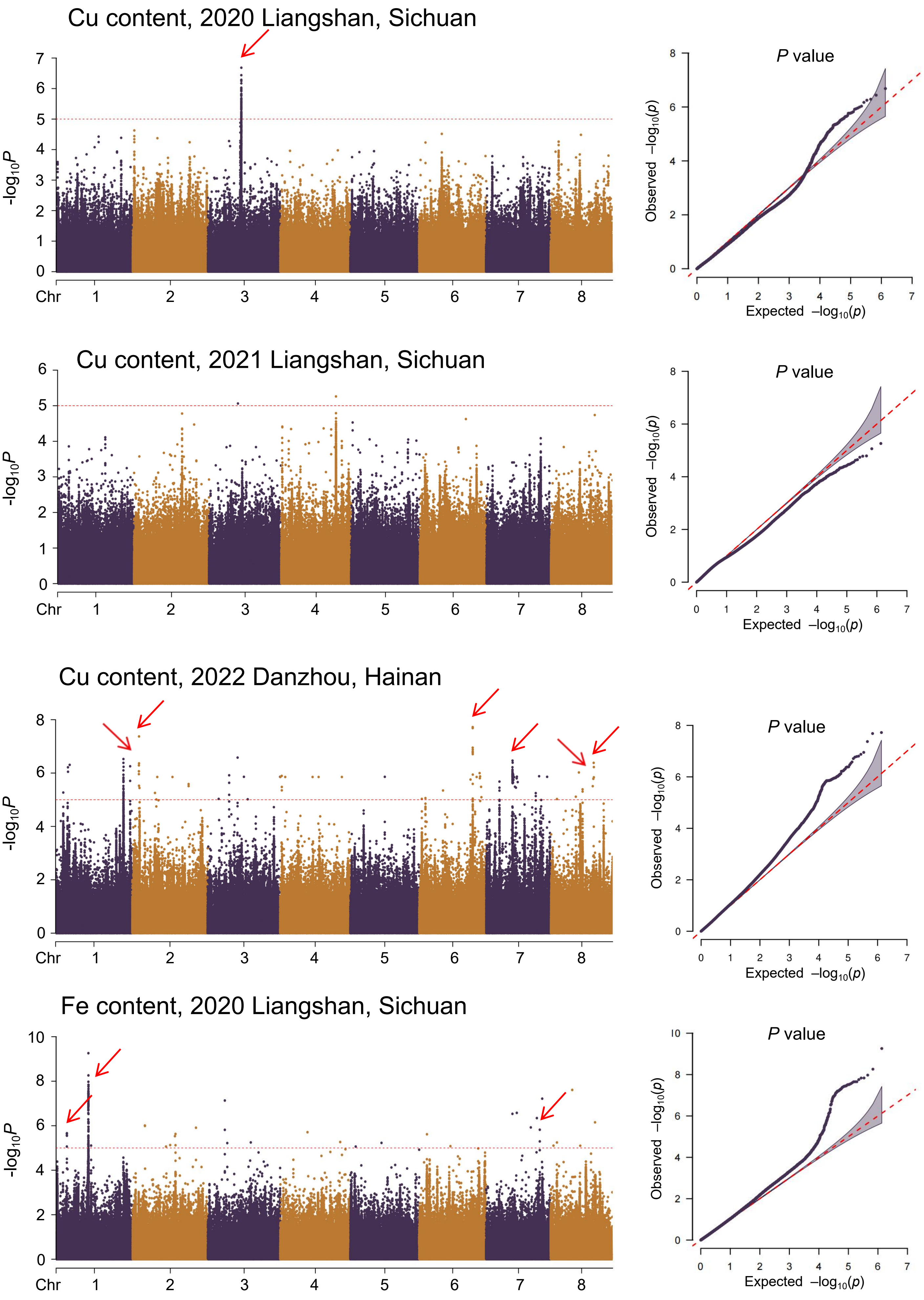

Cu content, 2020 Liangshan, Sichuan
Cu content, 2021 Liangshan, Sichuan
Cu content, 2022 Danzhou, Hainan
Fe content, 2020 Liangshan, Sichuan
$-\log_{10}P$
Chr
1 2 3 4 5 6 7 8
P value
Observed $-\log_{10}(p)$
Expected $-\log_{10}(p)$

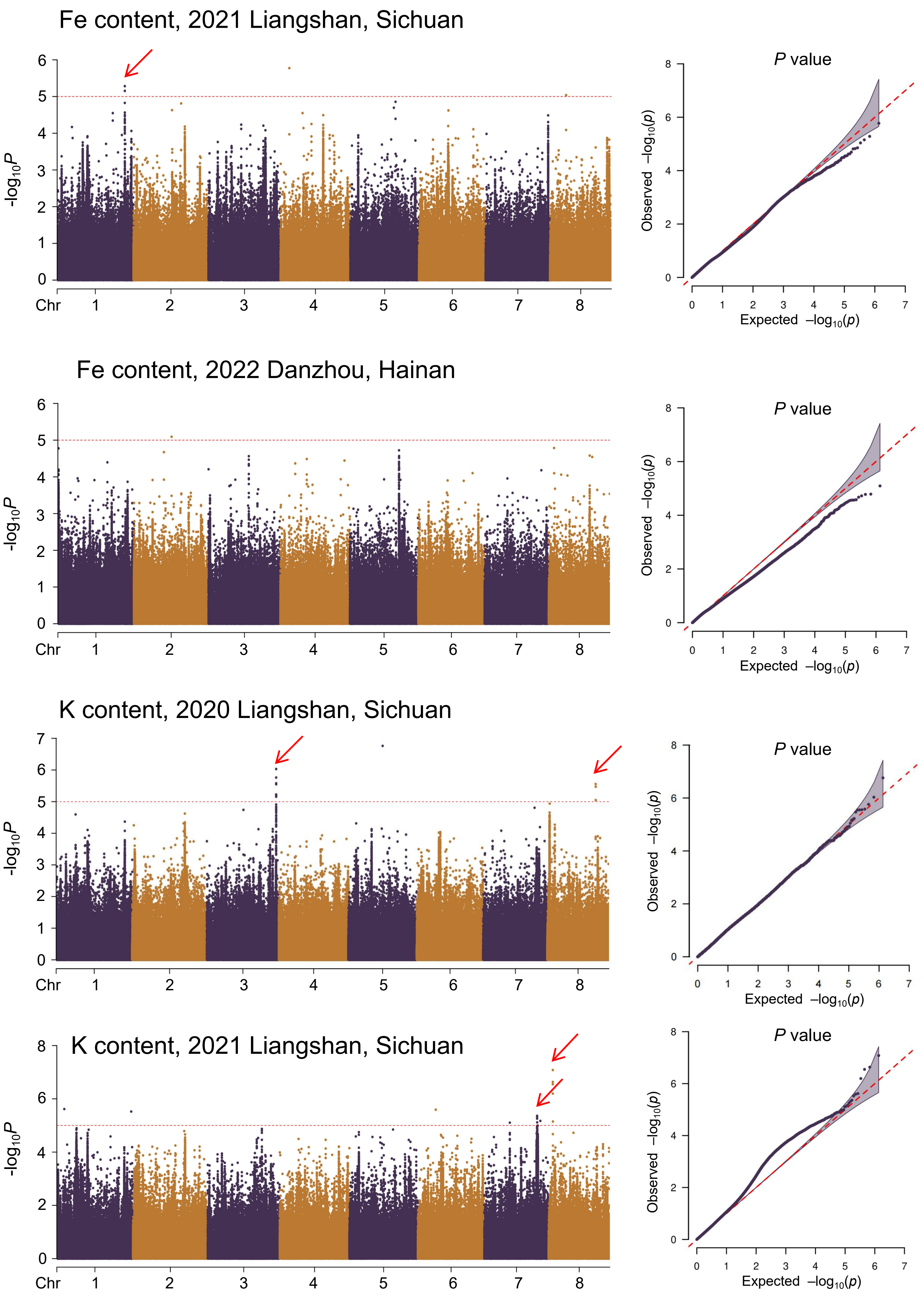
Fe content, 2021 Liangshan, Sichuan
-log10P
Chr
P value
Observed –log10(p)
Expected –log10(p)
Fe content, 2022 Danzhou, Hainan
-log10P
Chr
P value
Observed –log10(p)
Expected –log10(p)
K content, 2020 Liangshan, Sichuan
-log10P
Chr
P value
Observed –log10(p)
Expected –log10(p)
K content, 2021 Liangshan, Sichuan
-log10P
Chr
P value
Observed –log10(p)
Expected –log10(p)

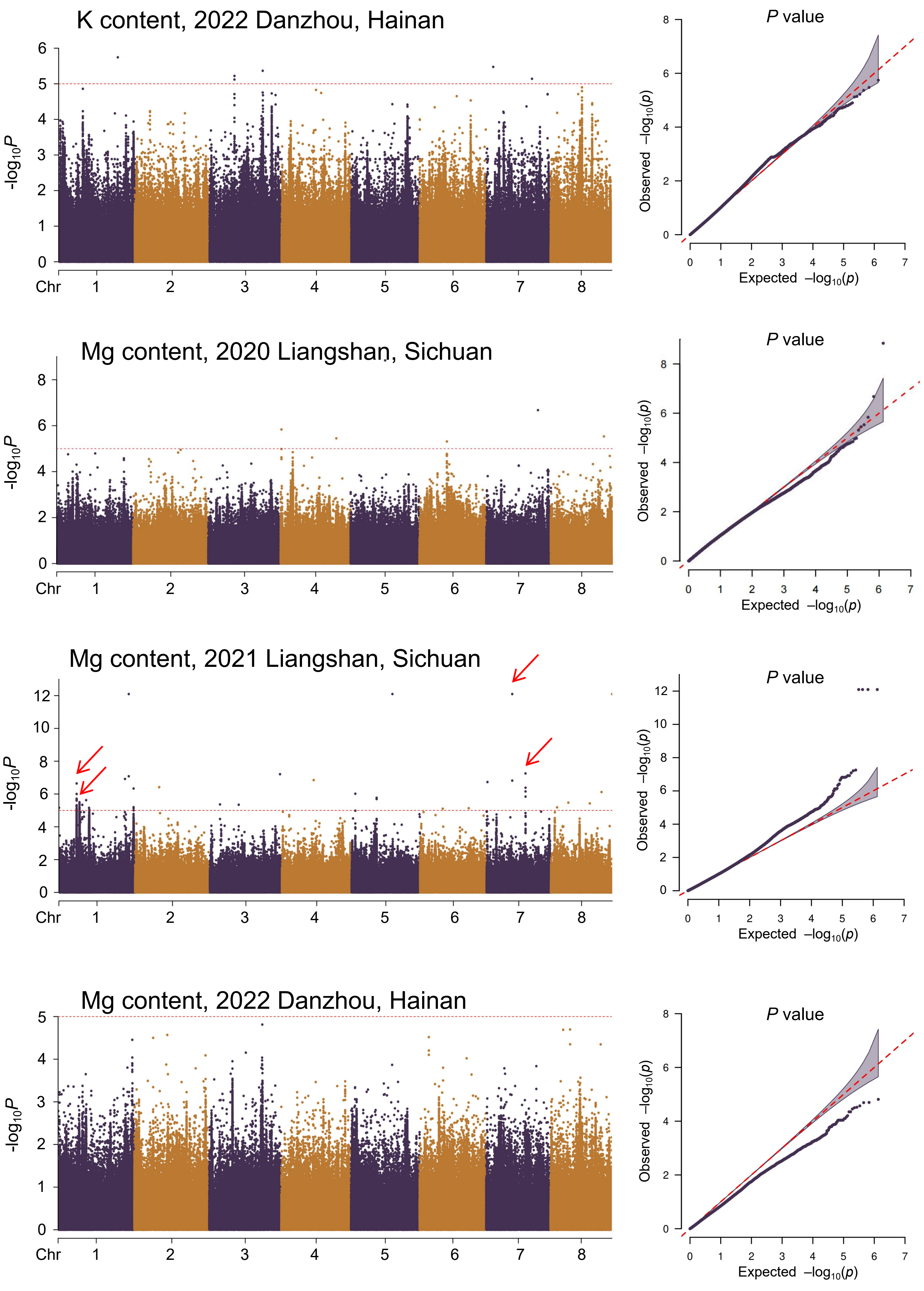

K content, 2022 Danzhou, Hainan
Mg content, 2020 Liangshan, Sichuan
Mg content, 2021 Liangshan, Sichuan
Mg content, 2022 Danzhou, Hainan
-log10P
Chr
1
2
3
4
5
6
7
8
P value
Observed –log10(p)
Expected –log10(p)

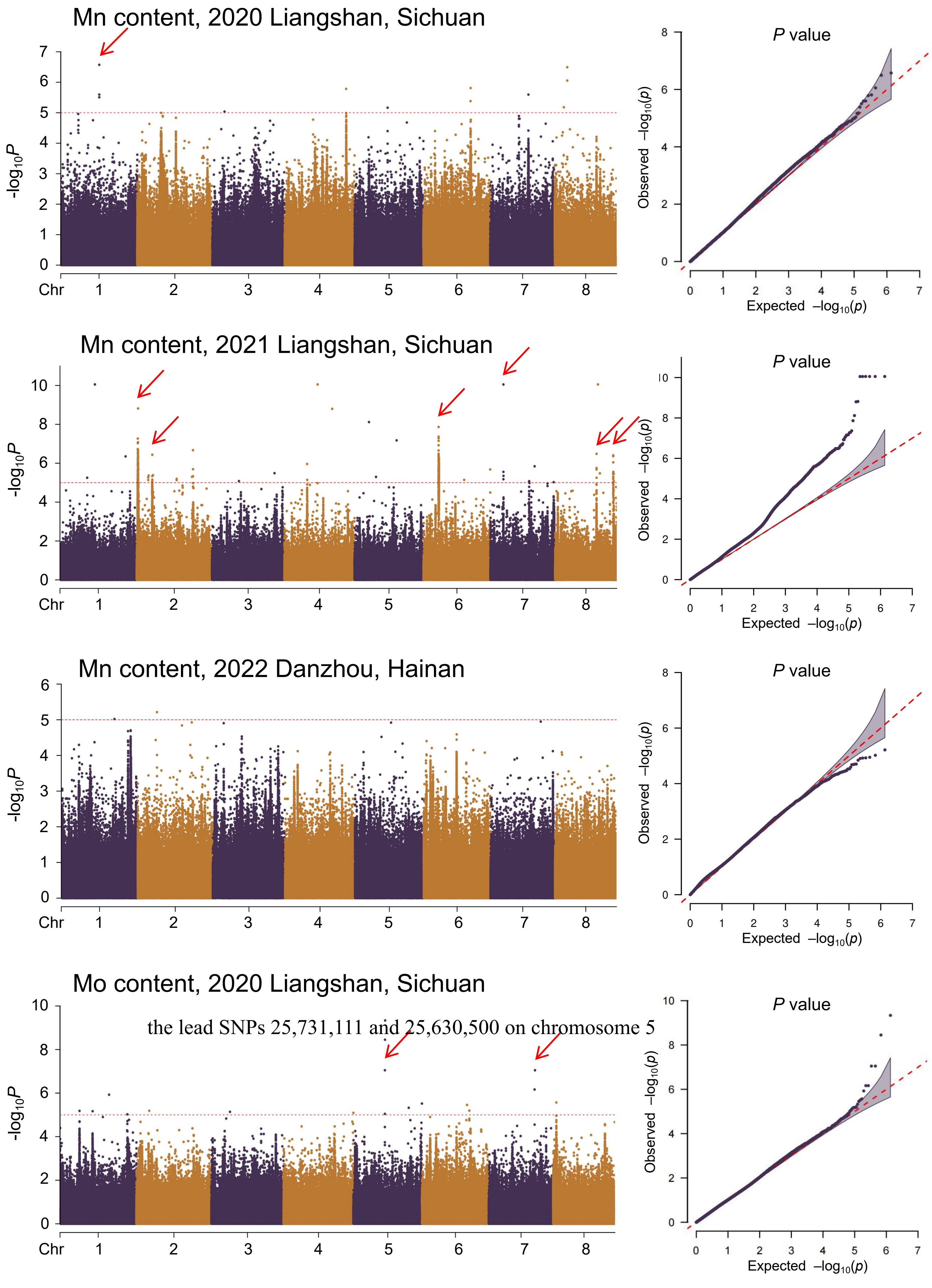

Mn content, 2020 Liangshan, Sichuan
-log10P
Chr
P value
Observed −log10(p)
Expected −log10(p)
Mn content, 2021 Liangshan, Sichuan
-log10P
Chr
P value
Observed −log10(p)
Expected −log10(p)
Mn content, 2022 Danzhou, Hainan
-log10P
Chr
P value
Observed −log10(p)
Expected −log10(p)
Mo content, 2020 Liangshan, Sichuan
the lead SNPs 25,731,111 and 25,630,500 on chromosome 5
-log10P
Chr
P value
Observed −log10(p)
Expected −log10(p)

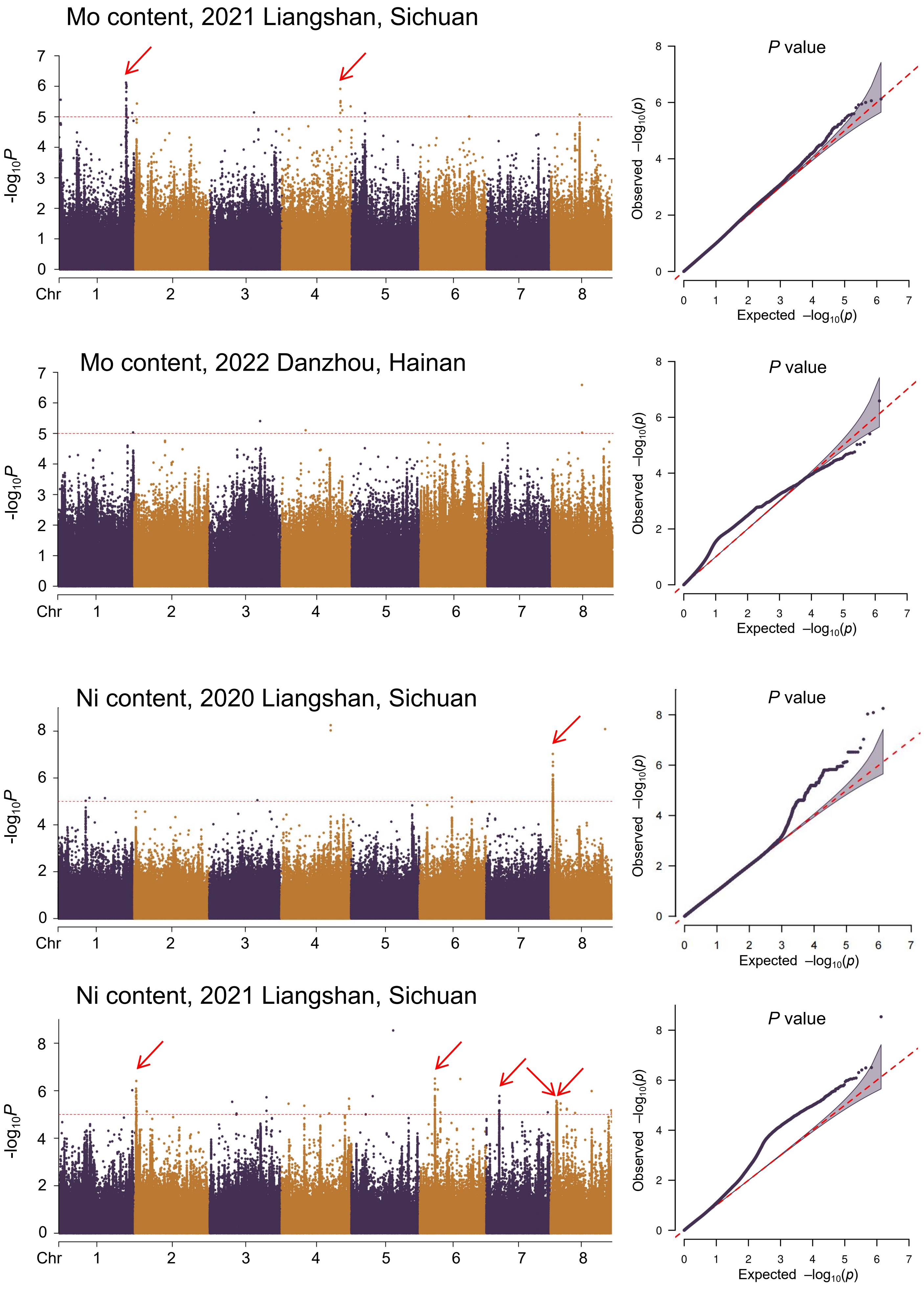

Mo content, 2021 Liangshan, Sichuan
-log10P
Chr
P value
Observed −log10(p)
Expected −log10(p)
Mo content, 2022 Danzhou, Hainan
-log10P
Chr
P value
Observed −log10(p)
Expected −log10(p)
Ni content, 2020 Liangshan, Sichuan
-log10P
Chr
P value
Observed −log10(p)
Expected −log10(p)
Ni content, 2021 Liangshan, Sichuan
-log10P
Chr
P value
Observed −log10(p)
Expected −log10(p)

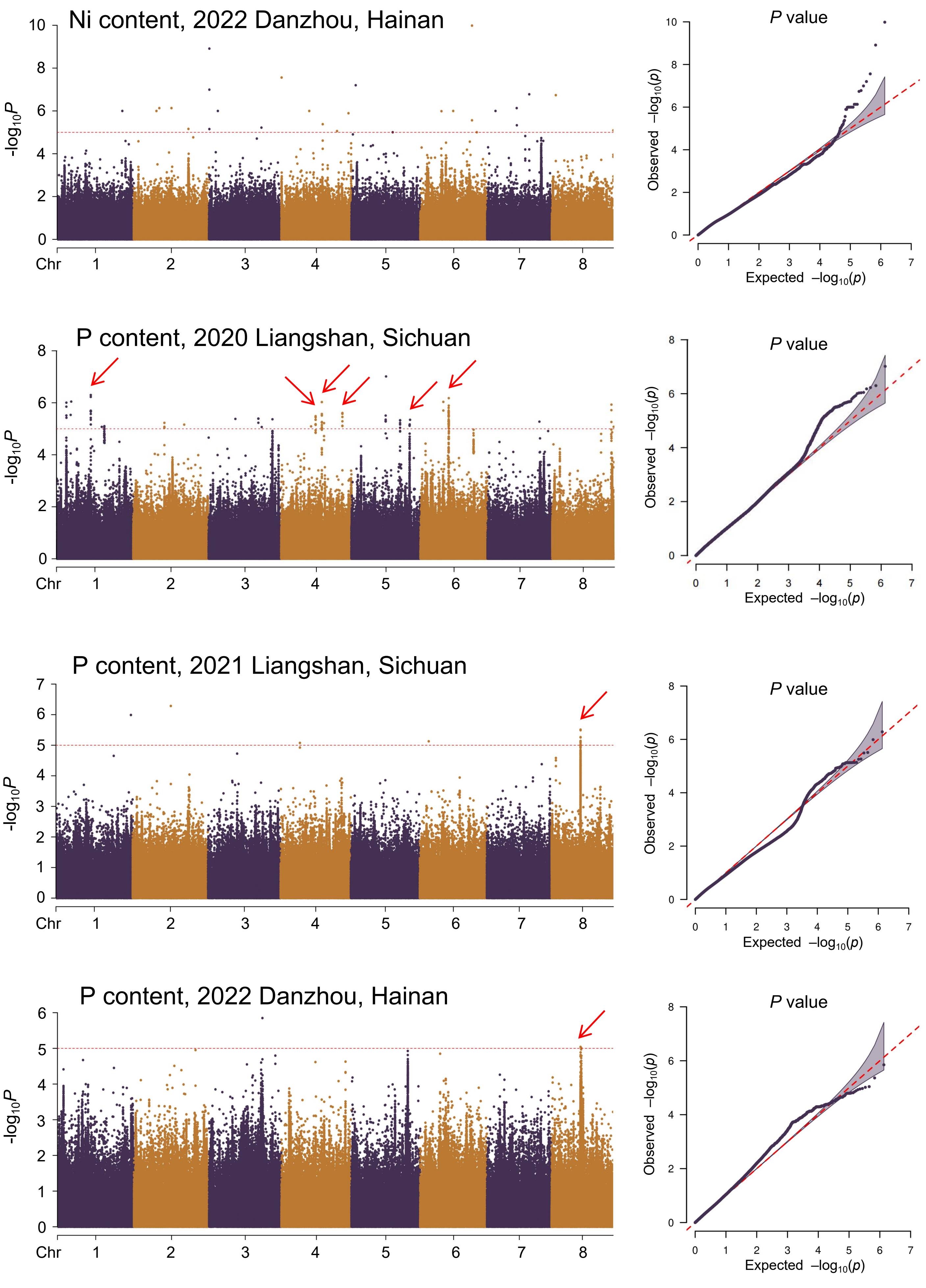

Ni content, 2022 Danzhou, Hainan
P content, 2020 Liangshan, Sichuan
P content, 2021 Liangshan, Sichuan
P content, 2022 Danzhou, Hainan
-log10P
Chr
P value
Observed −log10(p)
Expected −log10(p)

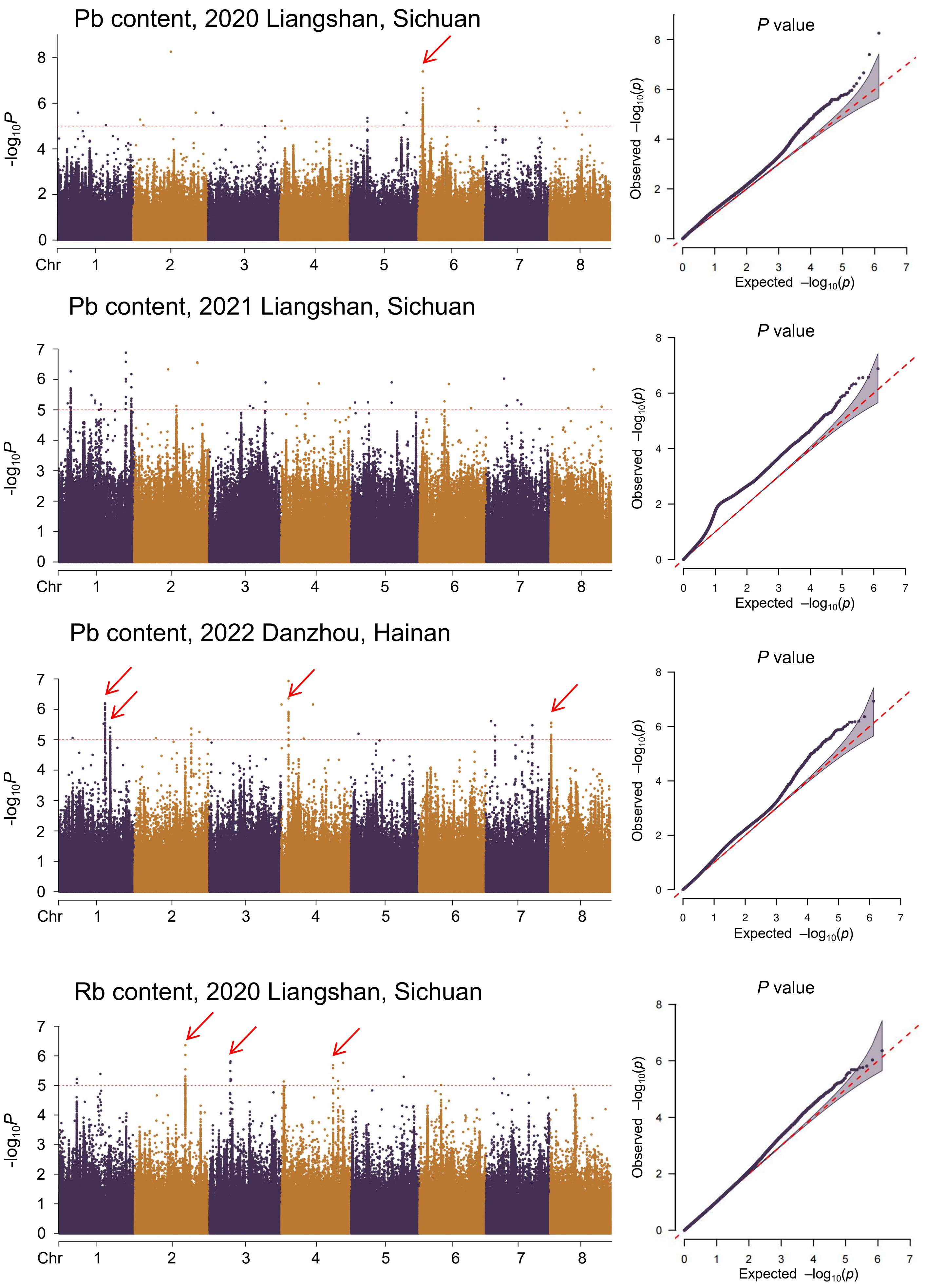

Pb content, 2020 Liangshan, Sichuan
Pb content, 2021 Liangshan, Sichuan
Pb content, 2022 Danzhou, Hainan
Rb content, 2020 Liangshan, Sichuan
$-\log_{10}P$
Chr
1 2 3 4 5 6 7 8
P value
Observed $-\log_{10}(p)$
Expected $-\log_{10}(p)$

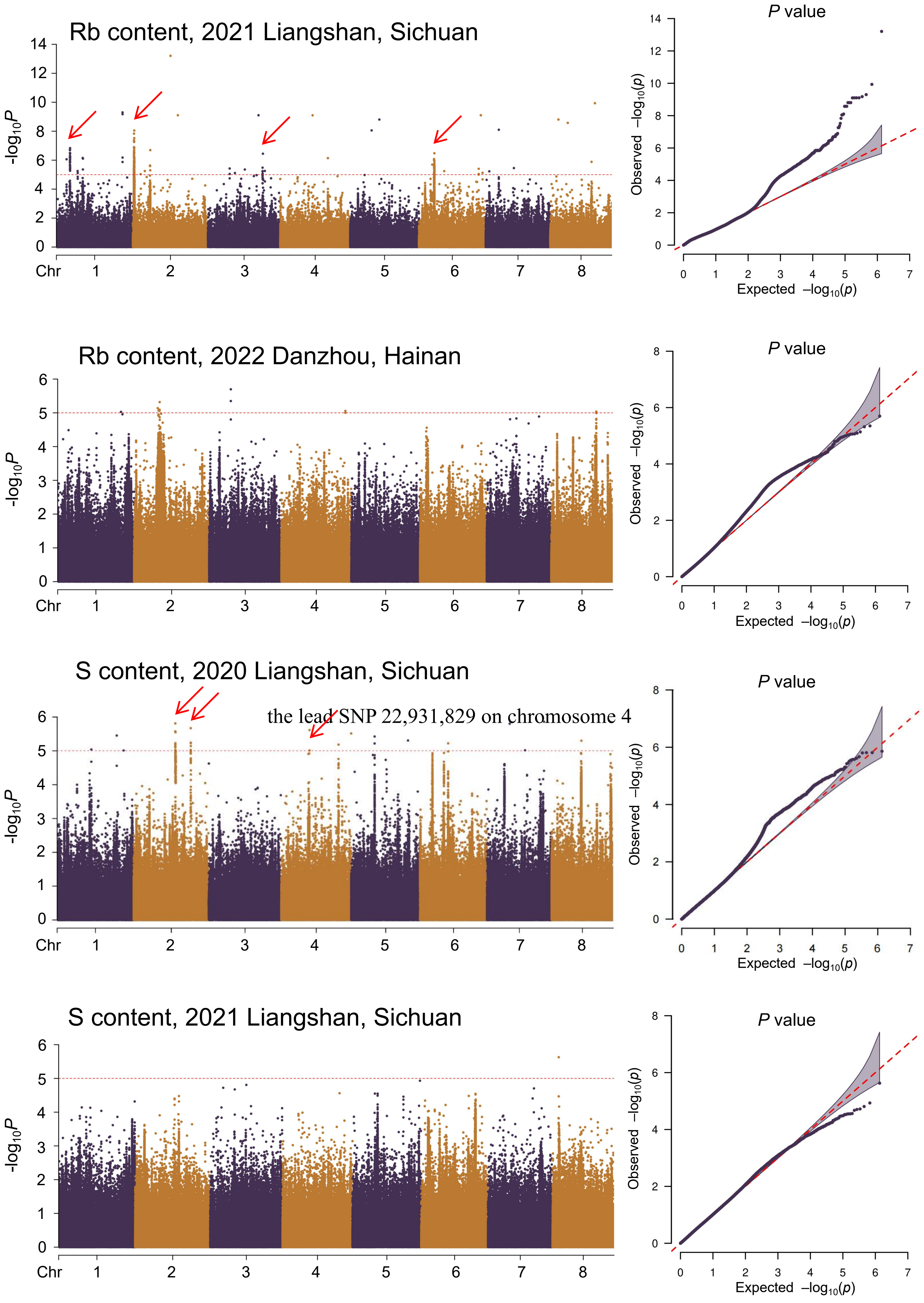

Rb content, 2021 Liangshan, Sichuan
Rb content, 2022 Danzhou, Hainan
S content, 2020 Liangshan, Sichuan
the lead SNP 22,931,829 on chromosome 4
S content, 2021 Liangshan, Sichuan
-log10P
Chr
1
2
3
4
5
6
7
8
P value
Observed –log10(p)
Expected –log10(p)

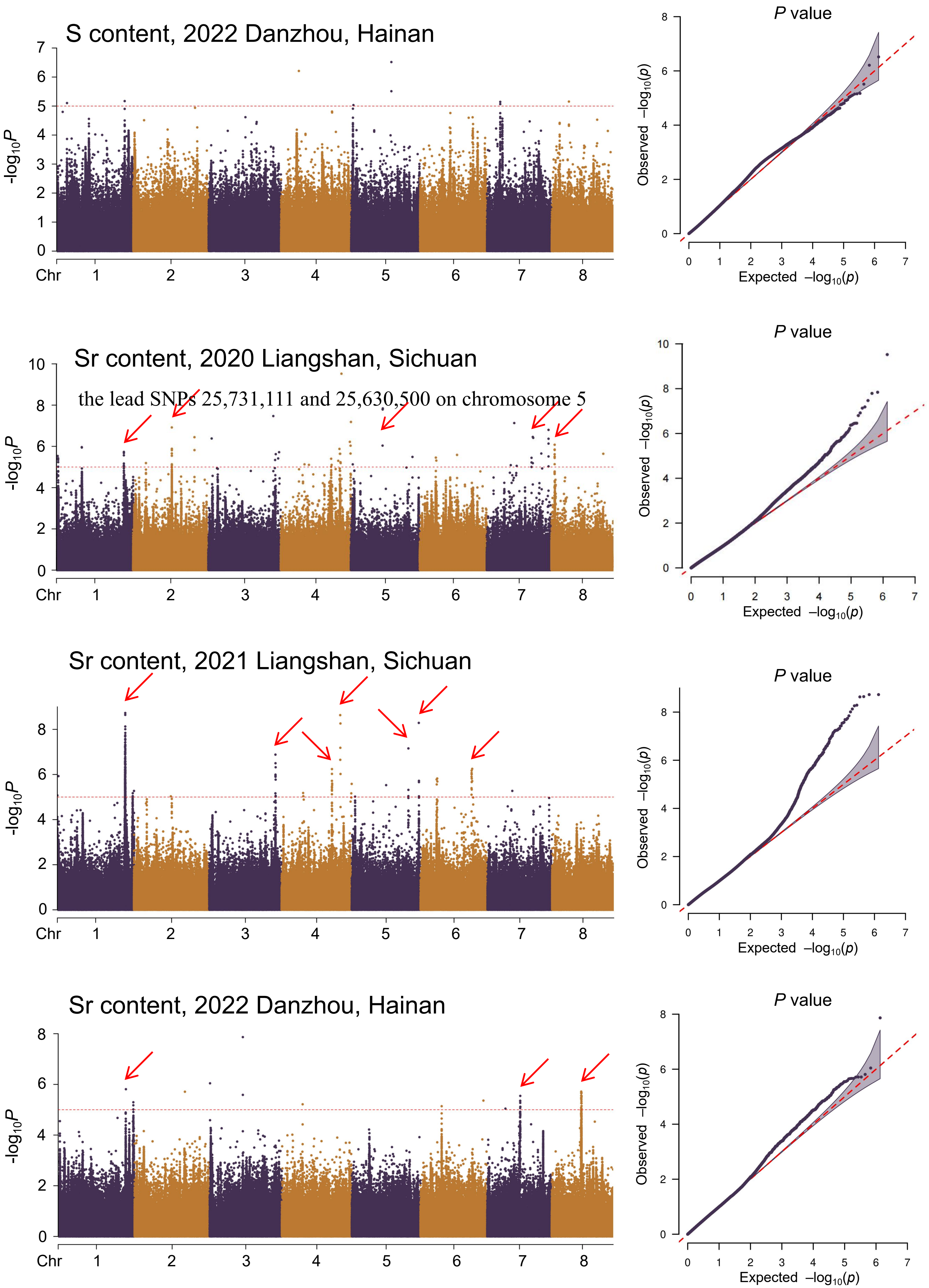
S content, 2022 Danzhou, Hainan
Sr content, 2020 Liangshan, Sichuan
the lead SNPs 25,731,111 and 25,630,500 on chromosome 5
Sr content, 2021 Liangshan, Sichuan
Sr content, 2022 Danzhou, Hainan
-log10P
Chr
P value
Observed −log10(p)
Expected −log10(p)

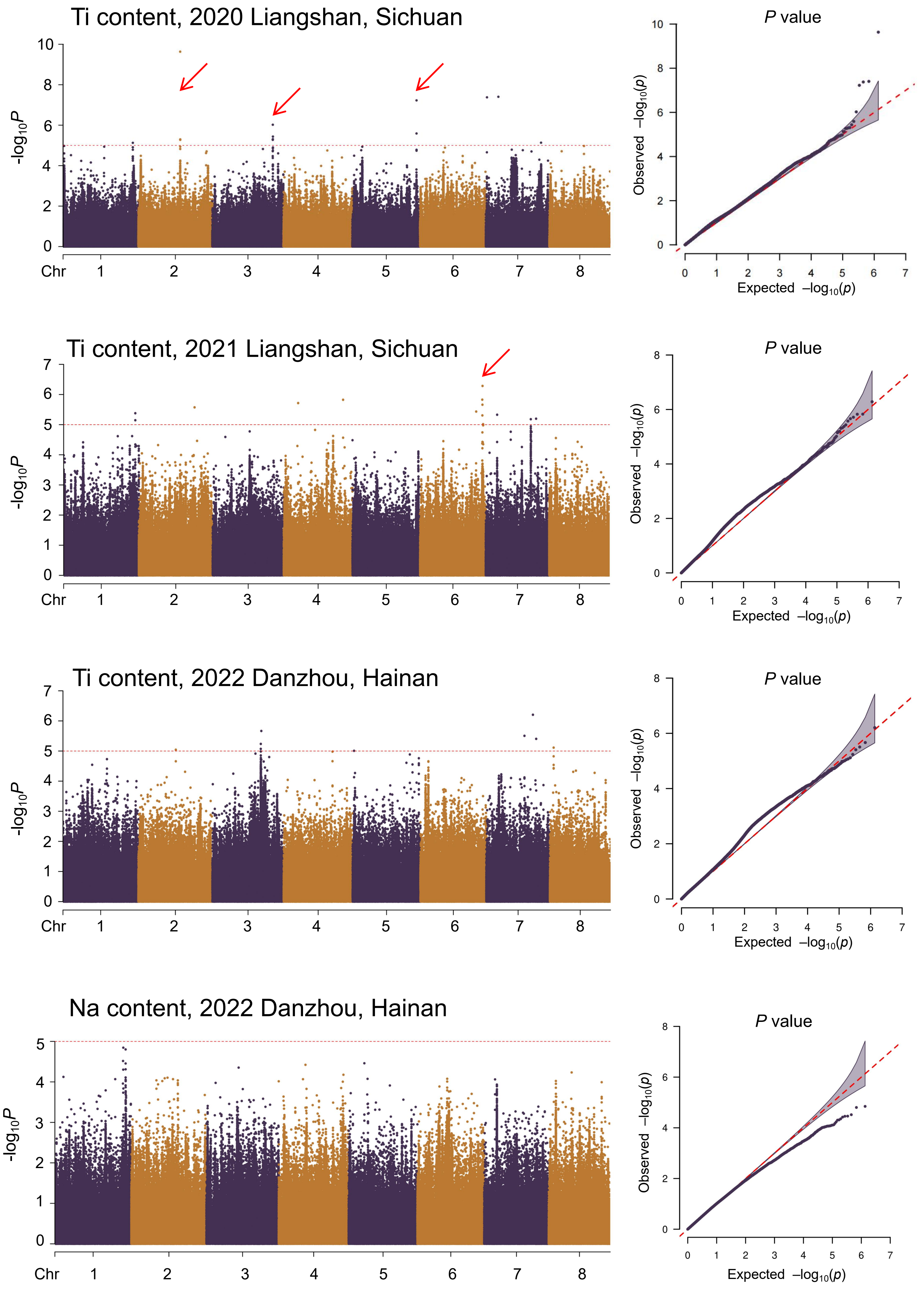
Ti content, 2020 Liangshan, Sichuan
-log10P
Chr 1 2 3 4 5 6 7 8
P value
Observed –log10(p)
Expected –log10(p)
Ti content, 2021 Liangshan, Sichuan
-log10P
Chr 1 2 3 4 5 6 7 8
P value
Observed –log10(p)
Expected –log10(p)
Ti content, 2022 Danzhou, Hainan
-log10P
Chr 1 2 3 4 5 6 7 8
P value
Observed –log10(p)
Expected –log10(p)
Na content, 2022 Danzhou, Hainan
-log10P
Chr 1 2 3 4 5 6 7 8
P value
Observed –log10(p)
Expected –log10(p)

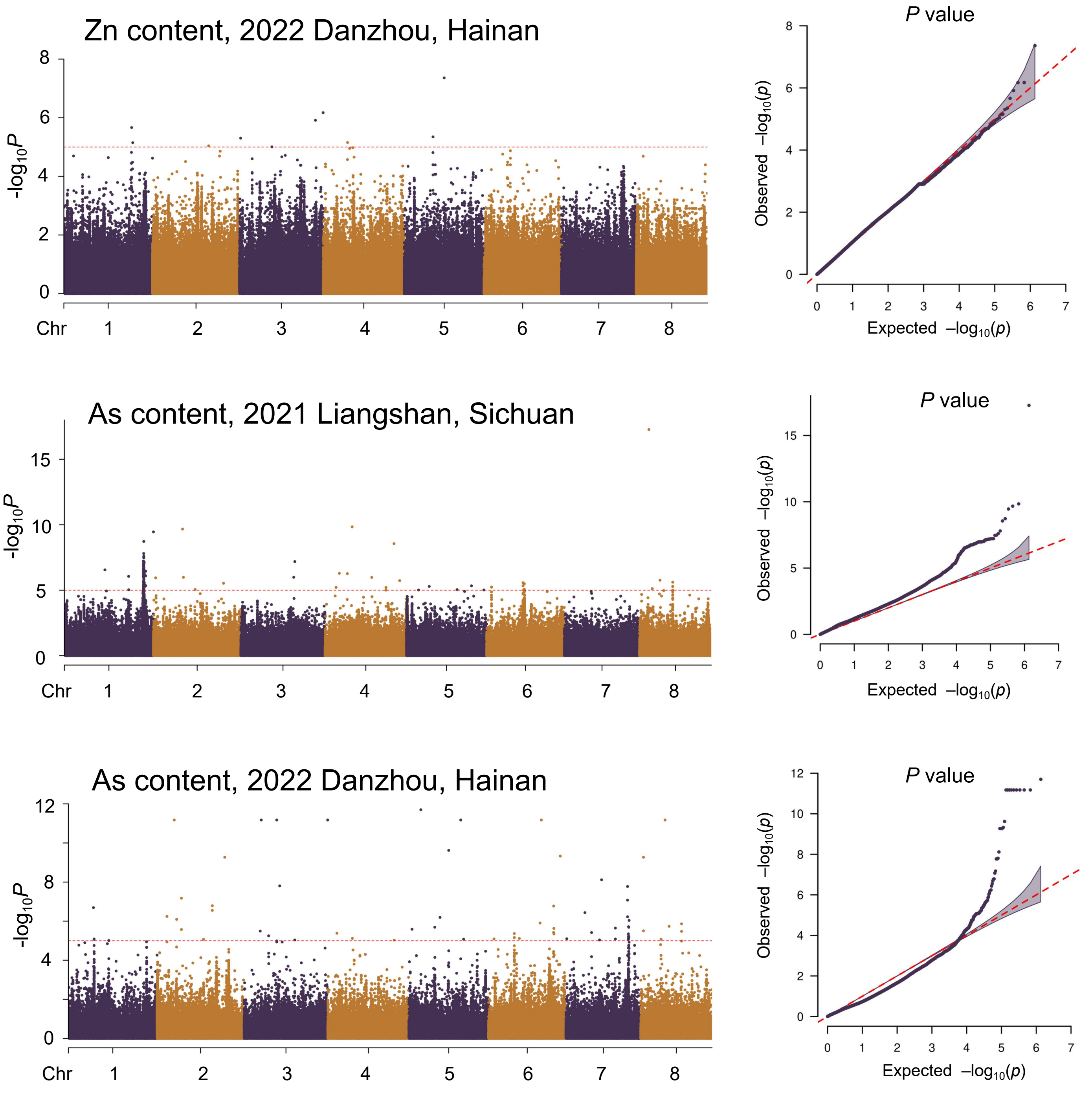

Figure S4. Manhattan and QQ plots of GWAS results of 20 ion elements.

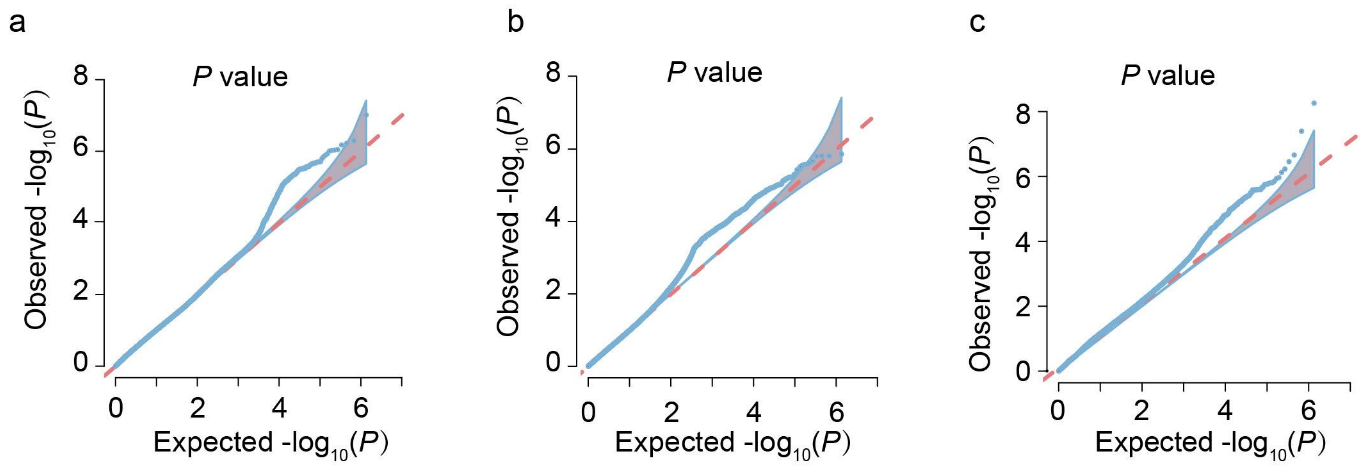

Figure S5. QQ plots of GWAS results of P (a), S (b) and Pb (c) ion elements.

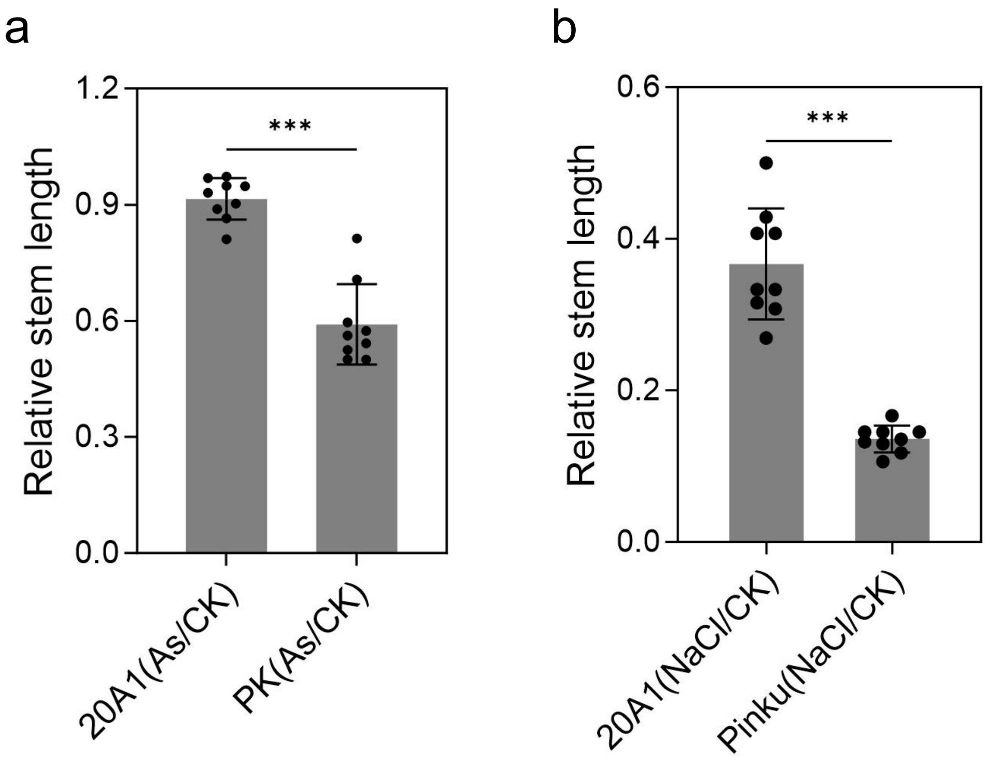

Figure S6. a. The relative stem length of 20A1 and Pinku under control and 0.6 μg/ml As conditions. n=9. b. The relative stem length of 20A1 and Pinku under control and 150 mM NaCl conditions. n=9. Data is presented as mean ± SD. Significant differences are indicated by asterisks: *** $P < 0.001$, two-tailed t-test.

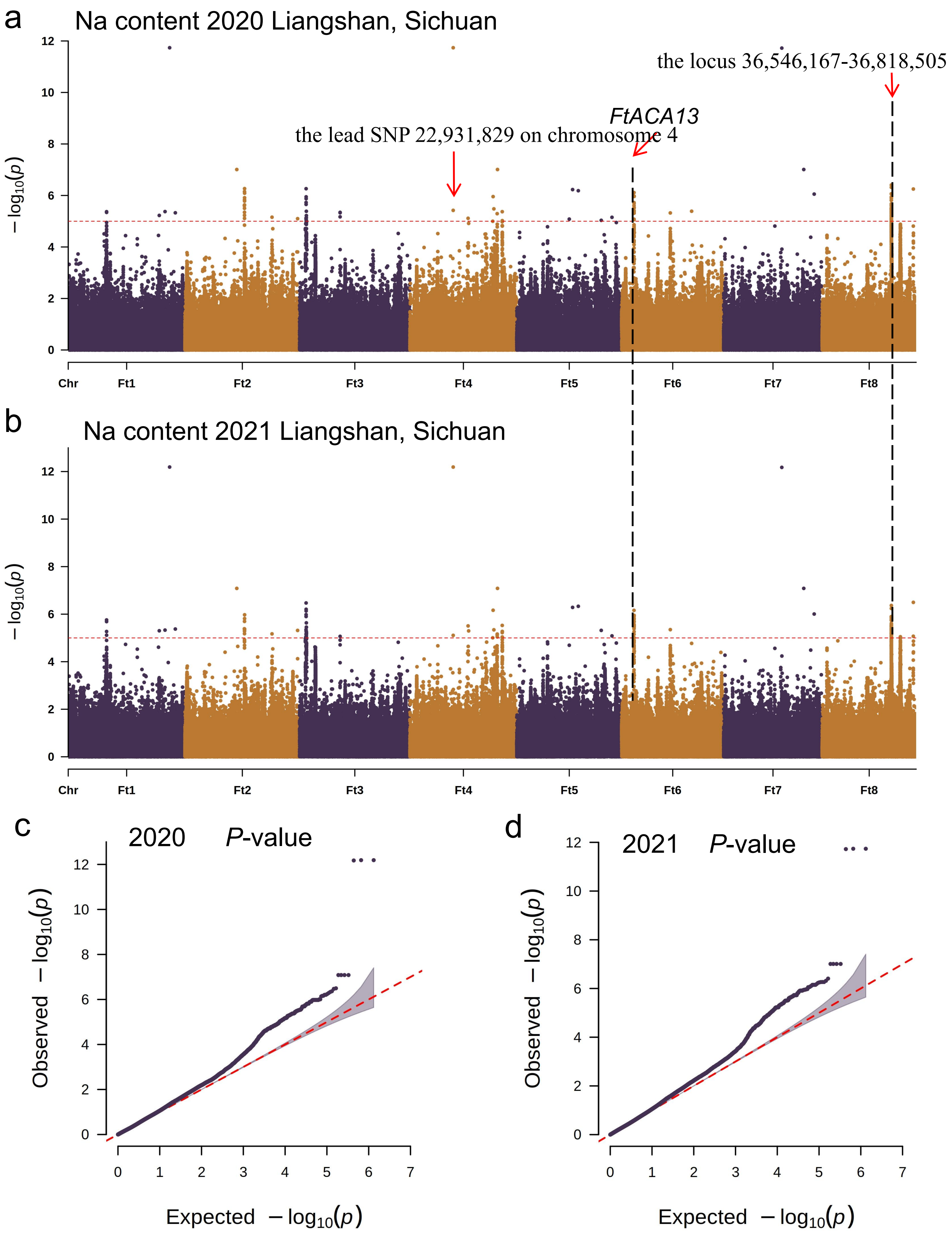

Figure S7. Manhattan and QQ plots of GWAS results of Na content of Tartary buckwheat.

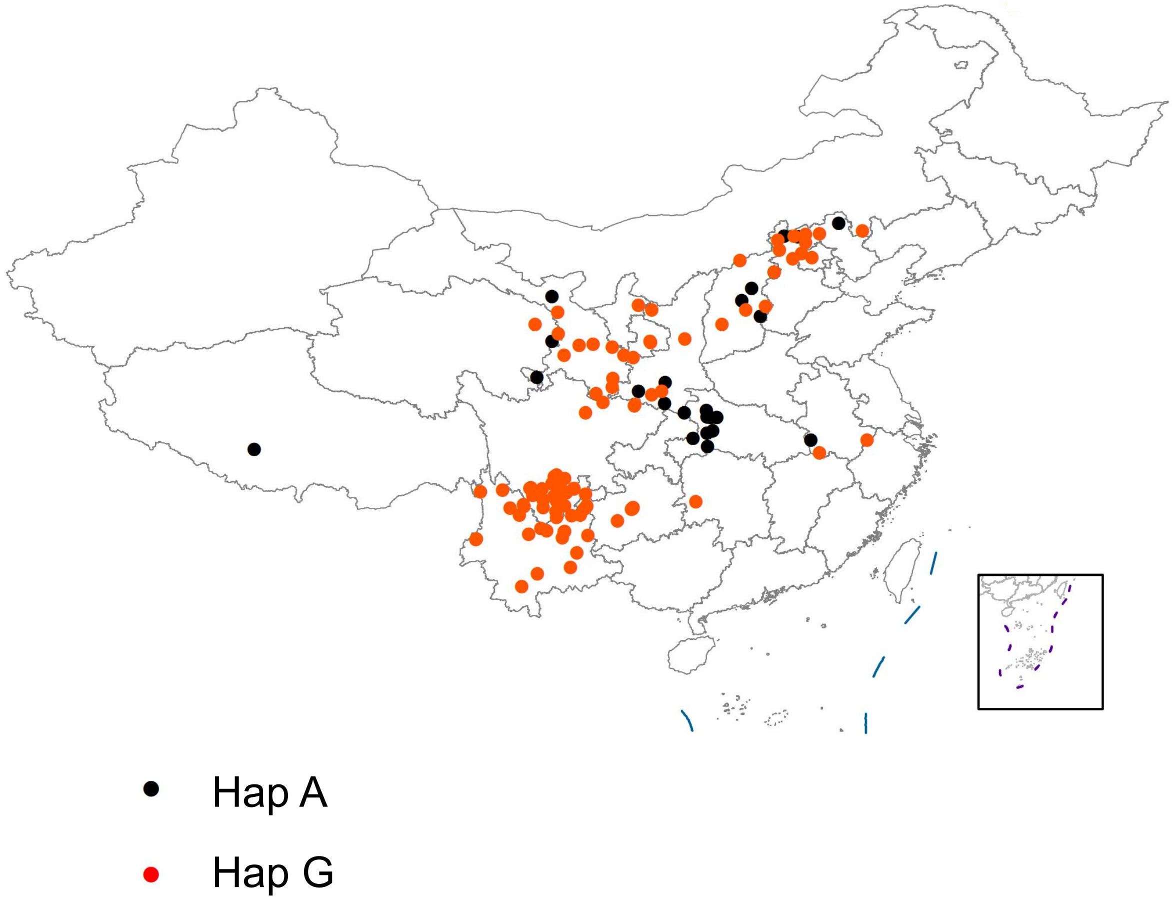

Figure S8. Geographic distribution of the Hap G (red) and Hap A (black) located in the promoter of *FtACA13* in Tartary buckwheat accessions.

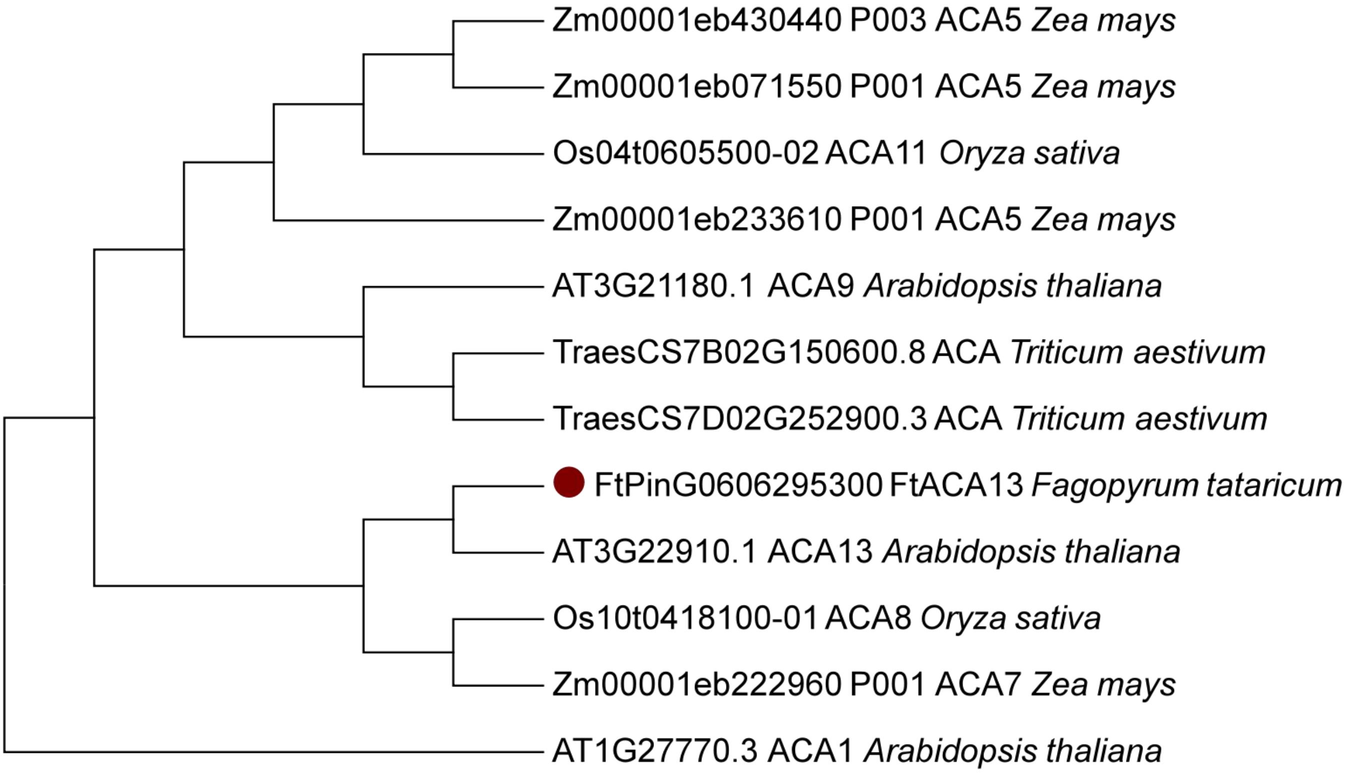

Figure S9. FtACA13 phylogenetic tree based on the neighbor joining method tree using full-length amino acid sequences of orthologues genes in Tartary buckwheat and other plants.

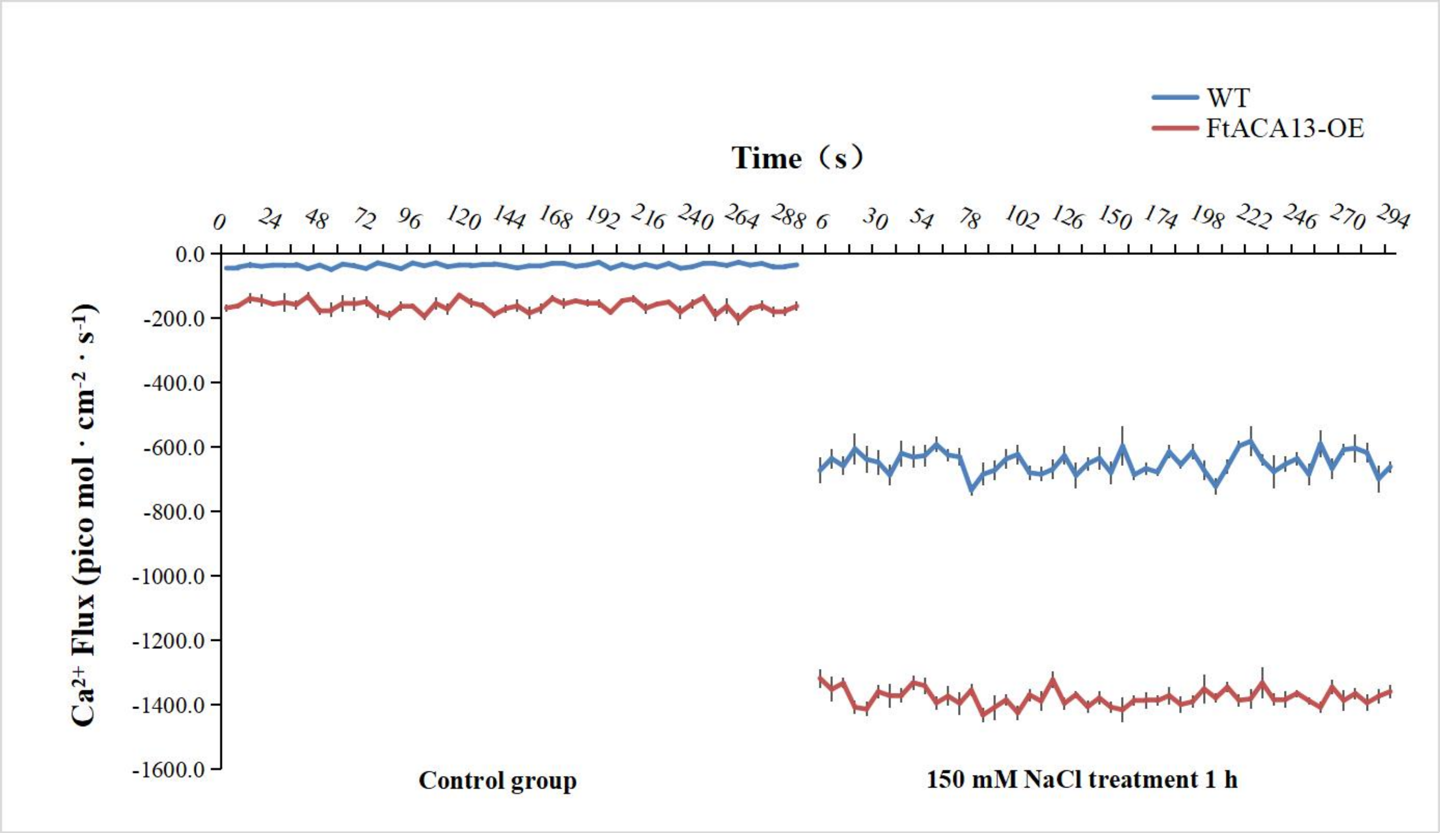

Figure S10. Comparison of extracellular calcium flux in living roots of *FtACA13* overexpression hairy roots and wild type. The control group indicates no treatment. The error bars indicate mean ± SD from six biological replicates.

a

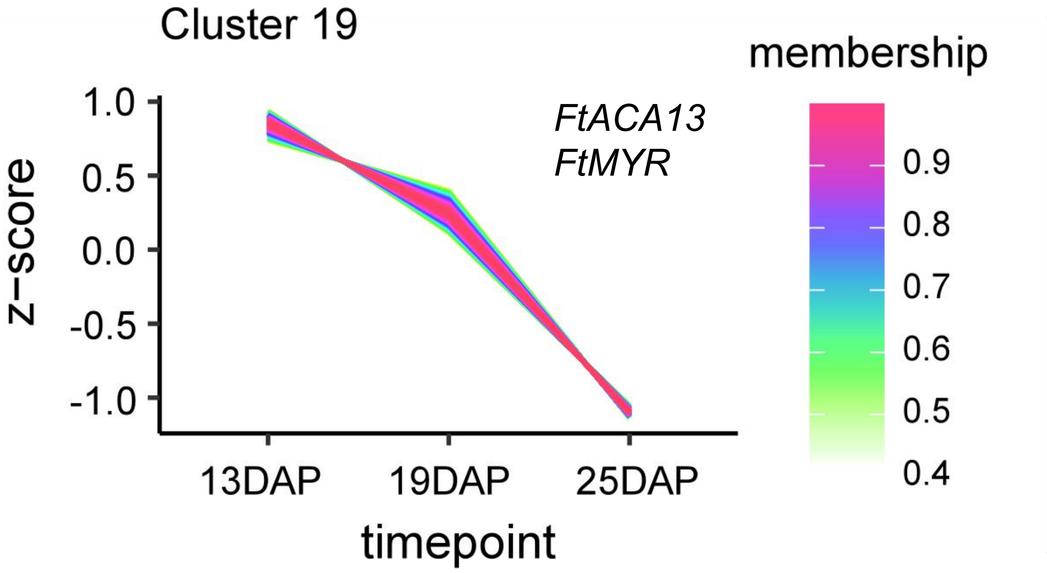

b

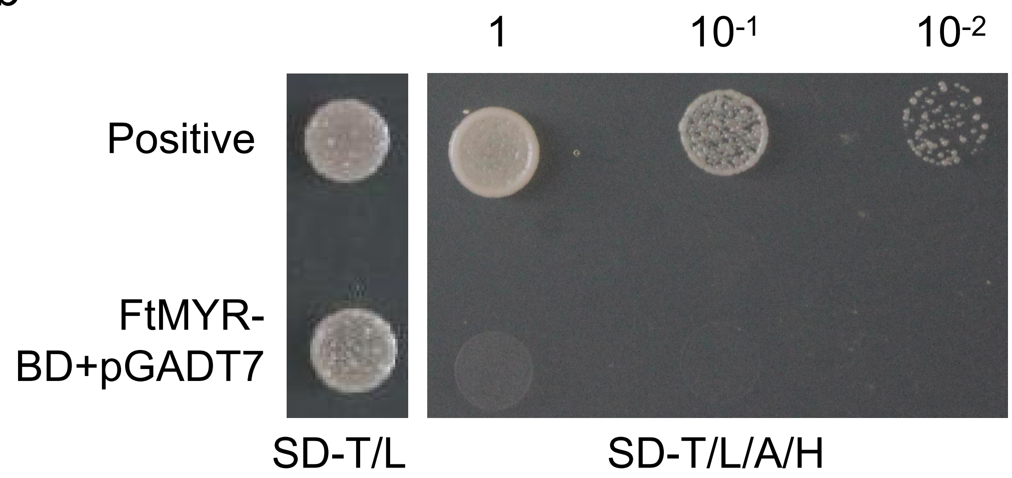

Figure S11. a. Analysis of the co-expression patterns of *FtACA13* and *FtMYR* transcription factors in the transcriptome of buckwheat seed development. b. Transcriptional activation assay of FtMYR protein in yeast. pBD-53 was used as a positive control.

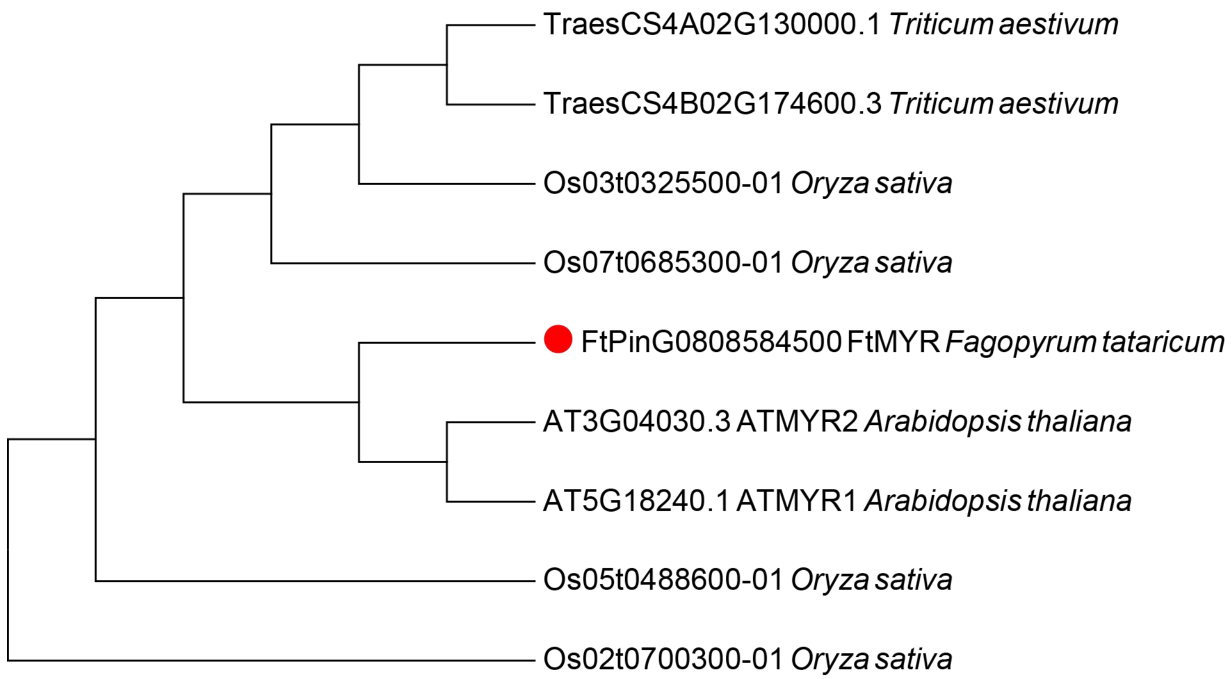

Figure S12. FtMYR phylogenetic tree based on the neighbor joining method tree using full-length amino acid sequences of orthologues genes in Tartary buckwheat and other plants.

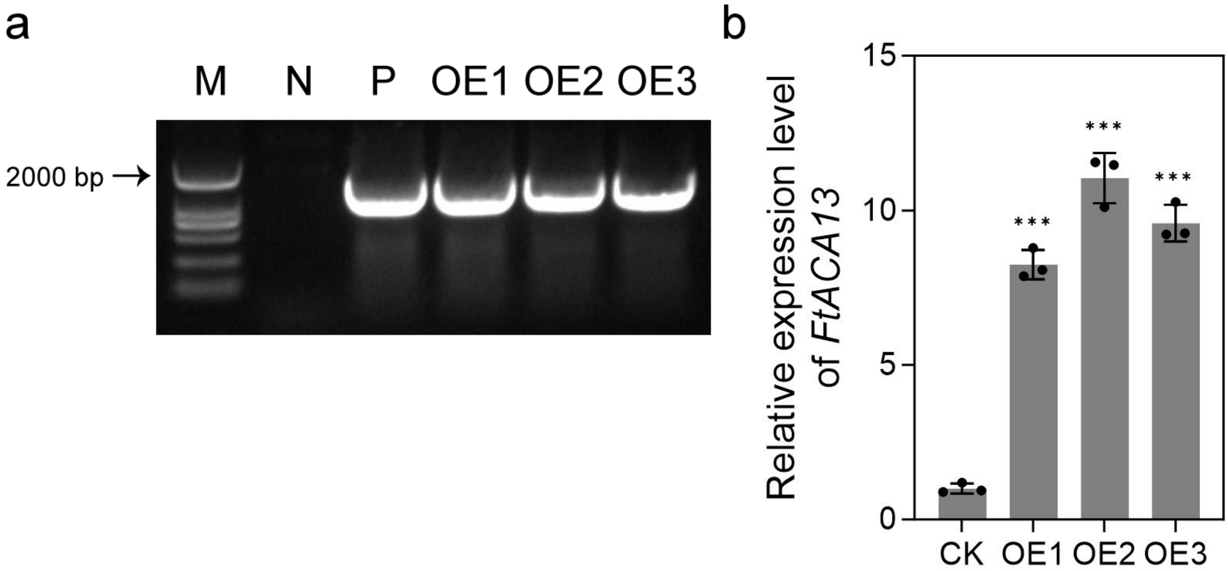

Figure S13. PCR (a) and RT-qPCR (b) analysis of hairy root lines expressing *FtACA13*. The hairy roots from wild type were used as negative control. N: negative control; P: positive control; M: DNA marker. The error bars indicate mean ± SD from three biological replicates. *** $P < 0.001$, two-tailed *t*-test.

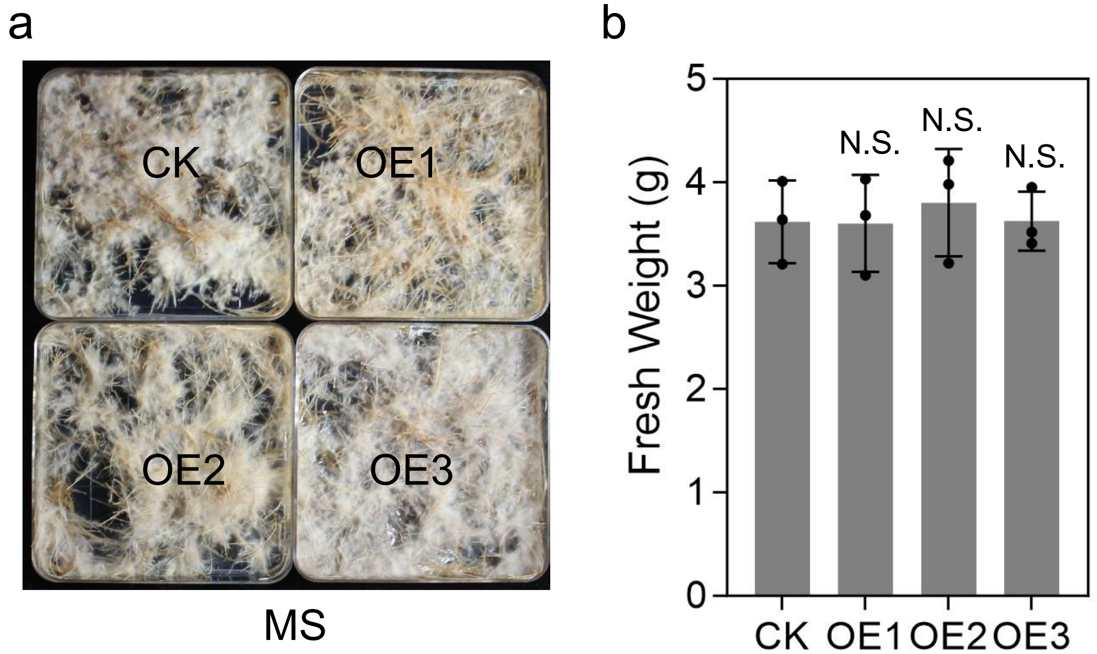

Figure S14. a. The hairy root phenotype grown on MS medium for 3 weeks. b. The fresh weight of different genotype hairy roots. The error bars indicate mean ± SD from three biological replicates. N.S. no significance, two-tailed *t*-test.

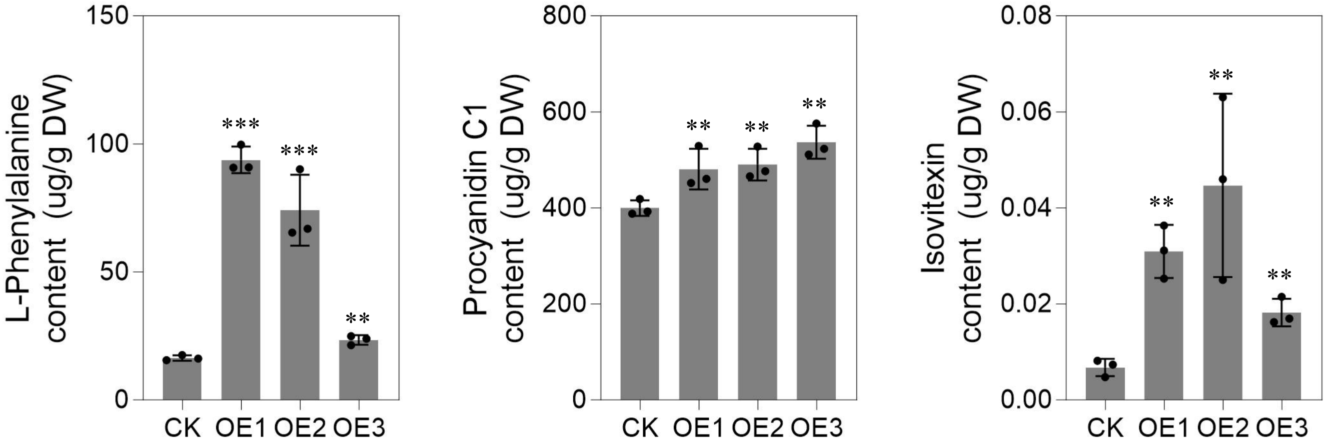

Figure S15. L-phenylalanine, procyanidin, and isovitexin contents in different genotype hairy roots. The error bars indicate mean ± SD from three biological replicates. Asterisk (***) and (**) indicate significant difference at P < 0.001 and P < 0.01 using two-tailed t-test.

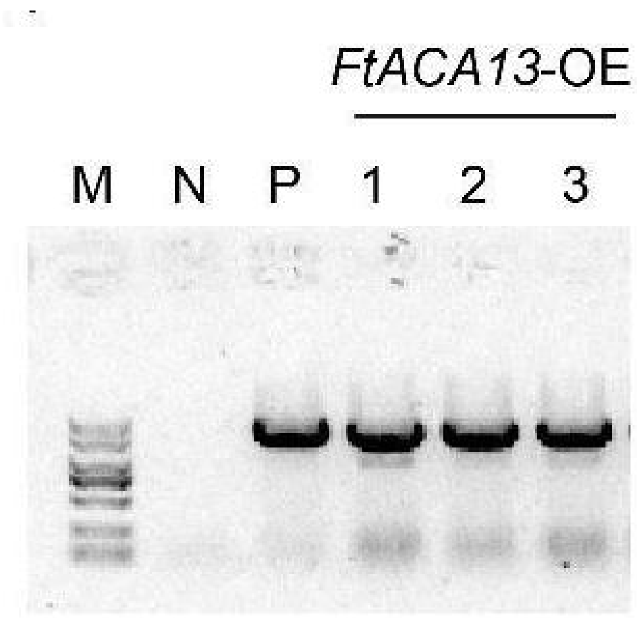

Figure S16. PCR analysis of *Arabidopsis* lines heterologously expressing *FtACA13*. The leaves from wild type (Col-0) were used as negative control. N: negative control; P: positive control; M: DNA marker.

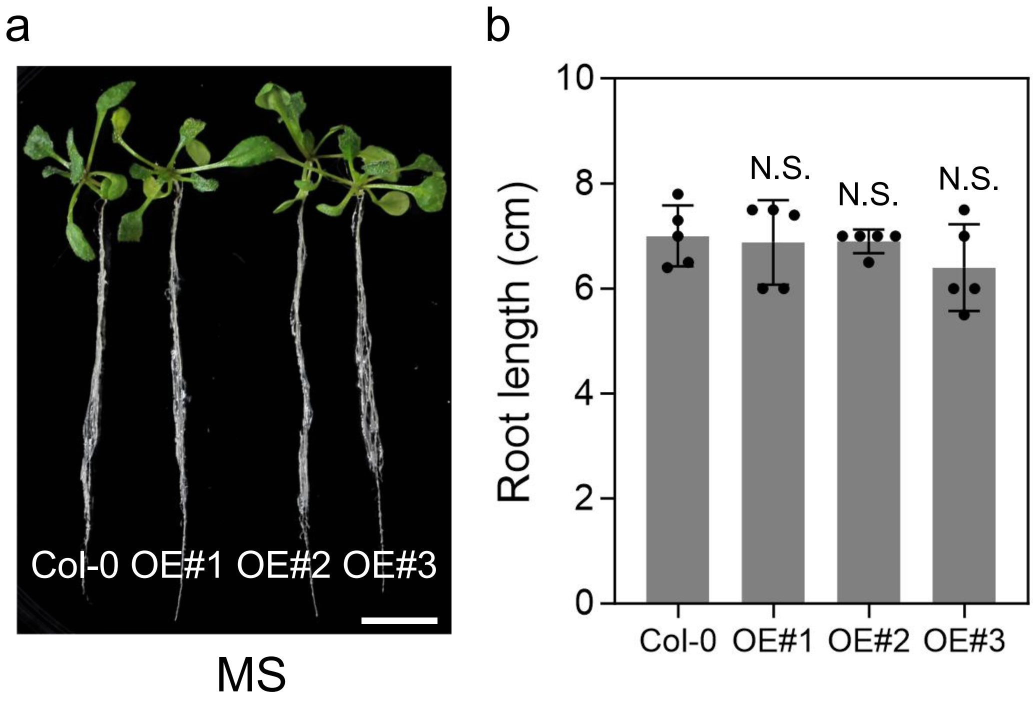

Figure S17. a. The phenotype of different genotype *Arabidopsis* lines grown on MS medium for 3 weeks. b. The root length of *Arabidopsis* lines. The error bars indicate mean ± SD from five biological replicates. N.S. no significance, two-tailed *t*-test.

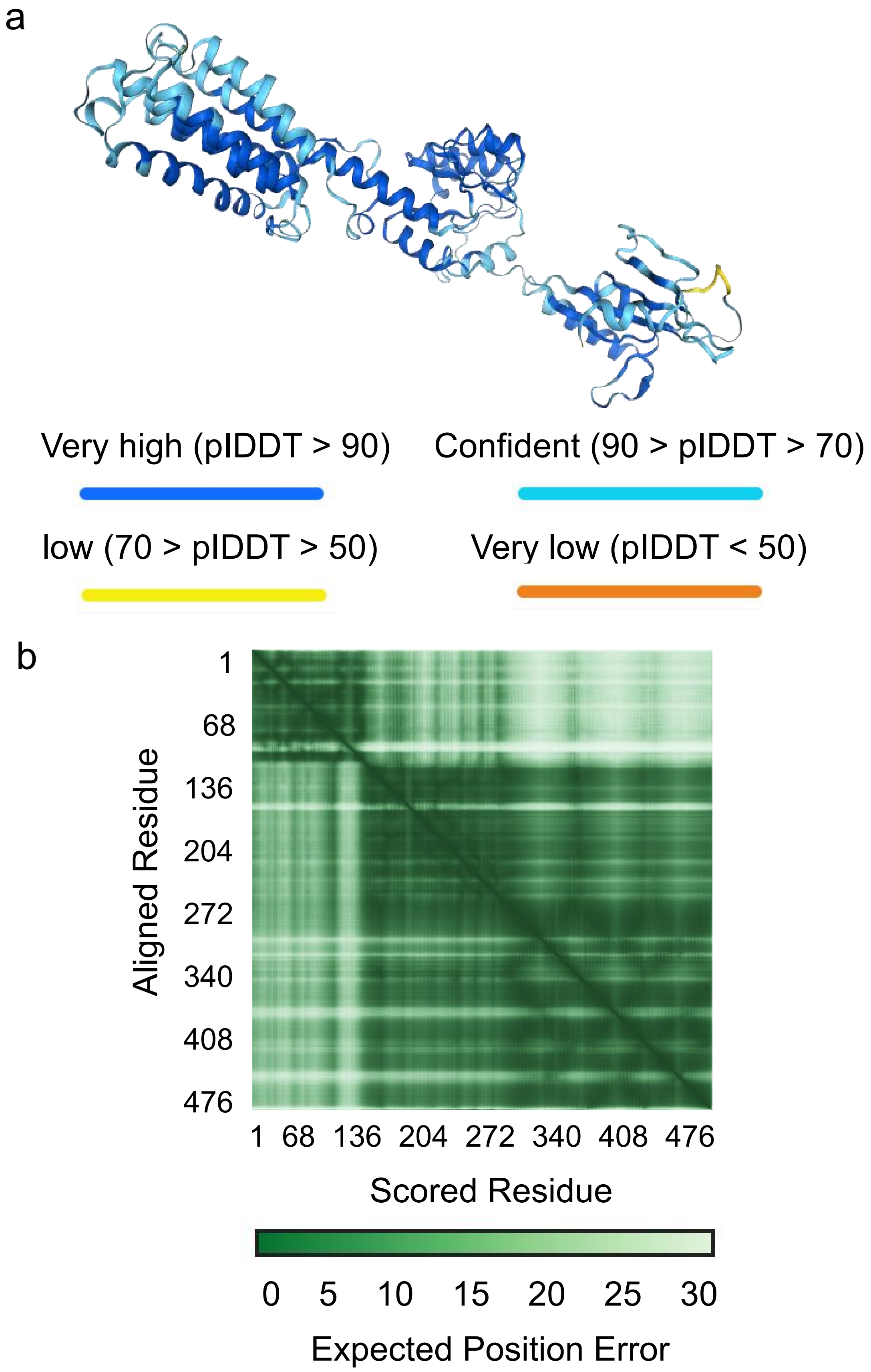

Figure S18. a. Predicted structure of FtACA13 coloured by pLDDT (orange: 0-50, yellow: 50-70, cyan 70-90, and blue 90-100). b. Predicted Aligned Error (PAE) matrix of same prediction (darker is more confident).

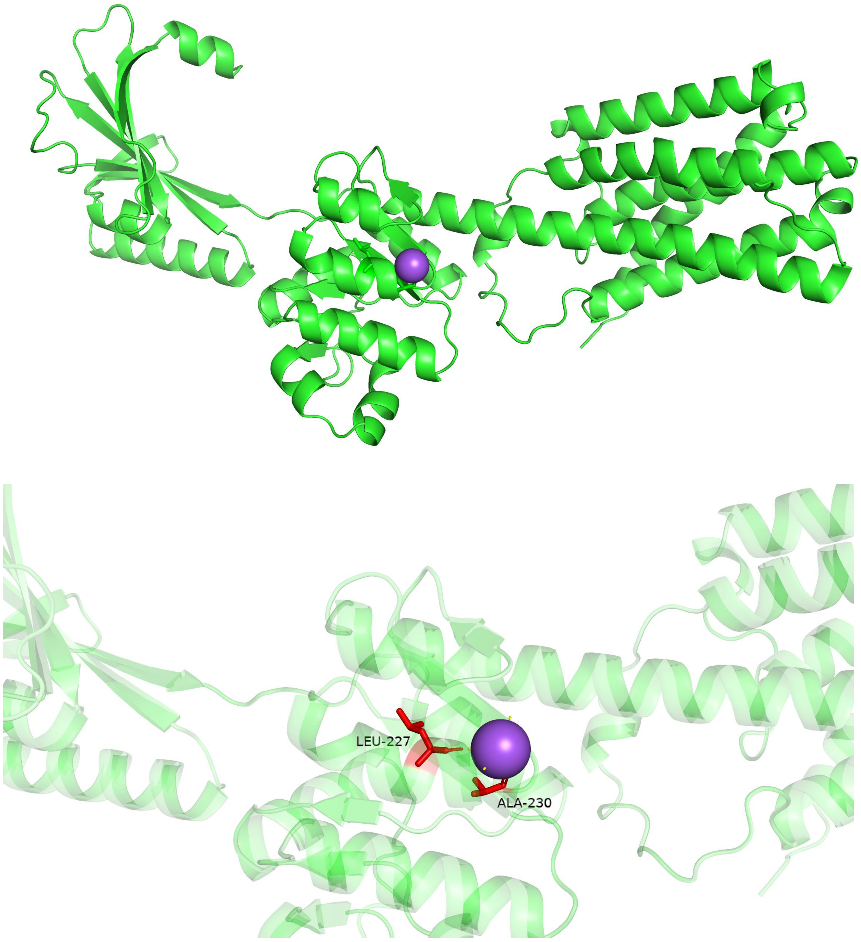

Figure S19. AlphaFold 3 predictions of FtACA13 protein interaction with sodium ion.

```
                          *        20         *        40         *
ACA13_pro_20A1  : TTTAGCTTAATATGACATCATGCGAGCGAAAGTTATTTCAAAAAATAATA :  50
ACA13_pro_Pinku : TTTAGCTTAATATGACATCATGCGAGCGAAAGTTATTC--AAAAATAATA :  48
                  TTTAGCTTAATATGACATCATGCGAGCGAAAGTTATT   AAAAATAATA

                        60         *        80         *       100
ACA13_pro_20A1  : TGGCTAATTCATGTTGAATGATTTTGATATCGTATACAGCCTCAAGCTTA : 100
ACA13_pro_Pinku : TGGCTAATTCATGTTGAATGATTTTGATATCGTATACAGACTCAAGCTTA :  98
                  TGGCTAATTCATGTTGAATGATTTTGATATCGTATACAG CTCAAGCTTA

                          *       120         *       140         *
ACA13_pro_20A1  : AAGAGTTTTGTTTGGAAAAAGTTTTGTTTTCATAAGTTTGTACAATTGCA : 150
ACA13_pro_Pinku : AAGAGTTTTG-TTGGAAAAAGTTTTGTTTTCATAAGTTTGTACAATTGCA : 147
                  AAGAGTTTTG TTGGAAAAAGTTTTGTTTTCATAAGTTTGTACAATTGCA

                        160         *       180         *       200
ACA13_pro_20A1  : CACTTTTGCCTTTATTTATTACTCTTTCAGTTTCTCCATAGATATTTTTA : 200
ACA13_pro_Pinku : CACTTTTGCCTTTATTTATTACTCTTTCAGTTTCTTCATAGATATTTTTA : 197
                  CACTTTTGCCTTTATTTATTACTCTTTCAGTTTCT CATAGATATTTTTA

                          *       220         *       240         *
ACA13_pro_20A1  : GAGTAAAAAATACACATCTTAAGAAATTTAAATATAGAAAAATATAATAT : 250
ACA13_pro_Pinku : GAGTAAAAAATACACATATTAAGAAATTTAAA-GTAAAAAAATATAATAT : 246
                  GAGTAAAAAATACACAT TTAAGAAATTTAAA  TA AAAAATATAATAT

                        260         *       280         *       300
ACA13_pro_20A1  : TTAATGATGTATAATAATTTTAAAAAGTGTAAAAAAAATGGTAGAGTGTC : 300
ACA13_pro_Pinku : TTAATGATGCATAATAA--------------------------------- : 263
                  TTAATGATG ATAATAA

                          *       320         *       340         *
ACA13_pro_20A1  : GAAAATTGTATGAGAAAATGAAATATTGCTTTGAAAATACAAACAATAAA : 350
ACA13_pro_Pinku : -------------------------------------------------- :   -

                        360         *       380         *       400
ACA13_pro_20A1  : TAAAAAGGTAGTTACTATTATATTTATAGAAAGTTTGAGTATTAATAACA : 400
ACA13_pro_Pinku : -------------------------------------------------A : 264
                                                                   A

                          *       420         *       440         *
ACA13_pro_20A1  : CTTATAAGTCTAAATATTATGAAATAAAATTTTAGAGTTCAAATAACAAA : 450
ACA13_pro_Pinku : TCCATAAGGTCAAATATTATGAAAT-AAATTTTAGAGCTCAAATGACAAA : 313
                     ATAAG   AAATATTATGAAAT AAATTTTAGAG TCAAAT ACAAA

                        460         *       480         *       500
ACA13_pro_20A1  : TATTTAAAAACAGATAGAGTAATAAAAAGGGCTTACATCATTTAATAAAA : 500
ACA13_pro_Pinku : TATTTGAAAACAGATGGAGTAATAAAAAGGGCTTACATTATTTAATAAAA : 363
                  TATTT AAAACAGAT GAGTAATAAAAAGGGCTTACAT ATTTAATAAAA
```

Figure S20. SV in the promoter of *FtACA13* between 20A1 and Pinku.

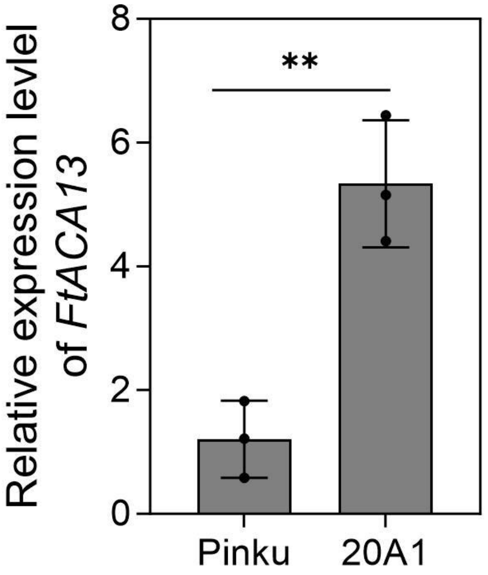

Figure S21. The expression level of *FtACA13* between Pinku and 20A1 Tartary buckwheats. The error bars indicate mean ± SD from three biological replicates. ** *P* < 0.01, two-tailed *t*-test.

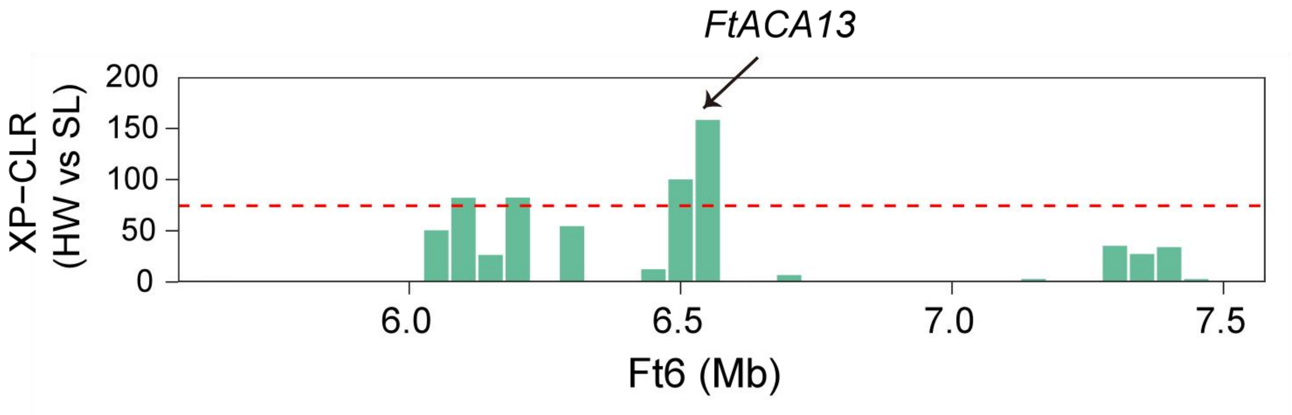

Figure S22. Selective signals identified through comparisons between HW and SL using XP-CLR on the 6 chromosomes. The dashed line represents the top 5% of values therefore scores in these regions were regarded as selective sweeps.

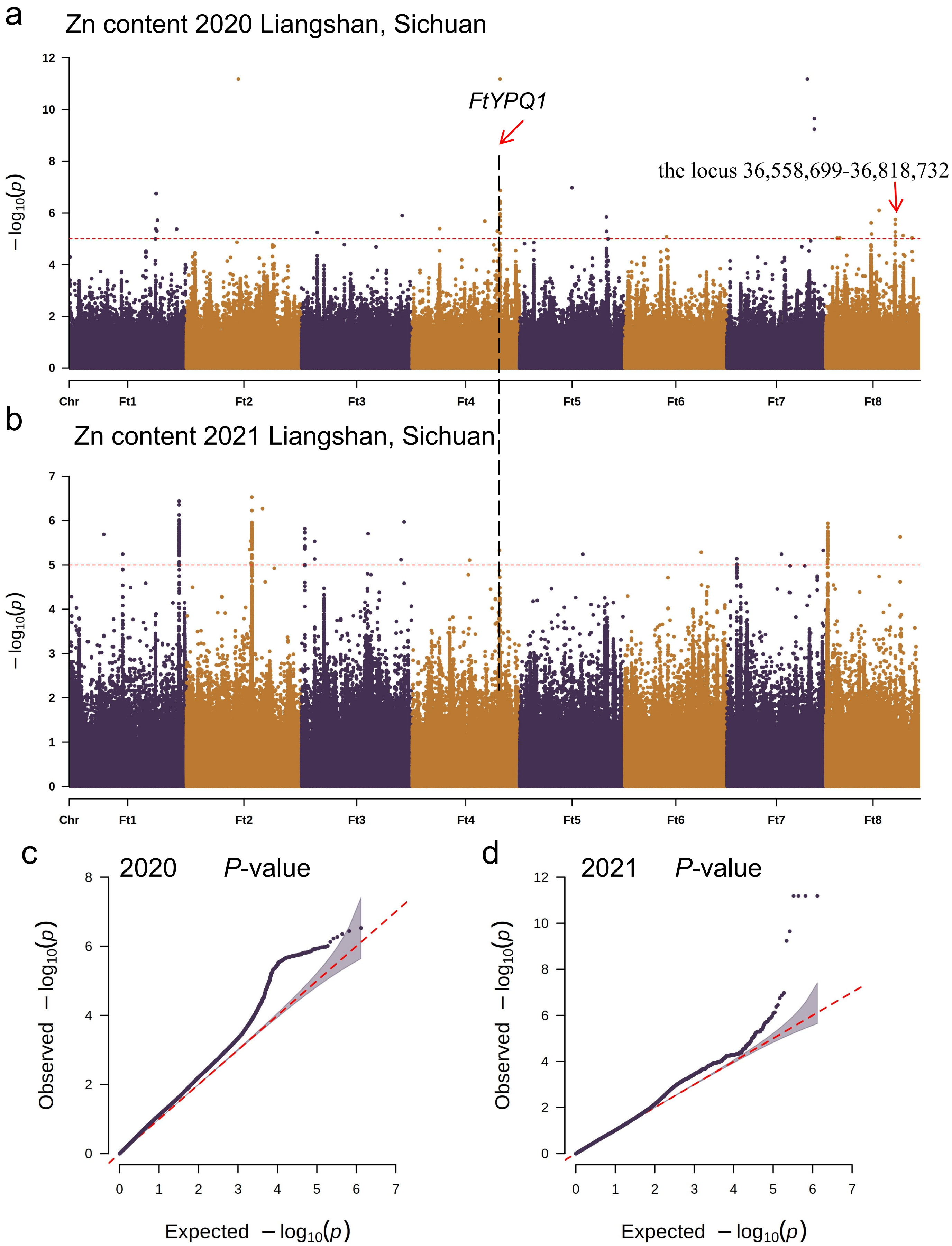

Figure S23. Manhattan and QQ plots of GWAS results of Zn content of Tartary buckwheat.

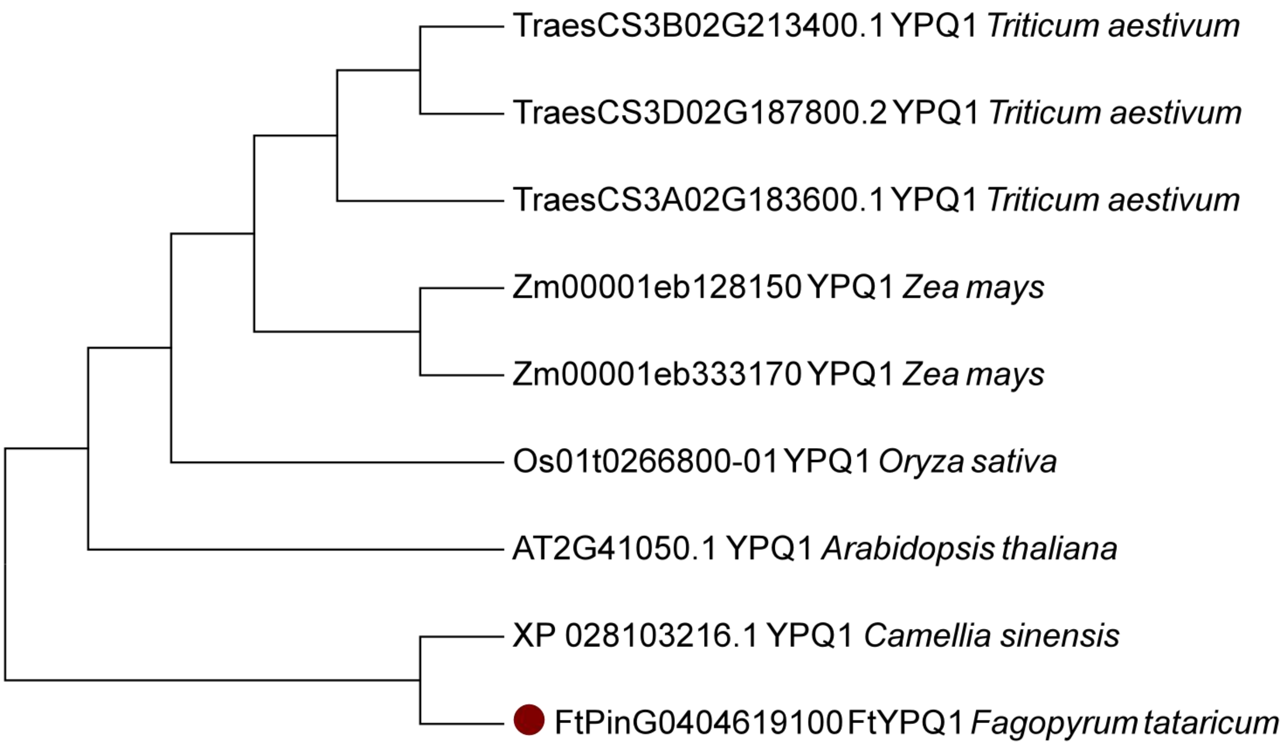

Figure S24. FtYPQ1 phylogenetic tree based on the neighbor joining method tree using full-length amino acid sequences of orthologues genes in Tartary buckwheat and other plants.

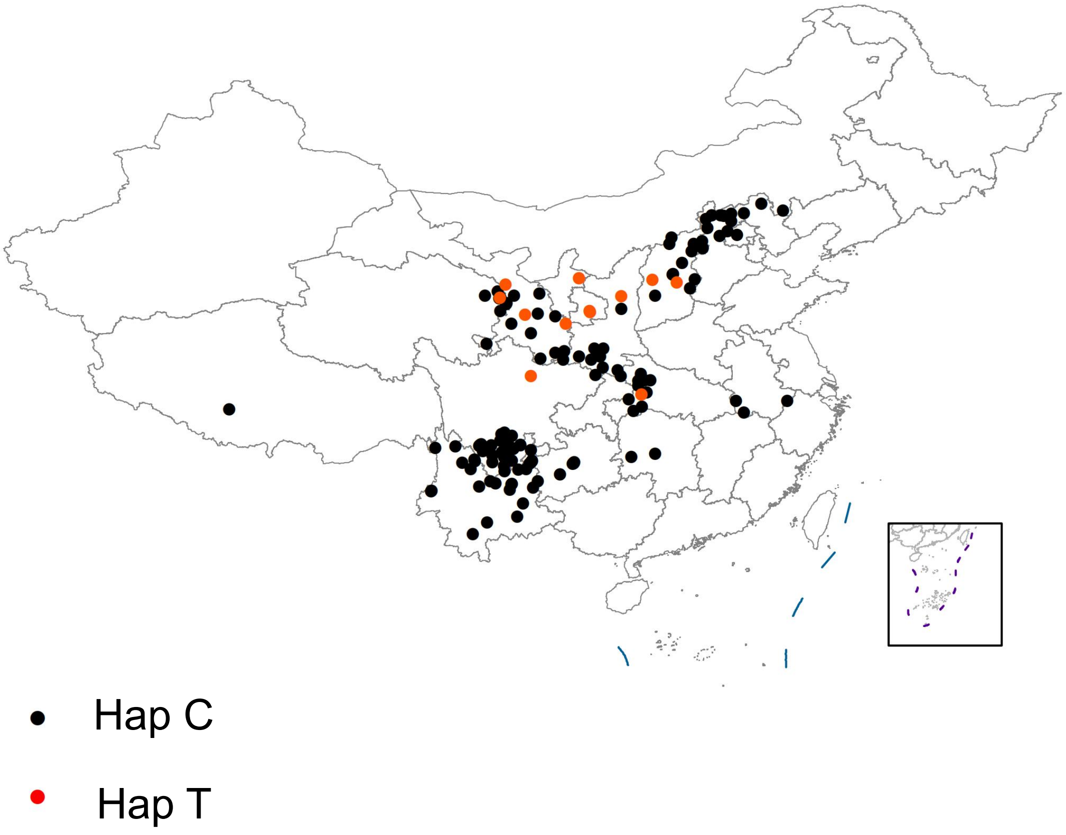

Figure S25. Geographic distribution of the Hap T (red) and Hap C (black) located in the promoter of *FtYPQ1* in Tartary buckwheat accessions.

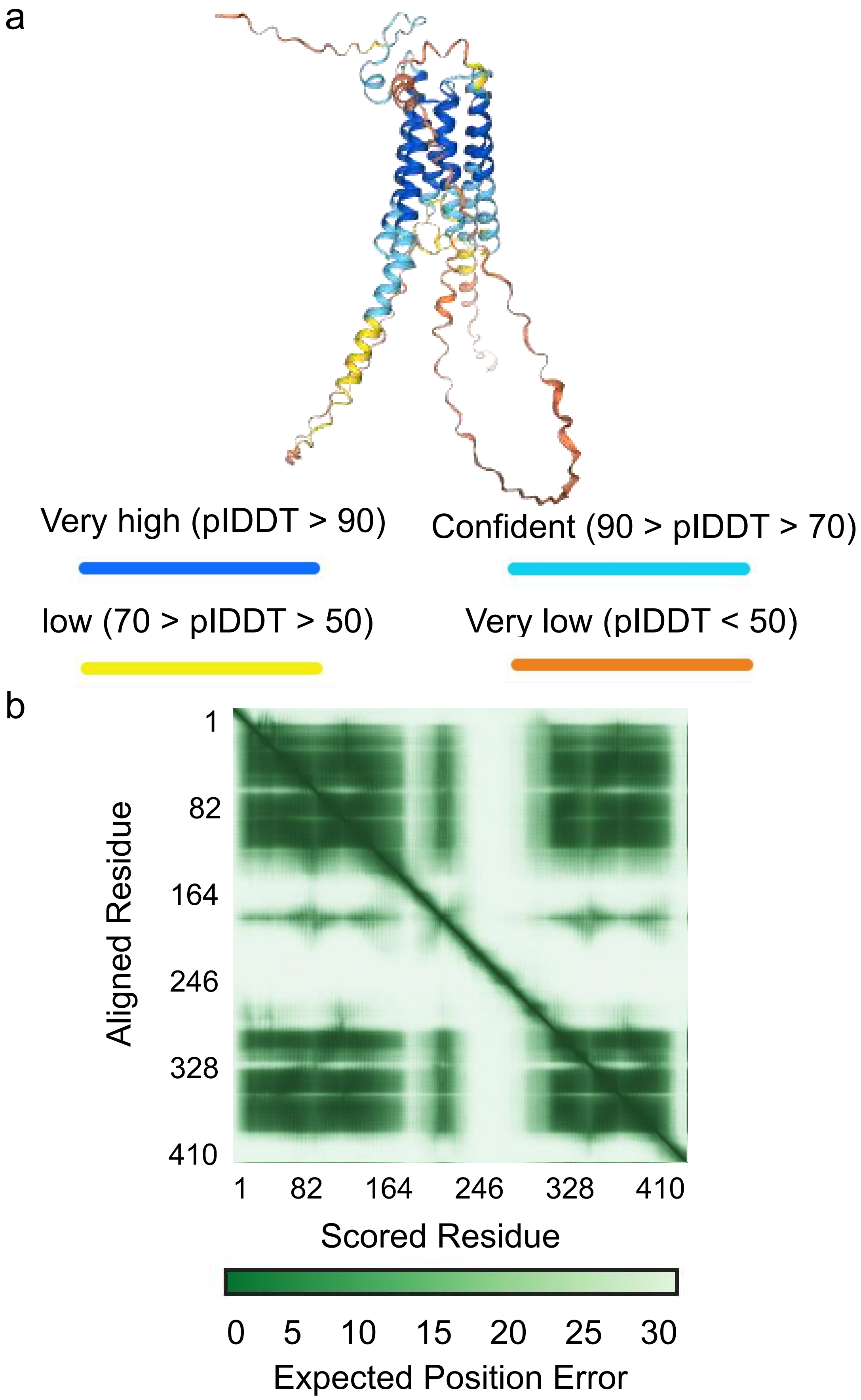

Figure S26. a. Predicted structure of FtYPQ1 colored by pLDDT (orange: 0-50, yellow: 50-70, cyan 70-90, and blue 90-100). b. Predicted Aligned Error (PAE) matrix of same prediction (darker is more confident).

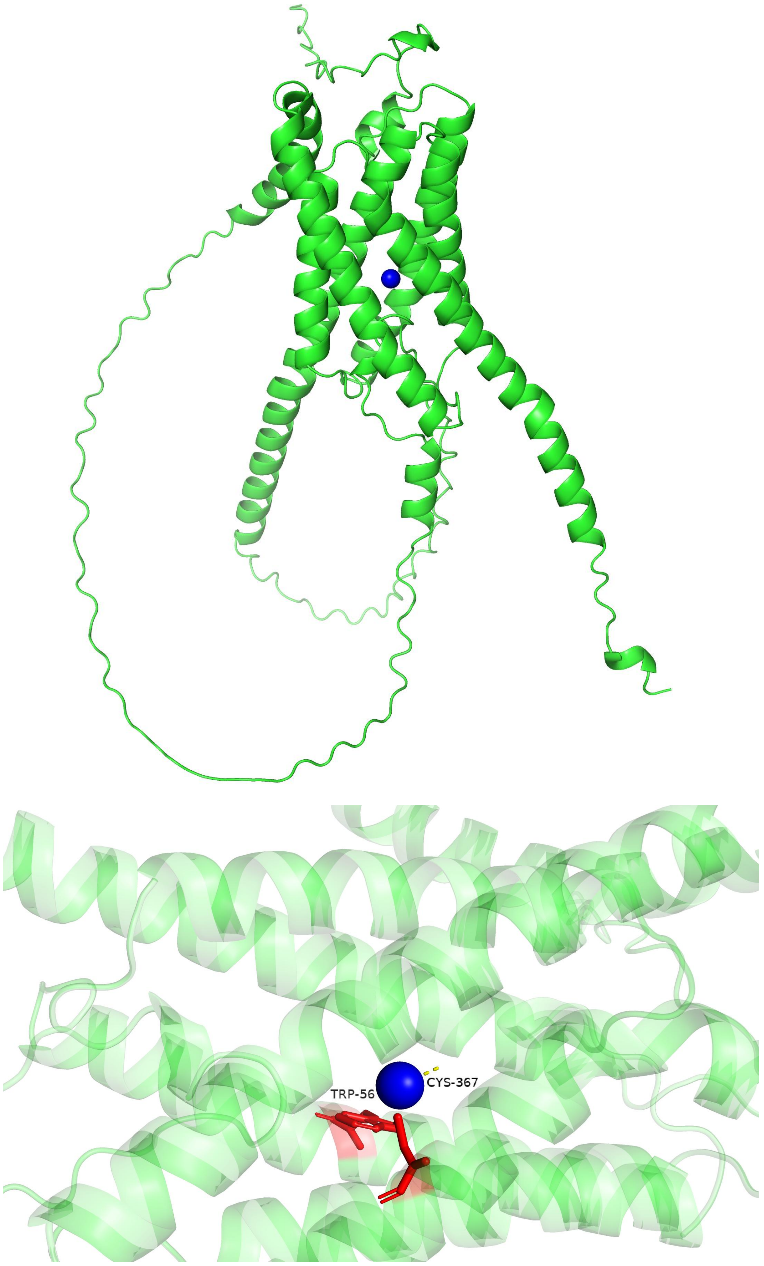

Figure S27. AlphaFold 3 prediction of FtYPQ1 protein interaction with zinc ion.

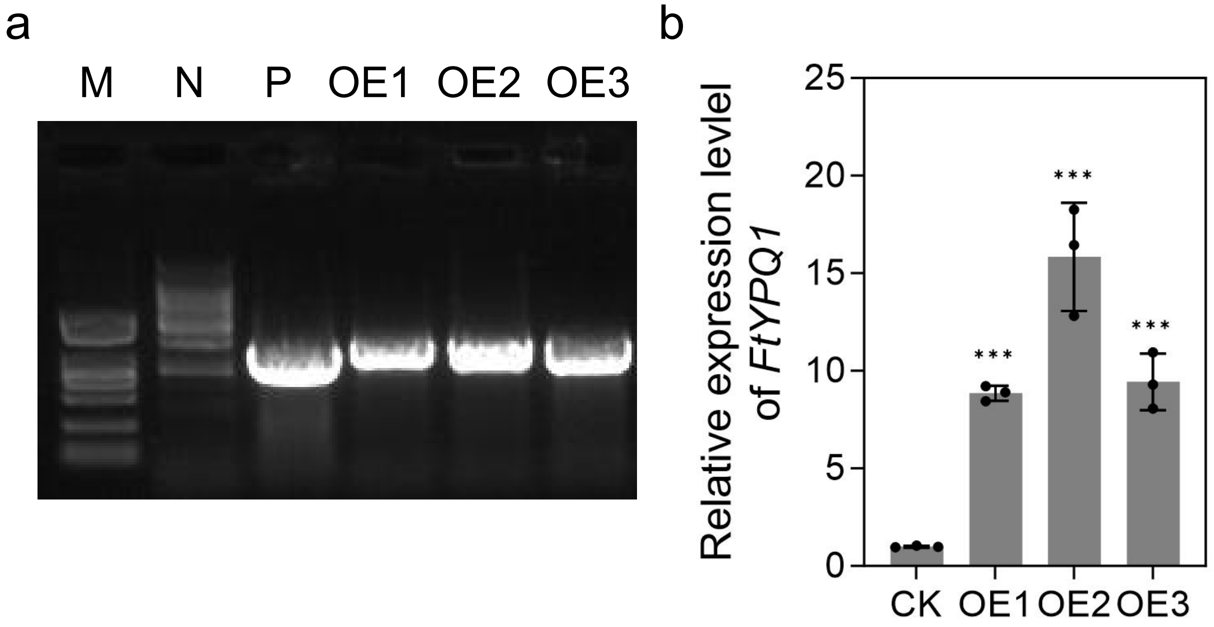

Figure S28. PCR (a) and RT-qPCR (b) analysis of hairy root lines expressing *FtYPQ1*. The hairy roots from wild type were used as negative control. N: negative control; P: positive control; M: DNA marker. The error bars indicate mean ± SD from three biological replicates. *** $P < 0.001$, two-tailed *t*-test.

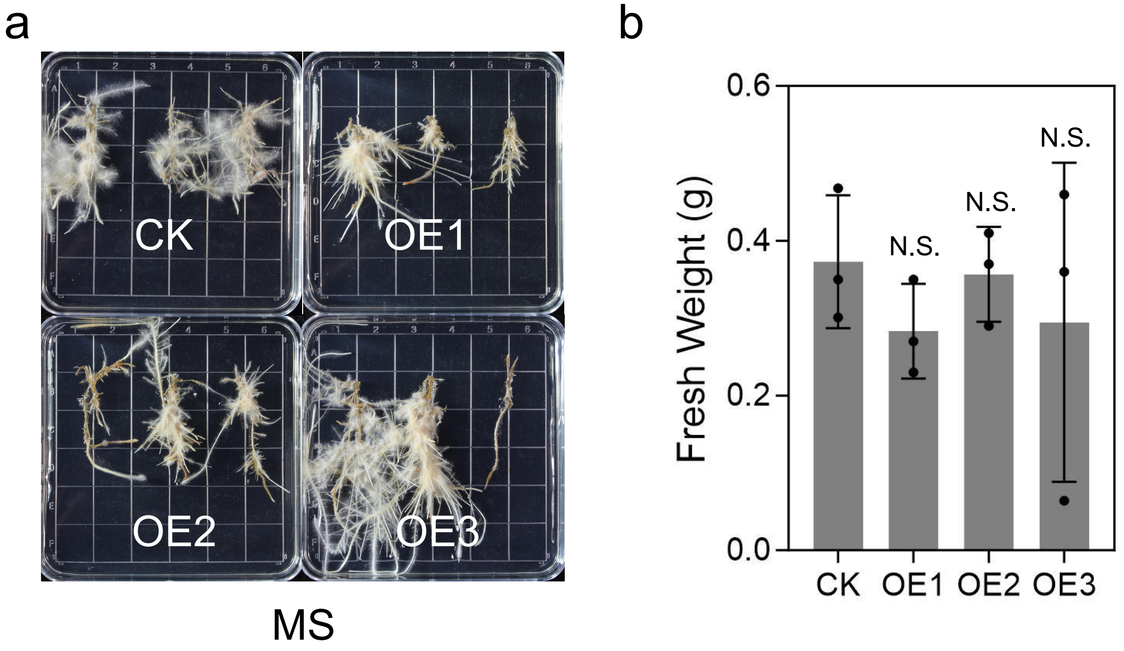

Figure S29. a. The hairy root phenotype grown on MS medium for 3 weeks. b. The fresh weight of different genotype hairy roots. The error bars indicate mean ± SD from three biological replicates. N.S. no significance, two-tailed *t*-test.

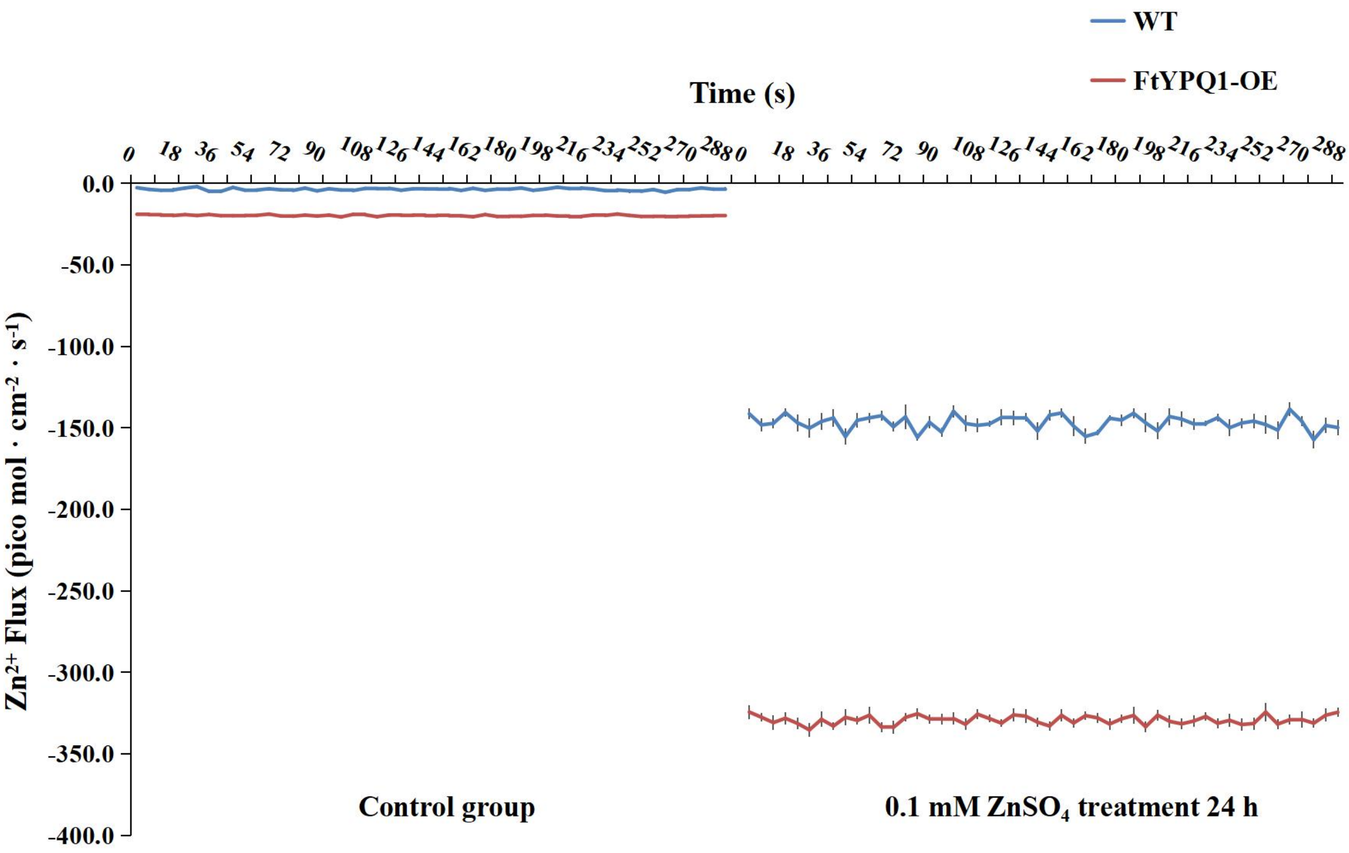

Figure S30. Comparison of extracellular zinc flux in living roots of *FtYPQ1* overexpression hairy roots and wild type. The control group indicates no treatment. The error bars indicate mean ± SD from six biological replicates.

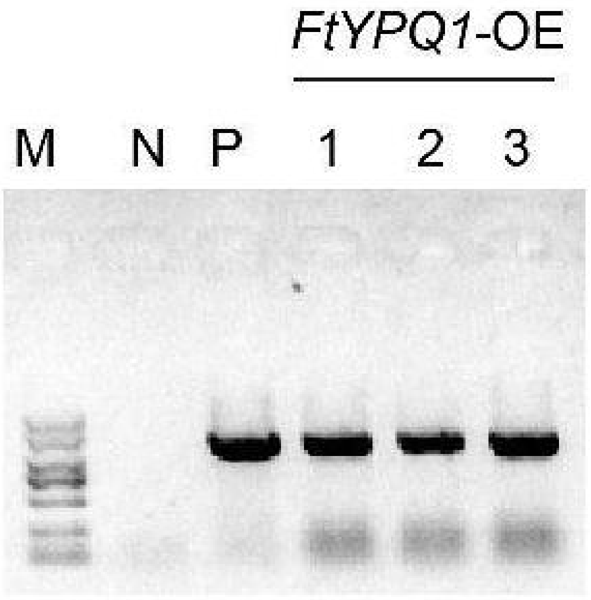

Figure S31. PCR analysis of *Arabidopsis* lines heterologously expressing *FtYPQ1*. The leaves from wild type (Col-0) were used as negative control. N: negative control; P: positive control; M: DNA marker.

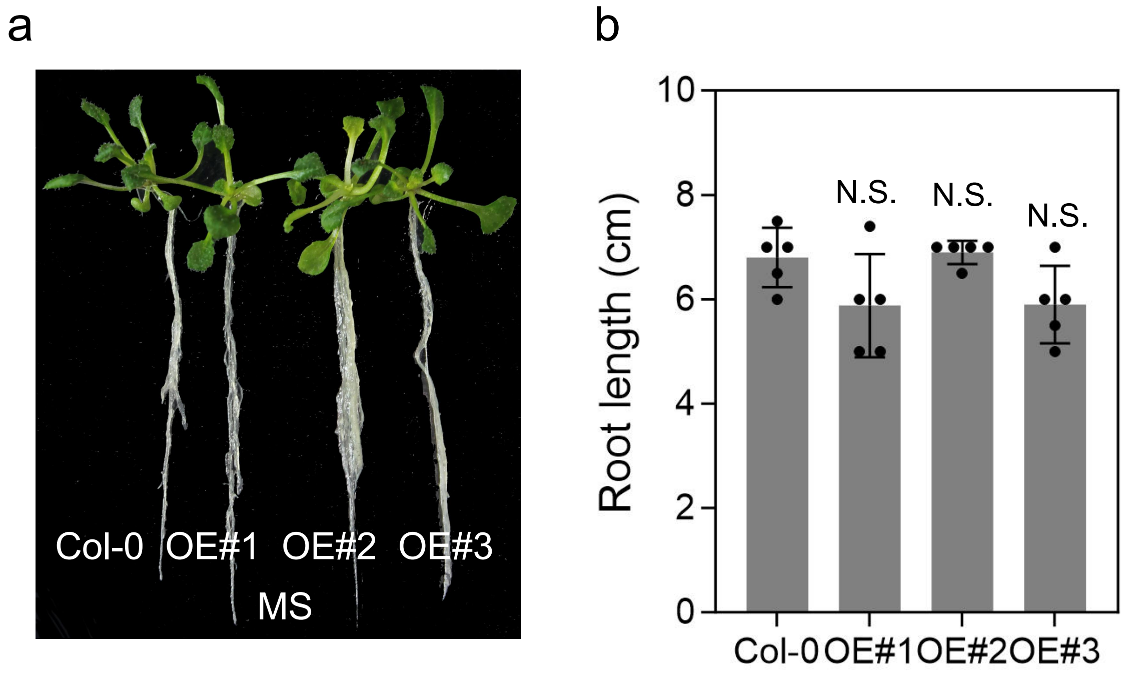

Figure S32. a. The phenotype of different genotype *Arabidopsis* lines grown on MS medium for 3 weeks. b. The root length of *Arabidopsis* lines. The error bars indicate mean ± SD from five biological replicates. N.S. no significance, two-tailed *t*-test.

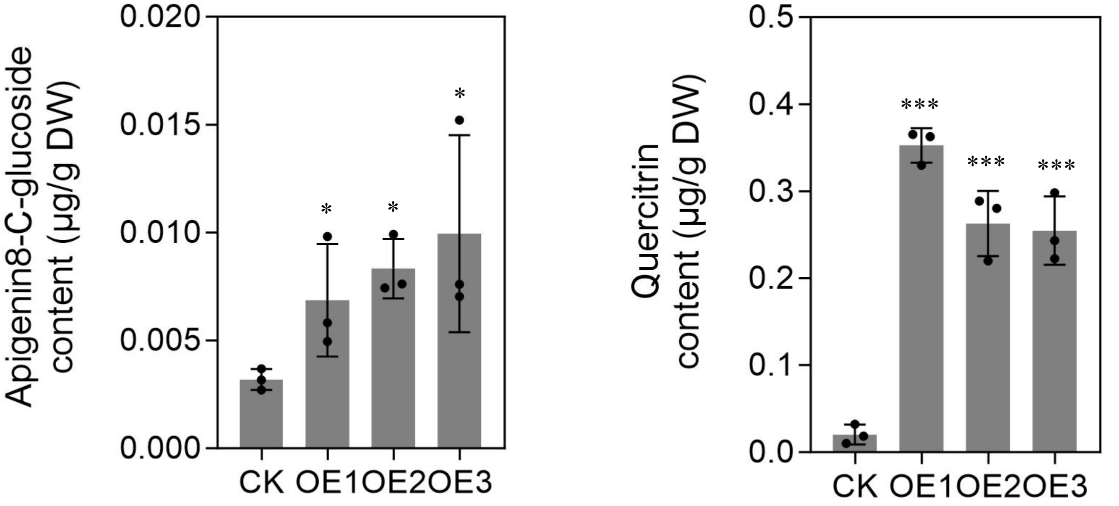

Figure S33. Apigenin-8-C-glucoside and quercitrin contents in different genotype hairy roots. The error bars indicate mean ± SD from three biological replicates. Asterisk (*) and (***) indicate significant difference at $P < 0.05$ and $P < 0.001$ using two-tailed *t*-test.

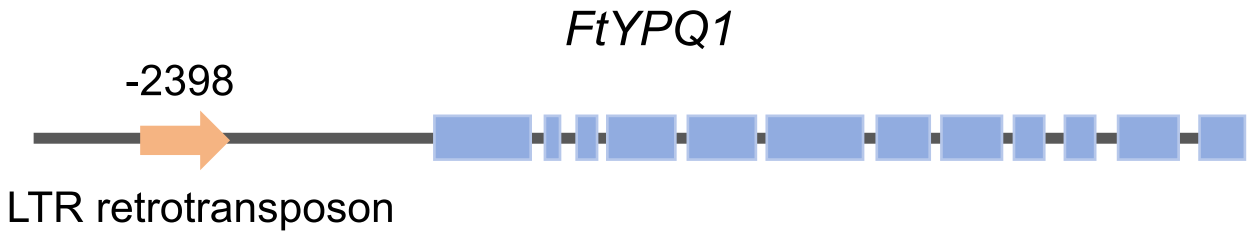

Figure S34. The position of LTR retrotransposon located in the promoter of *FtYPQ1* in Pinku.

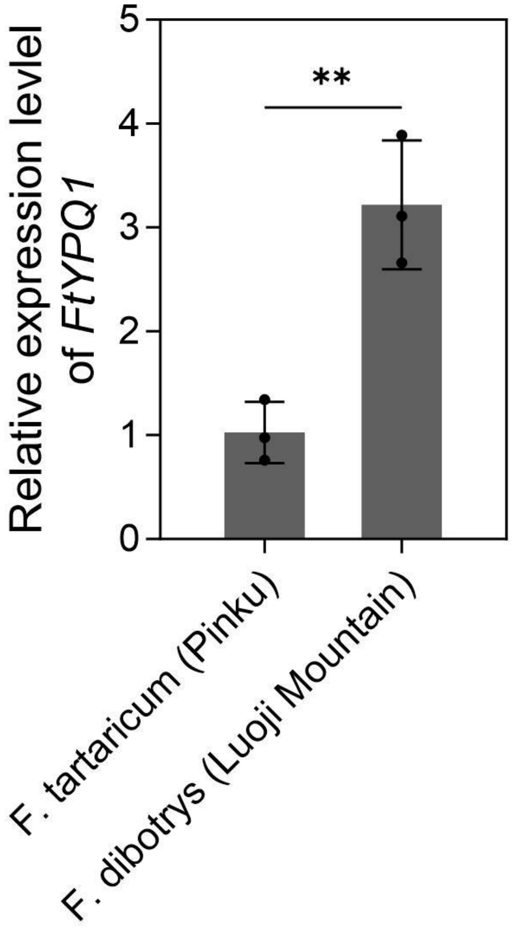

Figure S35. The expression level of *FtYPQ1* between *F. tataricum*-Pinku and *F. dibotrys*-Luoji Mountain. The error bars indicate mean ± SD from three biological replicates. ** $P < 0.01$, two-tailed *t*-test.

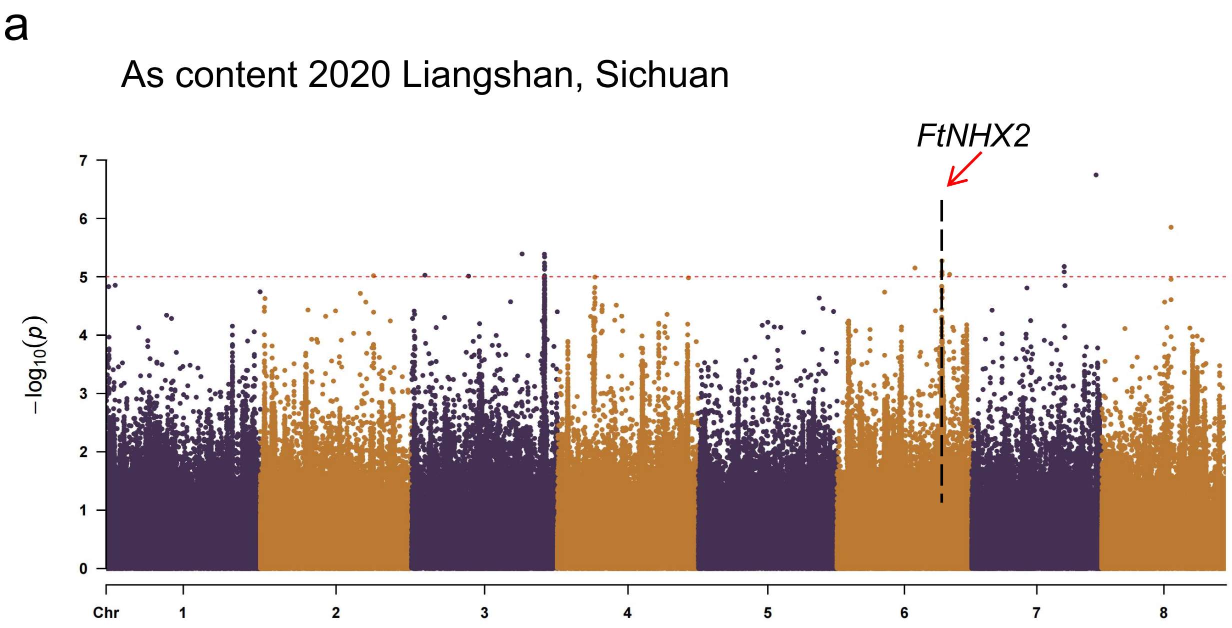

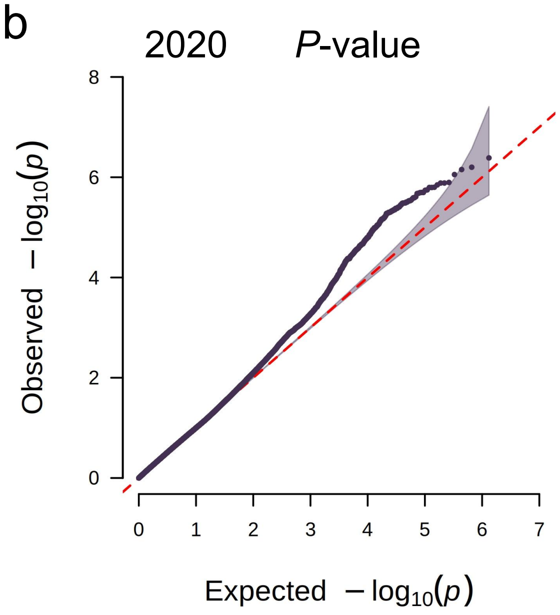

Figure S36. Manhattan and QQ plots of GWAS results of As content of Tartary buckwheat.

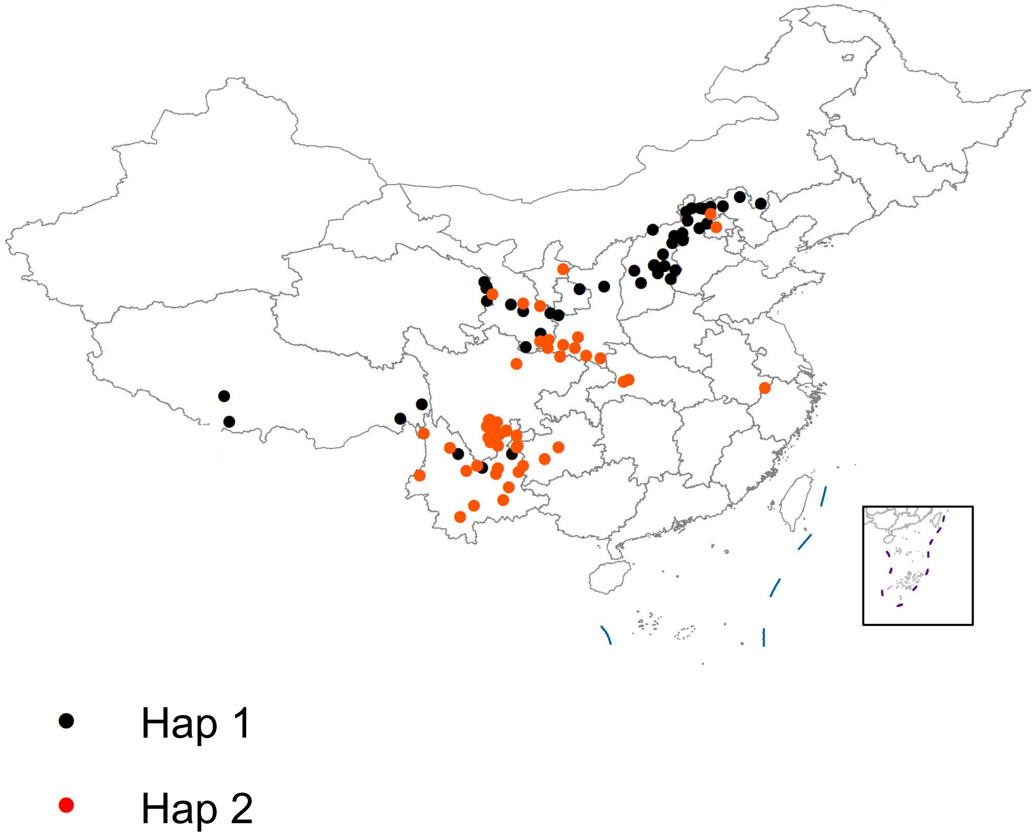

Figure S37. Geographic distribution of the Hap 2 (red) and Hap 1 (black) located in the promoter of *FtNHX2* in Tartary buckwheat accessions.

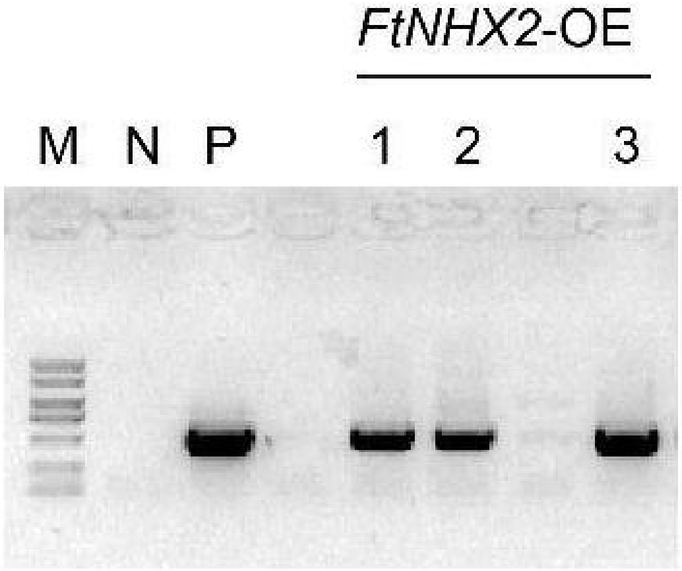

Figure S38. PCR analysis of *Arabidopsis* lines heterologously expressing *FtNHX2*. The leaves from wild type (Col-0) were used as negative control. N: negative control; P: positive control; M: DNA marker.

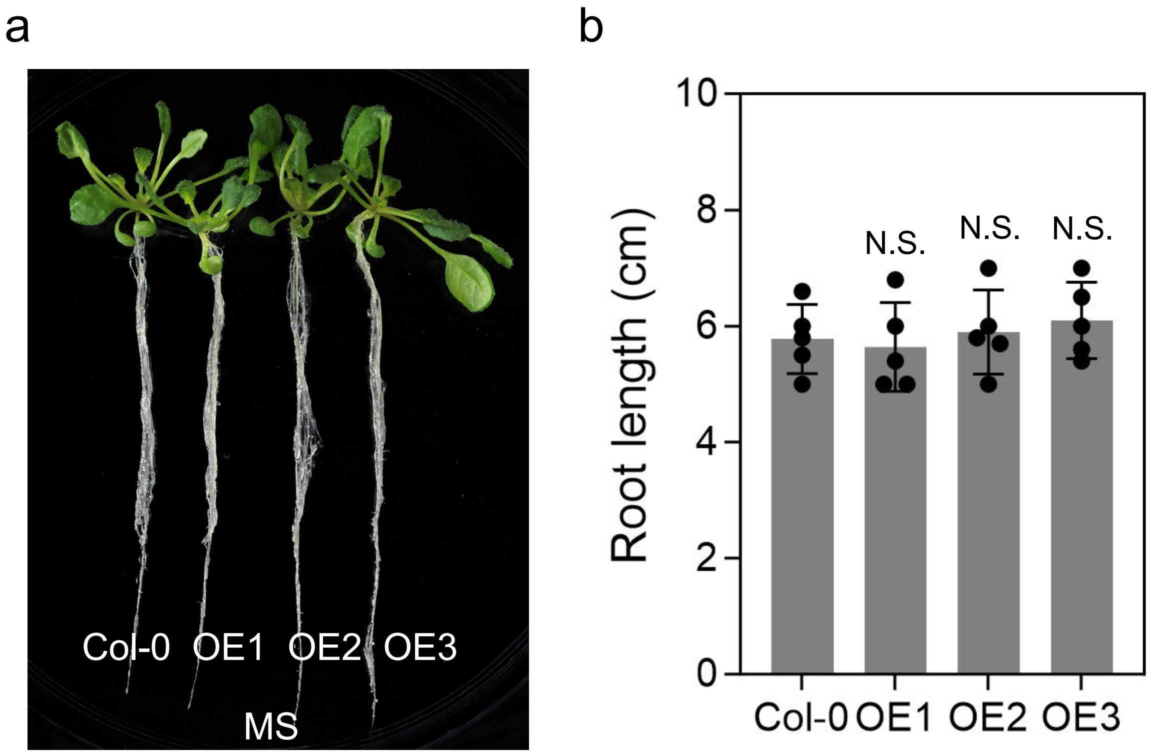

Figure S39. a. The phenotype of different genotype *Arabidopsis* lines grown on MS medium for 3 weeks. b. The root length of *Arabidopsis* lines. The error bars indicate mean ± SD from five biological replicates. N.S. no significance, two-tailed *t*-test.

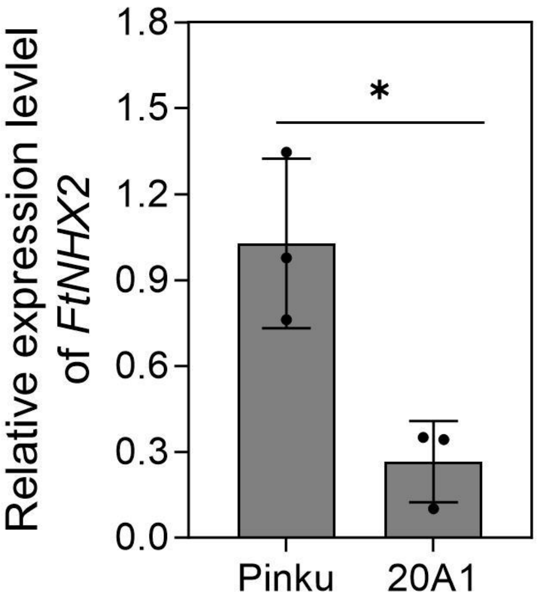

Figure S40. The expression level of *FtNHX2* between Pinku and 20A1 Tartary buckwheats. The error bars indicate mean ± SD from three biological replicates. * $P < 0.05$, two-tailed *t*-test.
